# Cardiomyocyte orientation recovery at micrometer scale reveals long‐axis fiber continuum in heart walls

**DOI:** 10.15252/embj.2022113288

**Published:** 2023-09-06

**Authors:** Drisya Dileep, Tabish A Syed, Tyler FW Sloan, Perundurai S Dhandapany, Kaleem Siddiqi, Minhajuddin Sirajuddin

**Affiliations:** ^1^ Centre for Cardiovascular Biology and Disease Institute for Stem Cell Science and Regenerative Medicine Bengaluru India; ^2^ The University of Trans‐Disciplinary Health Sciences and Technology (TDU) Bengaluru India; ^3^ School of Computer Science and Centre for Intelligent Machines McGill University, and MILA – Québec AI Institute Montréal QC Canada; ^4^ Quorumetrix Studio Montréal QC Canada

**Keywords:** 3D reconstruction, cardiomyocyte geometry, computer vision, fluorescent microscopy, heart wall structure, Cardiovascular System, Computational Biology, Methods & Resources

## Abstract

Coordinated cardiomyocyte contraction drives the mammalian heart to beat and circulate blood. No consensus model of cardiomyocyte geometrical arrangement exists, due to the limited spatial resolution of whole heart imaging methods and the piecemeal nature of studies based on histological sections. By combining microscopy and computer vision, we produced the first‐ever three‐dimensional cardiomyocyte orientation reconstruction across mouse ventricular walls at the micrometer scale, representing a gain of three orders of magnitude in spatial resolution. We recovered a cardiomyocyte arrangement aligned to the long‐axis direction of the outer ventricular walls. This cellular network lies in a thin shell and forms a continuum with longitudinally arranged cardiomyocytes in the inner walls, with a complex geometry at the apex. Our reconstruction methods can be applied at fine spatial scales to further understanding of heart wall electrical function and mechanics, and set the stage for the study of micron‐scale fiber remodeling in heart disease.

## Graphical abstract

A new method that combines confocal microscopy with computer vision provides unprecedented spatial resolution of cardiomyocyte geometry along the mouse heart wall.

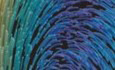

## Introduction

The mammalian heart wall is densely packed with cardiomyocytes that are geometrically aligned end‐on‐end to constitute cardiac muscle (Cretoiu *et al*, 2018). The geometric organization of cardiomyocytes supports synchronous contraction and electrical conduction while also offering mechanical strength (Streeter *et al*, 1969; LeGrice *et al*, 1995; Gilbert *et al*, 2007; Young & Panfilov, 2010; Savadjiev *et al*, 2012; Aumentado‐Armstrong *et al*, 2018; Libby *et al*, 2018). Malformations in cardiomyocyte arrangement and heart tissue fibrosis can lead to pathological conditions of the heart, including cardiomyopathies, remodeling following myocardial infarction, and disorders related to electrical propagation (Geerts‐Ossevoort *et al*, 2001; Chen *et al*, 2003; von Deuster *et al*, 2016). At a coarse spatial scale, the geometric organization of cardiomyocytes has been described as a helical continuum, wrapping around the chambers of the heart (Sallin, 1969; Streeter *et al*, 1969; Scollan *et al*, 1998; Beg *et al*, 2004; Chen *et al*, 2005; Gilbert *et al*, 2007; Rohmer *et al*, 2007; Bayer *et al*, 2012; Savadjiev *et al*, 2012; Libby *et al*, 2018). However, several competing models of cardiac myofiber organization still exist (Gilbert *et al*, 2007; Anderson *et al*, 2009), including a three‐layer model (Rushmer *et al*, 1953) and characterizations as nested donuts (Streeter *et al*, 1969), toroids (Peskin, 1989), pretzels (Jouk *et al*, 2000), or a single helical band (Corno *et al*, 2006).

Despite this lack of consensus, the present models of heart wall cardiomyocyte geometry in use for cardiovascular research are largely derived from millimeter resolution diffusion‐tensor magnetic resonance imaging (DT‐MRI; Peskin, 1989; Horowitz *et al*, 1993; Helm *et al*, 2005; Gilbert *et al*, 2007; Savadjiev *et al*, 2012). These models all support a smooth clockwise rotation of cardiomyocytes in a transmural penetration from outer to inner wall and do not predict any singularities in their aggregate orientation. Rule‐based models of cardiac myofiber orientation (Bayer *et al*, 2012) as well as those based on minimal surfaces (Savadjiev *et al*, 2012) also exist, but these are largely consistent with those afforded by DT‐MRI. DT‐MRI has also been used to obtain statistical atlases of heart wall myofiber geometry from the imaging of multiple subjects (Peyrat *et al*, 2007; Lombaert *et al*, 2012). Whereas such models can advance studies of heart wall electrical and mechanical function (Vetter *et al*, 2005; Young & Panfilov, 2010), they all suffer from limitations in spatial resolution. In fact, hundreds of cardiomyocytes can occupy a single voxel at this millimeter scale.

Micron‐scale light microscopy methods have the potential to recover orientation at the scale of individual cardiomyocytes. However, such efforts have concentrated thus far on imaging small sections (Seidel *et al*, 2016) or 3D stacks of heart tissue (Pope *et al*, 2008; Sivaguru *et al*, 2015; Nehrhoff *et al*, 2017; Perbellini *et al*, 2017; Teh *et al*, 2017; Garcia‐Canadilla *et al*, 2019; Merz *et al*, 2019) and not on modeling cardiomyocyte or myofiber geometry at the organ scale. Approaches based on histological sections are typically limited to two‐dimensional imaging (Greenbaum *et al*, 1981; Young *et al*, 1998; Anderson *et al*, 2009). Such methods also have not recovered the orientation of individual cardiomyocytes or their aggregate orientation at the whole heart scale. Thus, the geometric organization of cardiomyocytes at the micron scale across entire heart walls remains a fundamental unaddressed question in organ biology.

To tackle this problem, we developed new tissue structure orientation analysis methods by combining confocal light microscopy‐based deep and wide imaging with computer vision techniques. Our methods extract information from the fluorescence signal at cardiomyocyte boundaries, where the intensity gradient provides unbiased estimates of the eigenvectors associated with the structure tensor. This allowed us to estimate micron‐scale cardiomyocyte orientations across entire long‐axis and short‐axis ventricular sections from mouse hearts. Our three‐dimensional reconstructions at unprecedented spatial resolution revealed never before reported long‐axis bands of fibers extending along the entire length of the outer ventricular walls, different from the known smoothly varying helical arrangements (Scollan *et al*, 1998; Chen *et al*, 2005; Gilbert *et al*, 2007; Bayer *et al*, 2012; Savadjiev *et al*, 2012). This finding could advance present understanding of heart wall mechanics and electrical conduction, as well as open new opportunities to interrogate heart wall tissue structure and diseases related to alterations of cardiomyocyte and hence myofiber organization, across a range of spatial scales.

## Results

### An integrated pipeline for estimating micron‐scale cell orientations from *ex vivo* tissue

**Figure 1:**
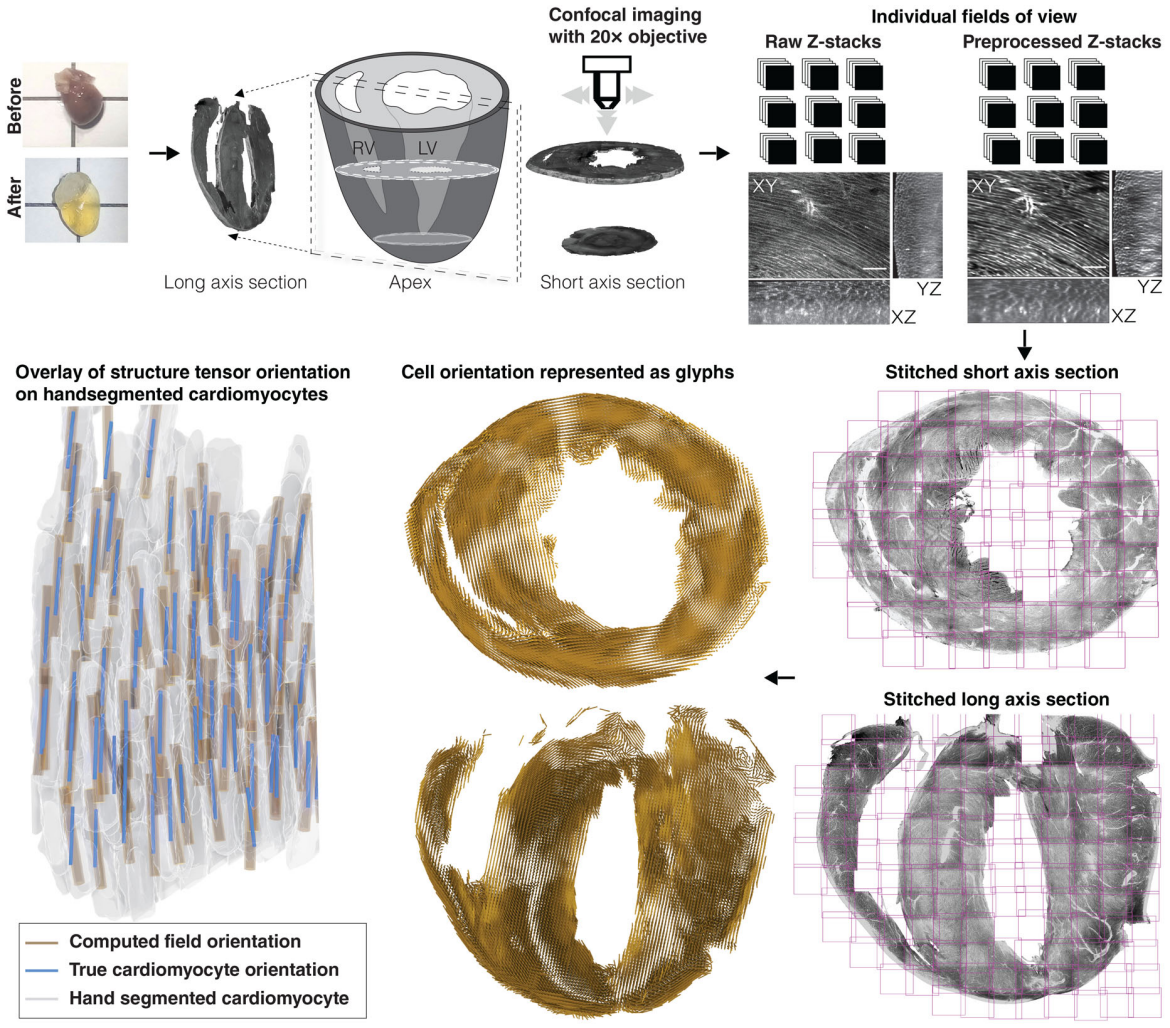
Overview of the heart tissue preparation, imaging and analysis pipeline *Top panels from left to right*: Representative photographs of a mouse heart before and after applying the CLARITY procedure (Tomer *et al*, 2014). An illustration of the cleared mouse heart sections analyzed in this study. The long‐axis (LA) sections correspond to longitudinal dissections of the mouse heart through a transverse plane equivalent to the HLA‐4C views of the AHA (or echocardiogram) nomenclature, revealing the right and left ventricular chambers. The short‐axis (SA) sections are dissected at the midventricular plane, equivalent to PSAX‐PML views of the AHA (or echocardiogram) nomenclature (Cerqueira *et al*, 2002). At least one mouse heart in this study provided four continuous sections, each being approximately 300 μm in thickness, for both the LA and SA analyses (Appendix Table S1; Materials and Methods). After sectioning and WGA staining, the tissue slices were imaged using confocal microcopy spanning the entire length, breadth, and width of the LA and SA sections (Materials and Methods). Using custom‐built algorithms, the individual confocal stacks were preprocessed for contrast enhancement, denoised, and then stitched (Materials and Methods). *Bottom panels from right to left*: The imaging and preprocessing pipeline results at a 2‐μm voxel isometric resolution of the entire SA and LA sections, up to a depth of about 300 μm in thickness. The estimated structure tensor vectors are visualized as glyphs represented as golden yellow lines for the entire SA and LA sections (Materials and Methods). A representative 3D view (bottom left) of the hand‐segmented cardiomyocytes (gray) from the WGA stain, overlayed with the cell orientations estimated using the true myocyte orientation (purple) and the estimated field orientation (golden yellow) by the structure tensor method (Materials and Methods).

**Figure EV1:**
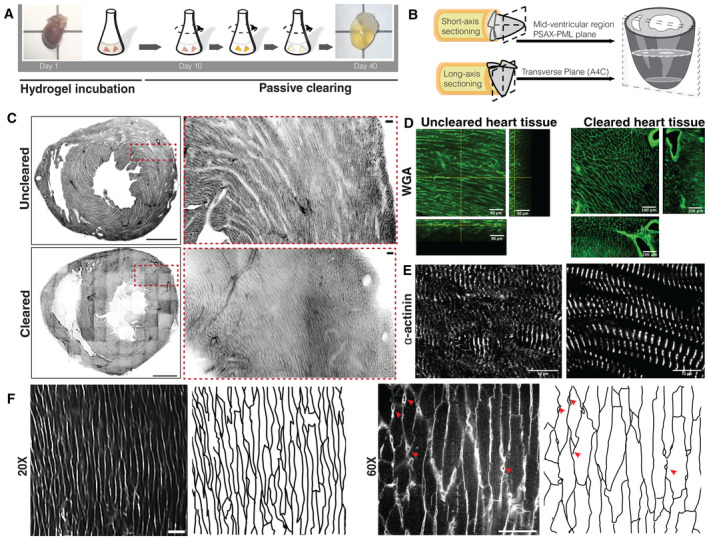
A comparison of cleared and uncleared mouse heart tissue by imaging A
A schematic illustration of the CLARITY method. The harvested heart tissues are incubated in a hydrogel/PFA mixture at 4°C for approximately 9 days. Following hydrogel incubation, the solution mixture with the heart tissues is polymerized at 37°C. The heart tissues are then excised from the polymerized hydrogel and shaken at 37°C with a clearing solution until they attain a desirable level of transparency (see Materials and Methods for details).B
The clarified heart is sectioned along its short‐ or long‐axis. For the short‐axis, a midventricular region that approximates the PSAX‐PML (parasternal short‐axis—papillary muscle level) plane was chosen. For the long‐axis, a transversal plane that represents the A4C (apical four chambers) plane was used.C–E
A comparison of uncleared and cleared heart tissue sections stained with WGA and the alpha‐actinin antibody.F
A comparison of 20× (2‐μm isotropic resolution) and 60× (0.663 * 0.663 * 0.79 μm^3^ x, y, and z resolution, respectively) cleared heart tissue sections stained with WGA. The raw and skeletonized images are shown for each magnification. The visible cell boundaries that are not of cardiomyocytes are marked with arrow heads (red) in the 60× images. The scale bar is 1,000 and 50 μm for the full view and zoomed in regions, respectively, in the WGA uncleared and cleared tissue images. The scale bar for the alpha‐actinin images is 10 μm.

To employ fluorescence based deep tissue imaging, we applied a tissue clearing method based on the CLARITY protocol (Tomer *et al*, 2014), optimizing it for *ex vivo* heart tissue (Materials and Methods; Figs 1 and EV1A). The intact cleared hearts from normal mice were serially sectioned in short‐ and long‐axis views (Fig EV1B), approximately at the parasternal short axis‐papillary muscle (PSAX‐PML) and the horizontal long axis‐4 chamber (HLA‐4C) regions, respectively, following the American Heart Association (AHA) nomenclature (Cerqueira *et al*, 2002). Post sectioning, the tissue was stained with fluorescent wheat germ agglutinin (WGA), which marks cell membranes (Materials and Methods) (Figs 1 and EV1C and D). The WGA‐stained tissue sections were subjected to confocal imaging (Methods) (Fig 1). A comparison with uncleared heart images showed no significant alterations in the tissue due to clearing procedures (Fig EV1D and E). The images were acquired at 2‐μm isotropic resolution such that only cardiomyocyte boundaries were captured (Fig EV1F). Every intact short‐ and long‐axis section was imaged in its entirety, as blocks of individual fields of view, each being ~ 640 × 640 × 300 μm in dimension (Materials and Methods) (Fig 1; Appendix Fig S1A). The images from cleared heart tissues were further processed by deconvolution, denoising, and stitching algorithms to recover micron‐scale resolution for each field of view and then stitched to obtain a full view of the entire section (Fig 1; Appendix Fig S1B–E).

**Figure EV2:**
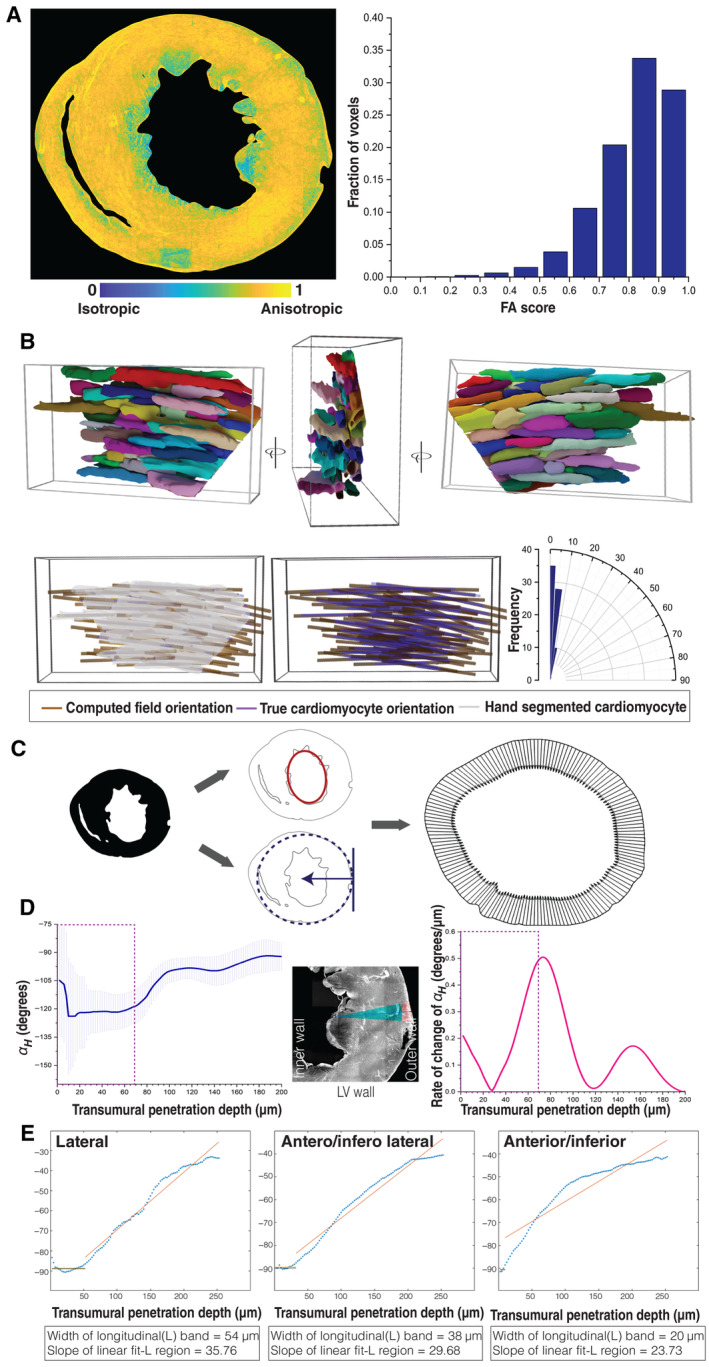
The structure tensor method for orientation estimation A
Fractional anisotropy scores are shown using a parula colormap (left) with a value in the range 0–1. The histogram (right) shows the fraction of pixels in each respective FA bin. The majority of the pixels have a high FA score, indicating the presence of dominant local orientations in the tissue stack. The color bar for the FA score is as indicated.B
Representative 3D views of hand‐segmented cardiomyocytes with randomly assigned colors to each cell (top panel). A representative 3D view of ground truth orientation (purple) based on the second moment matrix for hand‐segmented myocytes (gray) on the left, with the estimated field orientation from the WGA image (golden yellow) using a structure tensor approach (Materials and Methods) in the middle (bottom panel). The magnitude of the difference between the ground truth and the estimated orientation in degrees is illustrated with a graph on the right. The mean difference between two ground truth and estimated orientations is 5.98° *±* 2.3°.C
Helix angle calculation: Masking (left) followed by centroid estimation (middle, top); Masking the short‐axis section and estimating the tangent plane and normal for the penetration axis (middle, bottom). The set of penetration directions is shown on the right, from outer to inner wall.D
αH plot (left), region of LV wall analyzed (middle) and rate of change of αH calculated over a neighborhood of 15 voxels. The region marked by a dashed box represents the outer wall longitudinal cells, where the rate of change of αH is initially small and then increases sharply as one approaches the middle wall region, after which it plateaus and then increases again.E
Line fits for αH plots were calculated for the lateral, antero/infero lateral, and anterior/inferior regions, with the extent of the outer wall longitudinal cells shown by length of the first line in each plot.

A notable feature of the WGA‐stained stitched images is the appearance of a distinct pattern of cells restricted to a particular geometric location (Fig EV1C). To quantitatively assess the cell patterns, we estimated the orientations at the scale of individual cardiomyocytes using a structure tensor method (Granlund & Knutsson, 1994; Materials and Methods; Figs 1 and EV2A). The fractional anisotropy colormap and the distribution of anisotropy values across the short‐axis section illustrate the output of our automated structure tensor method for estimating the longest axis of a cardiomyocyte (Fig EV2A). To further validate our numerically obtained structure tensor orientation estimates, we compared them with the orientations of a selection of hand‐segmented cardiomyocytes and found there to be a strong agreement between the two (mean difference 5.98° ± 2.3°; Figs 1 and EV2B).

### Visualizing cell orientations using glyphs, streamlines, and angular projections

In order to visualize the estimated cardiomyocyte orientations, we adopted the use of glyphs and streamlines, that are often used to convey orientation estimates from DT‐MRI data (Materials and Methods; Rohmer *et al*, 2007; Leemans, 2010). For visualizing the local orientation of each cardiomyocyte at the submicron voxel scale, we display every 12^th^ voxel as an individual glyph (Fig 1; Movie EV1). We used bidirectional streamlines as a proxy for visualizing the aggregate cardiomyocyte orientations across the heart wall in both short‐ and long‐axis sections. Our micron‐scale 3D recovery of cardiomyocyte orientation across entire thick tissue sections of the heart represents a significant advance in recovering the geometric organization of cardiomyocytes since our reconstructions are at a much higher spatial resolution than that provided by previous studies (Gilbert *et al*, 2007; Materials and Methods; Fig 1; Movie EV1).

**Figure 2:**
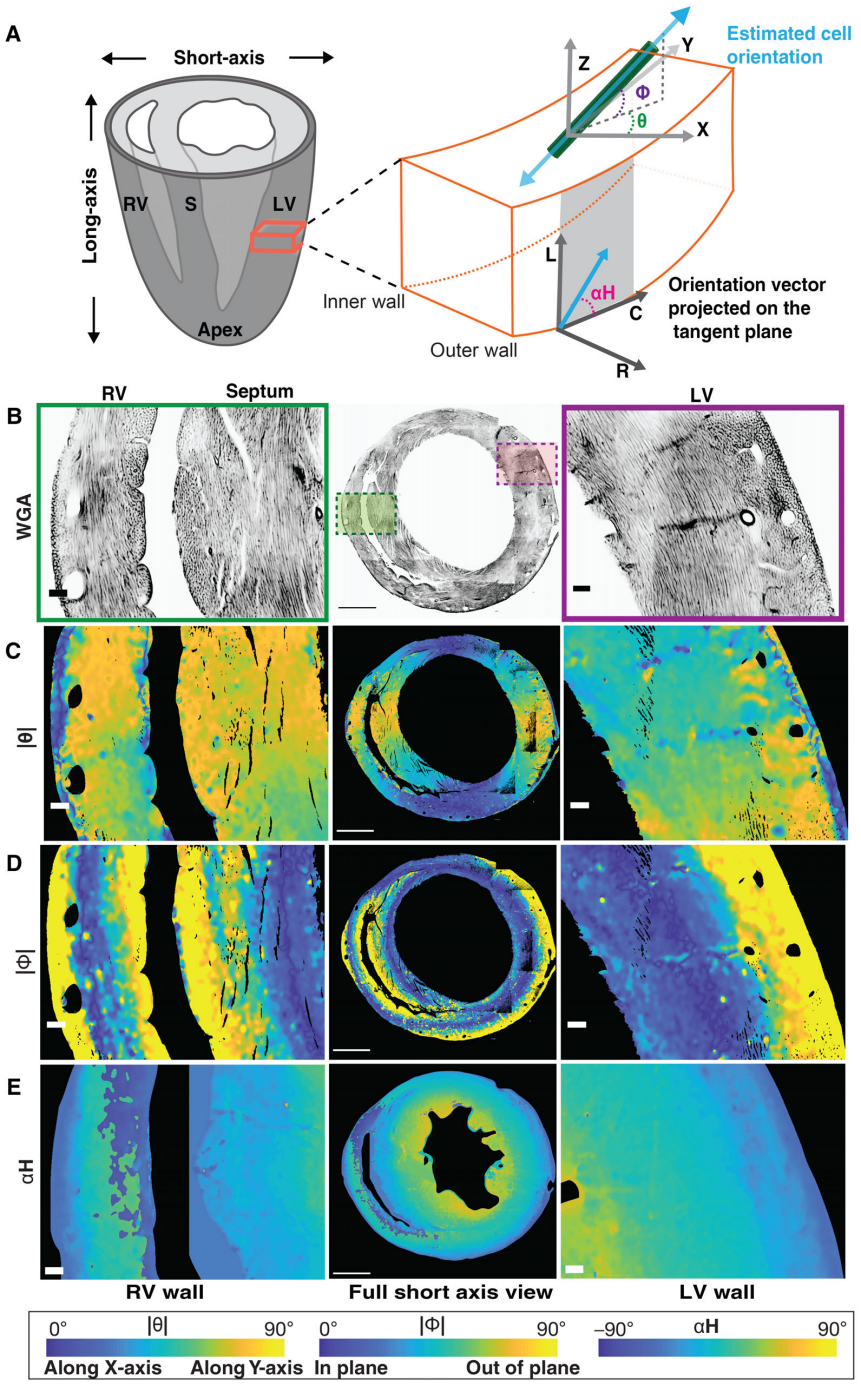
Cell orientation across a midventricular short‐axis section of the heart A
An illustration of the angles measured to represent the cell orientations across the ventricular walls. The red box represents a magnified view of the ventricle wall, with the global axes and labels as indicated: C‐Circumferential L‐Longitudinal R‐Radial. The green cylinder and the blue bidirectional arrow represent the cardiomyocyte and its long‐axis, that is, the estimated cell orientation. Φ is the angle between the projection of the estimated cell orientation onto the XY plane and the estimated cell orientation. θ is the angle between the projection of the estimated cell orientation onto the XY plane and the X‐axis direction. αH, the helix angle is the angle between the projection of the cell orientation onto the plane perpendicular to the transmural penetration direction and the circumferential direction.B–E
A magnified view of the right (green rectangle) and the left (magenta rectangle) ventricular regions for a full view of the WGA stain (middle). The |θ|, |Φ|, and αH angles for the mouse SA sections are shown using a parula colormap. The yellow tones for the |θ| angle represent cell orientation along the global Y‐Axis while the blue tones represent cell orientation along the X‐axis. For |Φ|, the blue and yellow tones represent cells with orientations aligned with the short‐axis section, and orthogonal to it, respectively. The colormaps scale with the angles, as indicated. The scale bar for the full view images is 1,000 μm and for the magnified views is 100 μm.

To quantitively analyze the estimated cardiomyocyte orientations, we plotted them using three angles: Theta (θ), Phi (Φ), and the Helix angle ($\alpha_{H}$; Materials and Methods; Fig 2A). θ represents the acute angle between the projection of the estimated cell orientation (myofiber orientation) onto the short‐axis (XY) plane and the X‐axis. Φ represents the acute angle between the estimated cell orientation and the short‐axis (XY) plane in the heart tissue. $\alpha_{H}$ is the angle between the projection of the myofiber onto the local tangent plane to the heart wall and the circumferential direction (Fig 2A). This corresponds to the helix angle, which is widely used in a body of work on macro‐scale myofiber organization (Beg *et al*, 2004; Chen *et al*, 2005; Gilbert *et al*, 2007; Peyrat *et al*, 2007; Lombaert *et al*, 2012; Agger *et al*, 2020). Several studies have reported that $\alpha_{H}$ undergoes a smooth rotation along a penetration axis from epicardium to endocardium in the left ventricle (Beg *et al*, 2004; Chen *et al*, 2005; Gilbert *et al*, 2007). We therefore computed $\alpha_{H}$ using an estimate of the radial penetration direction at each location in the heart wall (Materials and Methods; Figs 2A and EV2C).

### Regimes of discrete cell orientations across ventricular walls

To demarcate the discrete cell arrangements across the ventricular walls (Fig 2B) and the boundaries between these different cell arrangements, we computed the Φ and θ angles defined in Fig 2A and visualized them using quantitative colormaps (Materials and Methods). The colormaps for the θ angle of a single plane in the short‐axis (XY) view show that the cardiomyocytes are arranged in a radial direction following a smooth continuum (Fig 2C). Our findings at the micron scale are thus consistent with previous reports that heart wall myofibers wind around the ventricles in a circumferential manner (Spotnitz, 2000; Chen *et al*, 2005; Gilbert *et al*, 2007; Savadjiev *et al*, 2012; Poveda *et al*, 2013) (Movies EV2 and EV3).

**Figure EV3:**
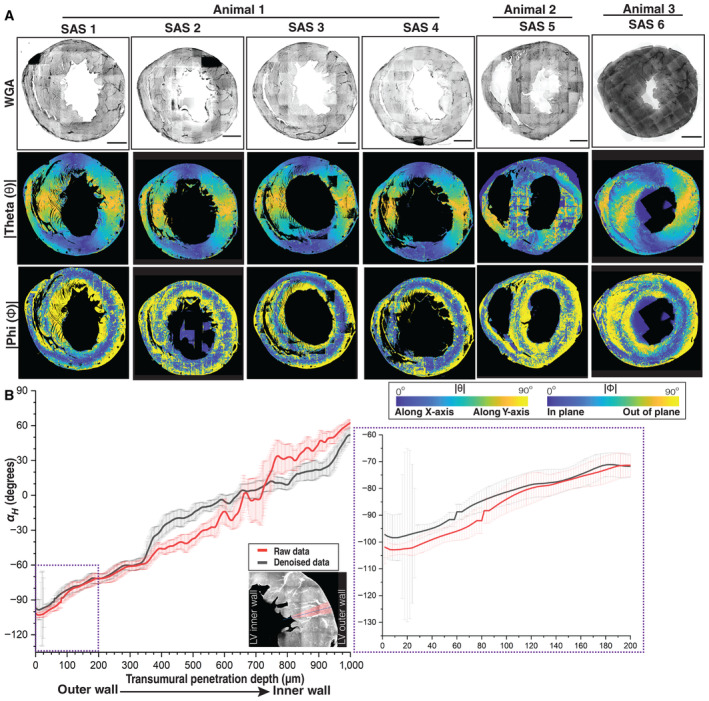
WGA staining and angular colormaps of different short‐axis sections A
A maximum intensity Z‐projection of the WGA‐stained short‐axis section from different mouse hearts as indicated, shown in grayscale, with the |Φ| and |θ| angles for cell orientations shown using parula colormaps, with the color bars as indicated. The scale bar is 1,000 μm.B
A comparison of αH estimates from raw (red line) and denoised short‐axis section image stacks (black line) are shown for comparison. The region of the LV wall analyzed is shown in the bottom left panel, with a zoom in on the first 200 μm on the bottom right. The transmural penetration direction is from the outer to the inner LV wall. The raw data agree with denoised αH estimates in most of the places; however, we observed that estimation was much more consistent and robust to nonuniform illumination changes with denoised data. The intensity between two fields of view (~ 350 μm in transmural penetration depth) and the αH estimates begin to deviate marking the edges.

**Figure EV4:**
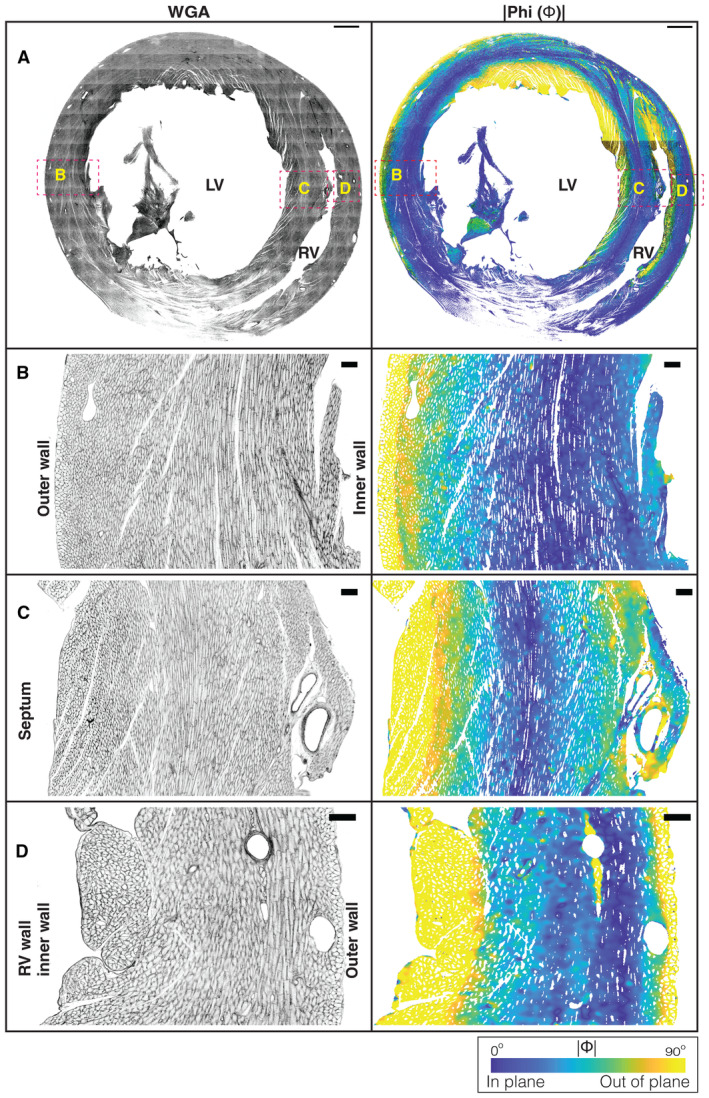
Analysis of a short‐axis section of a rat heart A
A short‐axis view of the ventricular chambers of a rat heart, sectioned at PSAX‐PML (parasternal short‐axis—papillary muscle level). A maximum intensity projection of the WGA stain is shown in grayscale, with the |Φ| angle shown using a parula colormap. The scale bar is 1,000 μm.B–D
Zoomed in regions from the left ventricle, septum and right ventricle, with the WGA stain shown in grayscale and the |Φ| angle shown using a parula colormap. The scale bars are 1,000 μm 100 μm for (A) and (B–D), respectively. The color bar is as indicated.

In contrast to θ, the Φ angle reveals a discrete and significant change in cardiomyocyte orientation across the ventricular walls (Fig 2D). The out‐of‐plane cardiomyocytes are approximately perpendicular to the short‐axis plane. This arrangement shown in yellowish tones in the colormaps forms a crescent shape along the outer walls of both ventricles and an orbicular shape in the inner walls surrounding both the chambers (Figs 2D, EV3A and EV4A–D). Taken together, the orientation reconstructions suggest that the out‐of‐plane cardiomyocytes at the edges of the ventricular walls have an orientation arrangement that is different from that of the well‐established circumferential heart fibers (Movie EV3).

### Sharp changes in myocyte orientation at ventricular wall boundaries

**Figure 3:**
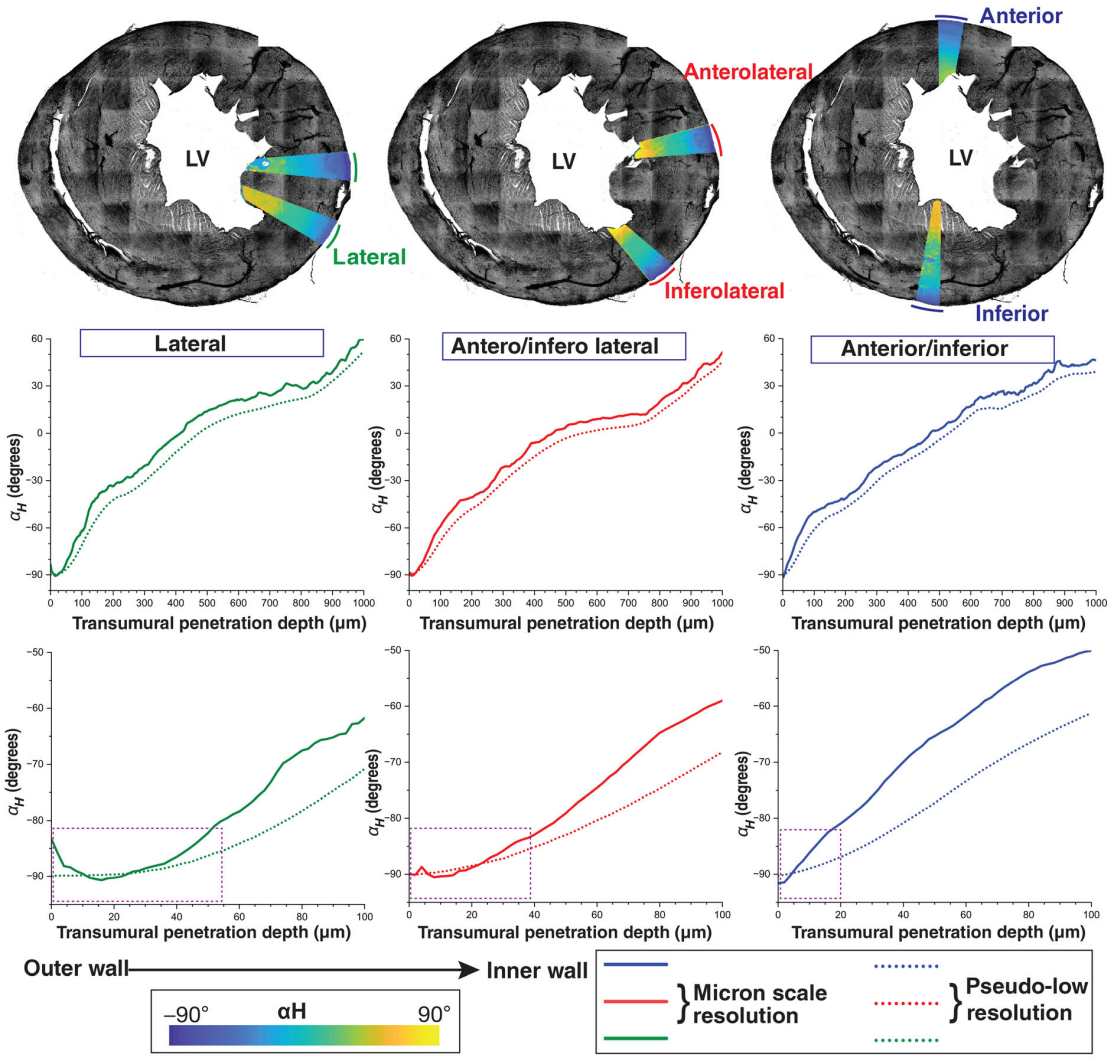
Helix angle plots for left ventricular wall sectors in a short‐axis section The individual 10° wedge‐shaped sectors are shown in three different groups; anterior/inferior, anterolateral, inferolateral, and lateral regions (top row panel). The wedge sector 3D average αH values were plotted and are highlighted as αH colormaps on a maximum intensity Z‐projection of the WGA‐stained short‐axis section in grayscale. The 3D average αH values* are plotted as a function of the transmural penetration depth walls, depicted as distance in microns along the X‐axis from the outer to the inner walls. The 3D average αH values from micron‐scale and pseudo‐resolution analyses of the selected sectors of the LV region from one representative data set of a short‐axis section (SAS3) are shown in the middle row panel. A zoomed in version of the outer‐ventricular region is provided in the bottom row panels. The region marked with a box in the bottom left panel represents the LV outer wall longitudinal cells. Data information: The values plotted here are derived from combining two sectors, spanning 10° in each, as illustrated.

In a short‐axis section, the Φ and $\alpha_{H}$ angles are related to cardiomyocyte orientation with respect to the viewing plane. $\alpha_{H}$ has been widely used to capture orientation changes along a transmural penetration from the outer to the inner heart wall (Beg *et al*, 2004; Chen *et al*, 2005; Gilbert *et al*, 2007). We therefore computed $\alpha_{H}$ at the micron scale using a ventricular outer boundary‐based estimate of the radial penetration direction at each location in the left ventricular wall (Materials and Methods; Figs 2A and E and EV2C; Movie EV4). The 3D average $\alpha_{H}$ values for several wedge‐shaped sectors were plotted as a function of transmural depth (Fig 3). The patterns of $\alpha_{H}$ values appear to be very similar at fixed angular distances with respect to the lateral region of short‐axis view of the left ventricular wall (Fig 3). Sectors in the vicinity of the lateral region reveal a sharp drop of about 20° within the first 50 μm near the circumference of the outer wall (Figs 3 and EV2D). However, the sharp change in $\alpha_{H}$ from the outer wall boundary gradually disappears further from the lateral sectors toward inferior and anterior regions (Fig 3). Immediately after this sharp decline, $\alpha_{H}$ undergoes a gradual increase of about 180° for the remainder of the left ventricular wall, in a manner that is approximately linearly proportional to transmural depth (Fig 3) although the rate of change of $\alpha_{H}$ does vary (Fig EV2D and E). This latter smooth transition of $\alpha_{H}$ through the myocardium (middle wall) until the endocardium (inner wall) is consistent with earlier findings (Beg *et al*, 2004; Chen *et al*, 2005; Gilbert *et al*, 2007; Peyrat *et al*, 2007; Lombaert *et al*, 2012; Savadjiev *et al*, 2012). However, the initial sharp transition of an approximately 20° change in *α*_*H*_, in the outer wall, is an entirely new discovery (Fig 3). We hypothesize that earlier studies lacked the required spatial resolution to identify the sharp change in $\alpha_{H}$ near the outer wall. To test this, we averaged the structure tensor, estimated at the micron scale, to a coarser spatial resolution, resulting in a pseudo low‐resolution (∼500 μm voxel resolution scale) estimate of cardiomyocyte orientation (Materials and Methods). This orientation estimate is qualitatively equivalent to the millimeter or submillimeter scale of previous DT‐MRI studies. A comparison of our micron scale and pseudo low‐resolution $\alpha_{H}$ angle estimates confirms the disappearance of the sharp change near the outermost layer, at the simulated coarser resolution (Fig 3). The long‐axis cardiomyocyte layer we have reconstructed in the outer ventricular wall might have remained obscure in past studies (Beg *et al*, 2004; Chen *et al*, 2005; Gilbert *et al*, 2007; Peyrat *et al*, 2007; Lombaert *et al*, 2012; Savadjiev *et al*, 2012) due to its narrow width of only approximately 50 μm. As such, in our simulated submillimeter‐scale coarse voxel analysis, it disappears in the orientation plots (Fig 3). In addition, a comparison of $\alpha_{H}$ between raw and preprocessed short‐axis images shows that denoising improves the $\alpha_{H}$ angle estimates (Fig EV3B). Our reconstructions also demonstrate the presence of narrow longitudinal arrangements of cardiomyocytes in the inner ventricular chamber and septum walls, which have also been described in previous studies involving histological sections (Greenbaum *et al*, 1981; Fig 2E; Movie EV3).

### Charting the continuity of the outer and inner wall long‐axis myofibers

**Figure 4:**
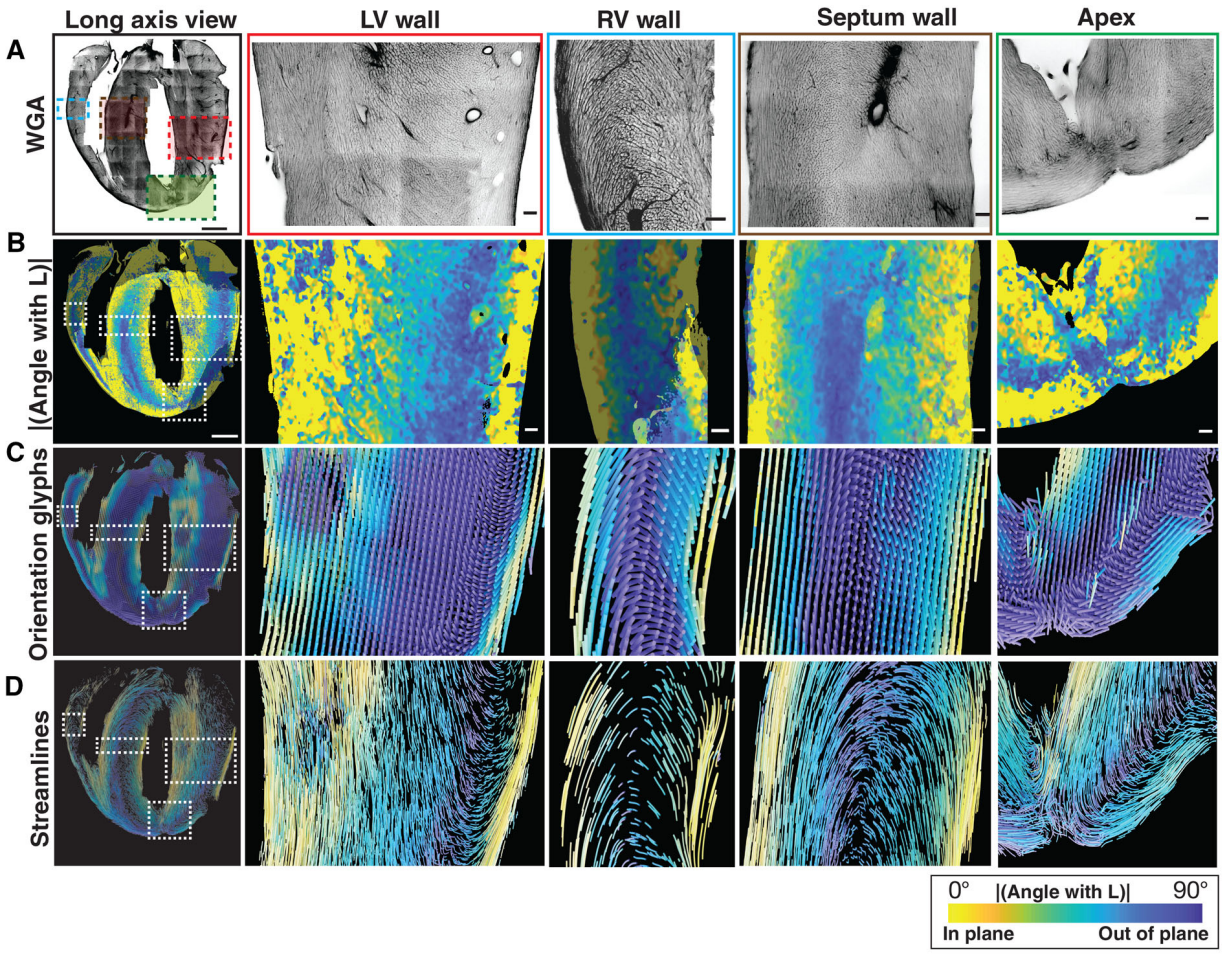
A detailed view of a long‐axis section A
WGA stain.B
Colormaps for the magnitude of the angle with the longitudinal axis (L).C
Estimated orientations as glyphs.D
Estimated orientations as streamlines. Data information: The colors follow a parula colormap, where the blue and yellow colors indicate in and out of the short‐axis plane cell orientations, respectively. Magnified views of the left ventricle (2^nd^ column), right ventricle (3rd column), septum wall (4^th^ column) and the apex region (5^th^ column) are shown for the regions in panel (A). The color bar for the angle with the longitudinal axis is as indicated. The scale bar for the complete view is 1,000 μm and for the zoomed in regions is 100 μm.

**Figure 5:**
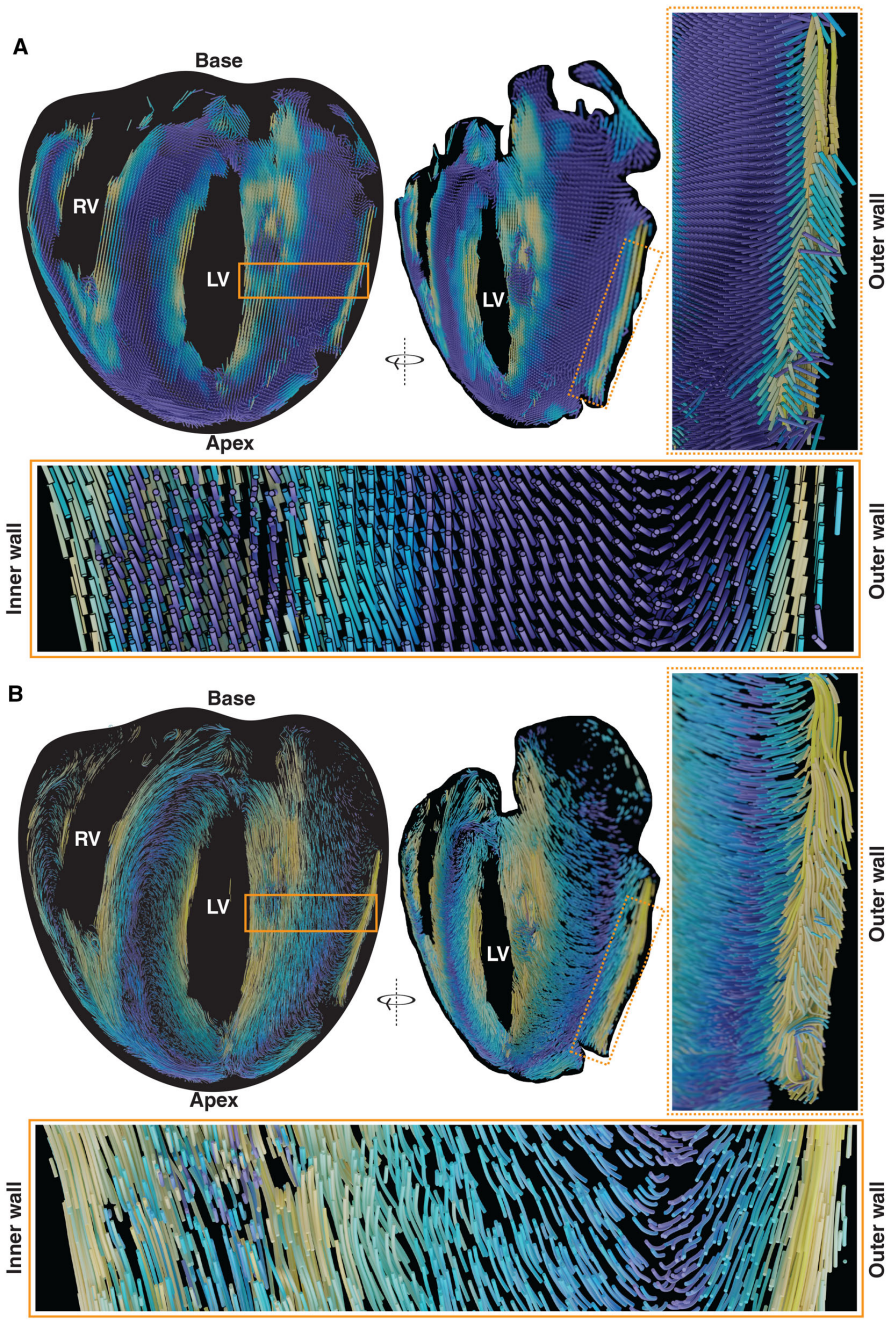
Evidence for a long‐axis fiber continuum from the analysis of a long‐axis section A, B
Estimated myofiber orientations are shown as glyphs and streamlines for the mouse LA sections as indicated. In each panel, a 3D visualization of the orientations is shown using either glyphs or streamlines, with a view obtained by rotation in a clockwise direction shown on the right (Materials and Methods). The colors follow a parula colormap, where the blue and yellow tones indicate cell orientations that are in and out of the short‐axis plane, respectively. These visualizations reveal the continuity of cell orientations across the entire long‐axis section, from base to apex.

**Figure EV5:**
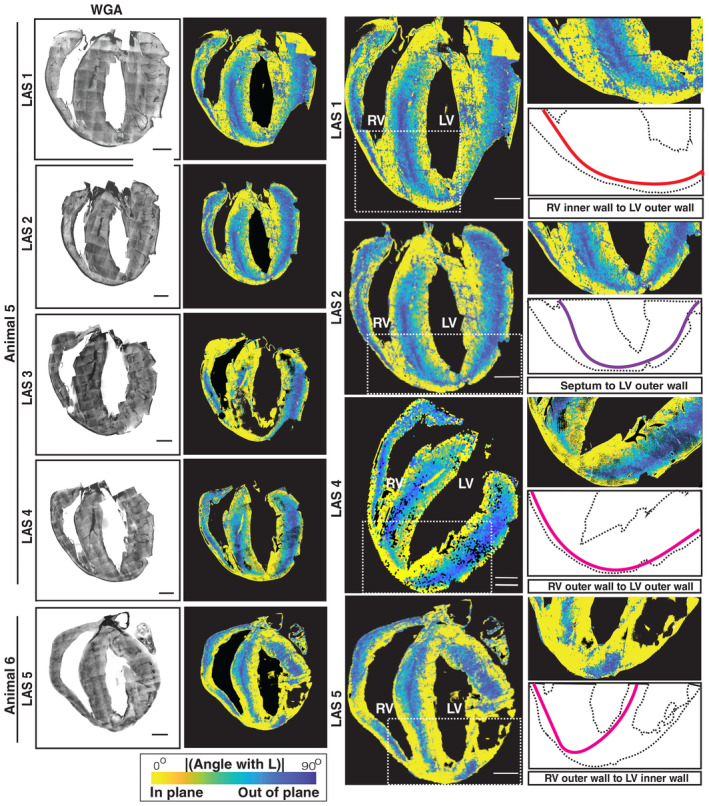
WGA staining and colormaps for the magnitude of the angle with the longitudinal axis, for different long‐axis sections and their connections at the apex Five mouse heart ventricle long‐axis sections are shown as maximum intensity Z‐projections of the WGA stain, with the magnitude of the angle between the aggregate cell orientation and longitudinal axis shown with a parula colormap marked, with a white dotted box highlighting the apex region under consideration (left column). A zoomed‐in view of the highlighted apex region from the respective long‐axis sections and illustration of long‐axis connections between different ventricular walls is also shown (right column). The continuity of the long‐axis fibers from the septum to the left ventricle outer wall, from the right ventricle inner wall to the left ventricle outer wall, from the right ventricle outer wall to the left ventricle outer wall and from the right ventricle inner wall to the left ventricle inner wall, is highlighted in the first, second, third, and fifth rows, respectively. The colorbar is as indicated, and the scale bar is 1,000 μm.

The analysis of short‐axis sections allowed us to identify narrow bands of cardiomyocytes near the boundaries of the ventricular walls that are aligned to the long‐axis direction of the heart (Fig 4A–D; Movies EV3 and EV4). To determine the spatial extent of this cardiomyocyte arrangement across the entire length of the ventricular walls, we turned our attention to the analysis of long‐axis sections (HLA‐4C). The WGA images and angular colormaps for the long‐axis sections showed that the cardiomyocytes at the edges of the ventricular wall are aligned to be parallel to the section plane (Figs 5A and B and EV5; Movie EV5). The cardiomyocyte orientations at the ventricular edges are geometrically aligned with the long‐axis direction (close to 0° with the section plane, or yellow tones in the colormaps, Figs 4 and 5 and EV5). This arrangement of cardiomyocytes extends all the way to the apex, where a complex geometry emerges. From our streamline visualizations, we infer that the bands of long‐axis myofibers extend, intertwine, and continue into the opposite ends of the ventricular walls (Figs 5 and EV5; Movies EV6 and EV7).

**Figure 6:**
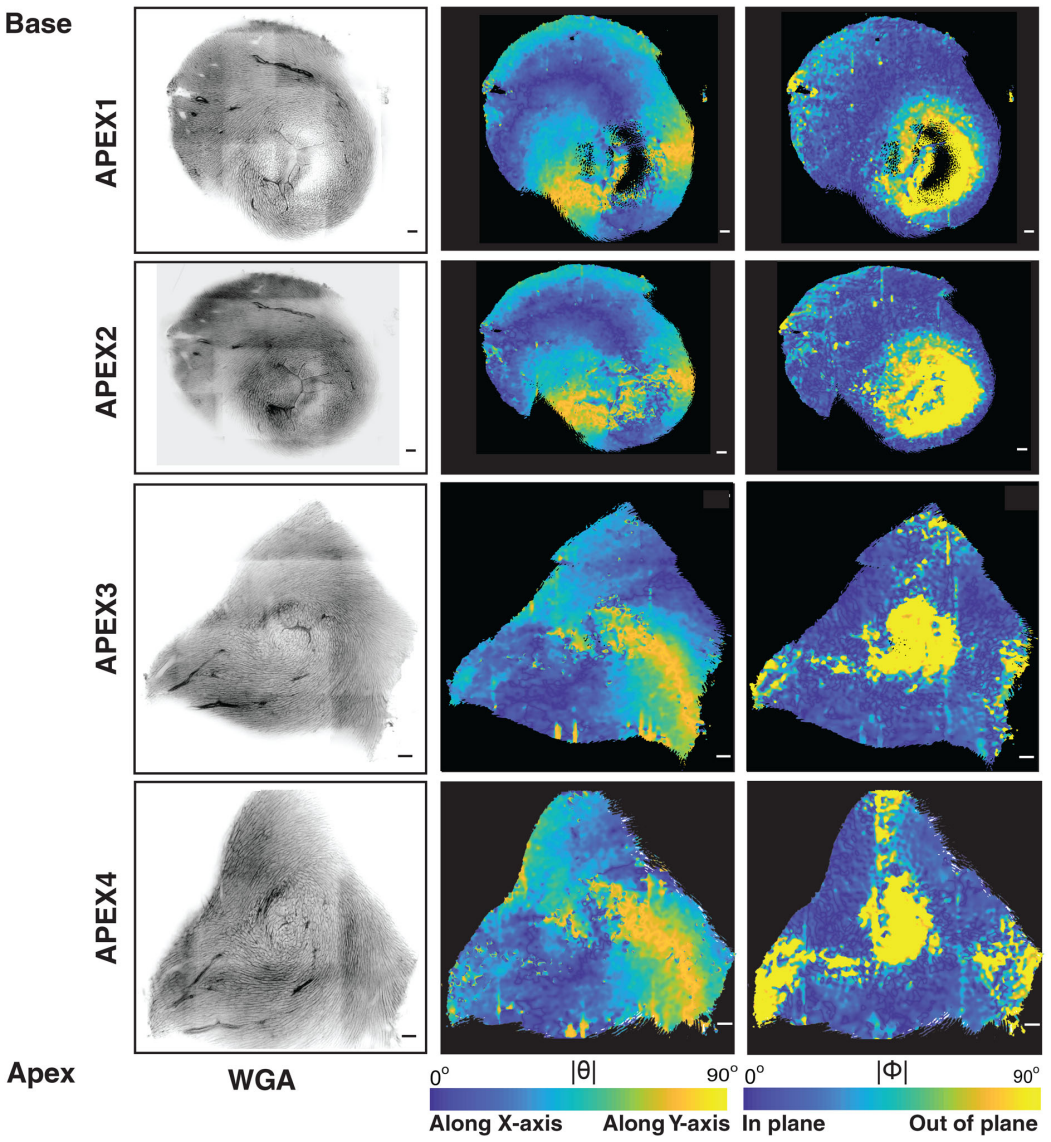
Analysis of a short‐axis section from the apex region Representative Z‐planes from four mouse heart apical serial sections are shown with WGA staining (left), and the |θ| (middle) and |Φ| (right) angles related to cell orientation using parula colormaps. The colormap scales are as indicated, with the scale bar being 100 μm.

In order to elucidate the complex geometry at the apex, we extended our sectioning toward the apex region along the short‐axis plane (Fig 6; Materials and Methods). Here, the θ angle, depicting the component of cardiomyocyte orientation in the XY plane, shows a smooth transition, similar to that in the midventricular short‐axis sections (Fig 2). The Φ angle reveals a confluence of three different bands of cardiomyocytes in the long‐axis direction (Figs 5 and EV5). At the apex, these fiber systems turn upwards toward the base of the heart and then continue to the adjacent boundaries of the heart wall (Fig 6; Movie EV8). The long‐axis cardiomyocyte arrangement in the left ventricular outer wall appears to smoothly continue into cardiomyocyte arrangements in the right ventricular outer and inner walls (Fig EV5). Thus, we observe a layer of cardiomyocytes that forms a continuum across the boundaries of the ventricular walls, running from base to apex (Appendix Fig S2A–C; Movies [Link], [Link] and EV7). This layer of cardiomyocyte arrangement when visualized using bidirectional streamlines portrays an entire long‐axis myofiber system that is orthogonal to the established circumferential myofibers (Appendix Fig S2A–C; Movies [Link], [Link], and EV7).

**Figure 7:**
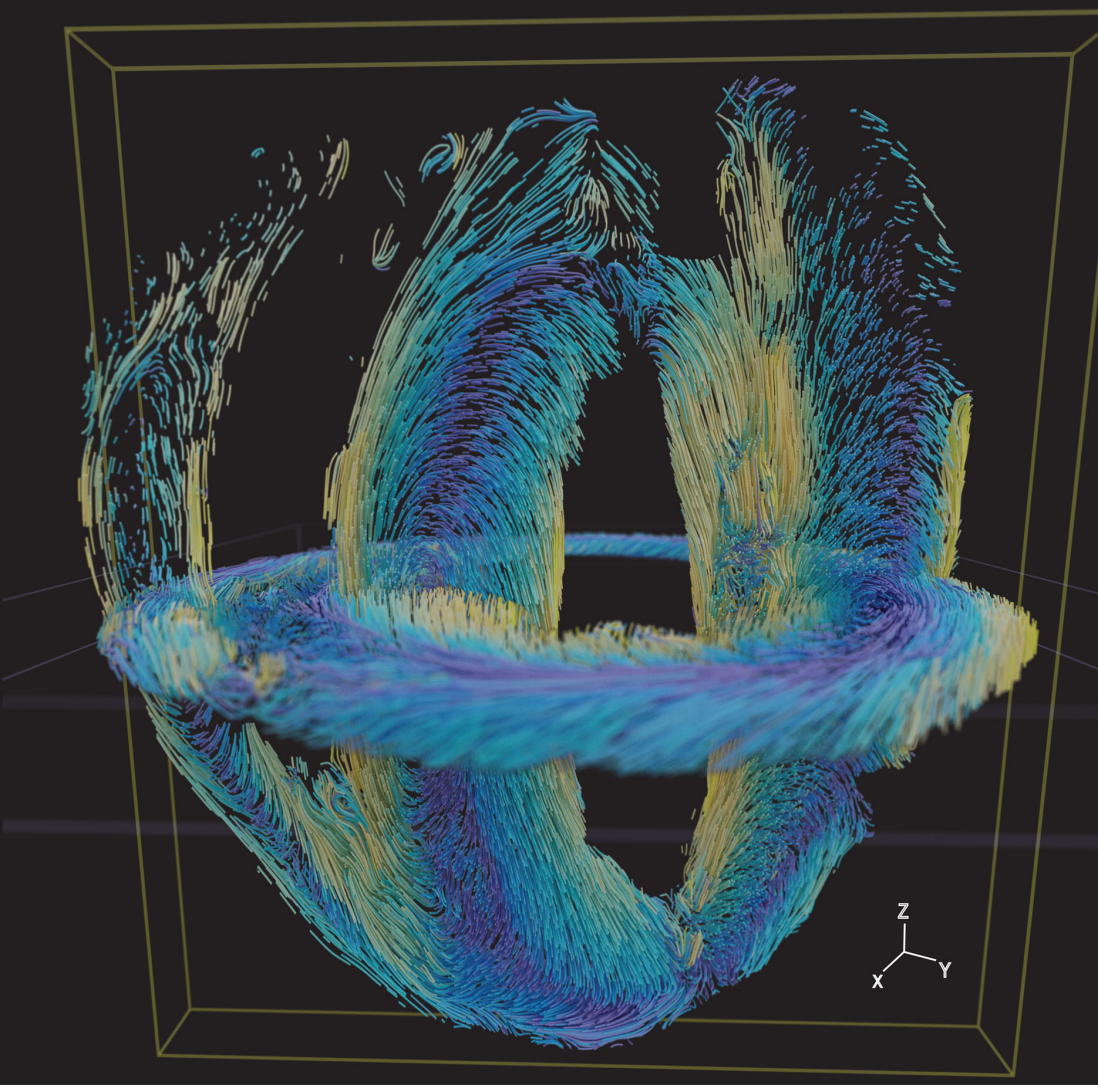
A model for orthogonal myofiber systems in heart ventricular walls The composite model is obtained by superposition of the reconstructions of the short‐axis and long‐axis sections from different mouse hearts. The structure tensor‐based orientations are visualized as streamlines (Materials and Methods). The colors follow a parula colormap, where the blue and yellow tones indicate orientations in or out of the short‐axis plane, respectively.

In summary, by combining tissue clearing, light microscopy, and computer vision, our micron‐scale recovery of cardiomyocyte orientation and myofiber geometry has revealed a long‐axis fiber system in the outer wall of the heart (Fig 7). Both the outer wall long‐axis system and the longitudinal fibers in the endocardium appear to be conserved features in the ventricular walls of four‐chambered rodent hearts (Fig EV4A–D).

## Discussion

Tissue clearing methods, together with three‐dimensional imaging of biological samples, have revealed new cell types and their organization within organs (Tomer *et al*, 2014; Morales‐Navarrete *et al*, 2019). Other studies have used tractography to visualize biological structures at various voxel resolutions including at the millimeter, submillimeter, and micron scales (Beg *et al*, 2004; Chen *et al*, 2005; Gilbert *et al*, 2007; Savadjiev *et al*, 2012; Vinegoni *et al*, 2020). However, whole organ or tissue studies have seldom combined high‐resolution three‐dimensional volumetric imaging with quantitative assessment of cell orientations using computer vision methods. Here, we have presented an integrated pipeline to recover the micron‐scale organization of densely packed cardiomyocytes in heart ventricular walls. A key advancement in our study is the integration of fluorescence‐based micron‐scale heart imaging with geometric image analysis of cardiomyocyte orientation and myofiber reconstruction methods from the microscopy images. This combination led to a resolution gain of about three orders of magnitude in scale (i.e., from millimeter to micron) for cell membrane orientation recovery, revealing a long‐axis arrangement of cardiomyocytes, a system that has eluded discovery by previous lower resolution imaging methods (Gilbert *et al*, 2007). Other high‐resolution imaging methods, such as synchrotron radiation imaging of rat hearts at 3 μm voxel resolution, have also revealed ultrastructural properties of heart tissue, including its cellular and extracellular features (Teh *et al*, 2017). However, due to a lack of cellular boundary information in such images, the geometric arrangements of cardiomyocytes cannot easily be recovered. Such methods have not reported sharp transitions in the helix angle in the anterolateral, inferolateral, and lateral regions of the LV wall. Reports of micron scale heart imaging focusing on collagen have observed a distinct epicardial pattern for the extracellular matrix (Pope *et al*, 2008), complementing our finding of long‐axis cardiomyocyte arrangements. The integrated pipeline we have described can be applied to compare alterations in cellular and subcellular structures between healthy and diseased hearts. Our approach to estimate cell orientations at the micron scale can also be used to study the arrangement of ordered cell arrays in other biological systems, such as in tissues where cells have a dominant local orientation. The application of our methodology also provides an opportunity to generate and share digitized atlases of cell orientation field data of entire organs and tissues with the research community.

The vast majority of models for heart wall contraction and electrical wave conduction have only considered a helical arrangement of cardiomyocytes, rotating smoothly in a clockwise manner in a transmural penetration from outer wall to inner wall (Vetter *et al*, 2005; Young & Panfilov, 2010; Aumentado‐Armstrong *et al*, 2018), consistent with models of fiber geometry based on DT‐MRI (Beg *et al*, 2004; Chen *et al*, 2005; Gilbert *et al*, 2007; Bayer *et al*, 2012; Savadjiev *et al*, 2012). Our reconstruction of cardiomyocyte orientations at the micron scale and descriptions of arrangements of cardiomyocytes along the long‐axis direction in the outer ventricular walls of rodent hearts could advance present understanding of heart structure and function. During heart contraction, the ventricular walls undergo a wringing motion from the apex toward the base, accompanied by a slight shortening of the heart along its long‐axis direction. Together, these motions generate an ejection force that empties the chambers. While the bulk of the contraction is carried out by the helical myofibers, which wrap around the chambers in a circumferential direction, the continuum of long‐axis fibers might also play a role in long‐axis shortening. A mathematical consideration of alternate geometries does in fact demonstrate the need for helical myofiber systems to optimize ejection fraction (Sallin, 1969). While the most recent tissue engineering papers (Chang *et al*, 2022) build on this and use a combination of helical and circular myofiber systems to simulate ventricular motion with wringing, the long‐axis fiber systems in the outer walls are still conspicuously absent. Additionally, we have observed that the three bands of outer wall long‐axis cardiomyocyte arrangements continue upwards to the base from the apex (Fig EV5).

Our reconstructions support a previous proposal, based on transmural penetrations of histological sections (Vetter *et al*, 2005), that a distinct long‐axis epicardial myofiber system exists in the right ventricle and that it plays a vital role in electrical conduction, as demonstrated by simulations of electrical wave propagation. Our 3D reconstructions at the micron scale confirm the presence of such a system, but further reveal a much more prominent system in the outer wall of the left ventricle (Figs 2, 4, and 5), which might play a complementary role in facilitating electrical conduction in the heart walls. The geometry of the outer wall long‐axis cardiomyocyte arrangements is quite complex at the apex, resembling a confluence of three systems (Fig 6). It appears to not be smooth at the apex, as a multitude of models derived from DT‐MRI have predicted. Our reconstruction of a prominent geometrically distinct long‐axis epicardial arrangement of cardiomyocytes is further supported by observations that a thin epicardial cell layer orthogonal to that in the midwall exists in humans (Drouin *et al*, 1995) and that in the rabbit and the pig (Scollan *et al*, 1998; Vetter *et al*, 2005), sharp changes of action potential coincide with changes in cell geometry and orientation in the outer wall (Yan *et al*, 1998). In a recent article using millimeter scale diffusion imaging of pig hearts, Wilson *et al* report a nonuniform variation of $\alpha_{H}$, in different regimes of the heart wall (Wilson *et al*, 2023).

The electrical signals from the sinoatrial node propagate to the atrioventricular node to cause the ventricles to pump blood, via a network of Purkinje fibers (Sedmera & Gourdie, 2014; Romero *et al*, 2016). Analyses have shown that a helical continuum of fibers, together with their associated transmural turning from the outer wall to the inner wall, can explain the point‐to‐point time to arrival conduction wave propagation maps in the heart wall, under a model of anisotropic diffusion (Young & Panfilov, 2010). Moreover, transmural turning has been shown to play a role in minimizing diffusion bias and mitigating the potentially harmful effects of stochastic propagation (Aumentado‐Armstrong *et al*, 2018). However, how the timing of the contraction is controlled at any short‐axis section is an open question. We hypothesize that the propagation of the conduction wave through the heart wall along the transmural direction could be mediated by the helical continuum of myofibers (Young & Panfilov, 2010; Aumentado‐Armstrong *et al*, 2018) and that the long‐axis outer wall fibers facilitate the propagation of the signal from the apex to the base in a longitudinal direction. This would then explain the overall timing behavior observed in the contraction of the ventricles and the need for such geometrically distinct orthogonal fiber systems.

Orthogonal fiber systems are, in fact, not restricted to mammalian hearts. Similar fiber organizations have been reported in other organs and entire animal body plans (Diamant, 1989; Mittal, 2013; Scimone *et al*, 2017). At the organ level, the coexistence of a circumferential and longitudinal fiber system is known to be important for the smooth muscle peristaltic motions seen in esophageal and intestinal tubes (Diamant, 1989; Mittal, 2013). In planarians, in addition to the circumferential and longitudinal muscle fibers, a diagonal fiber system has been reported (Scimone *et al*, 2017). However, a crucial difference between the heart and these other systems is that in the latter, the orthogonal arrays occupy an equal volume (Diamant, 1989; Mittal, 2013; Scimone *et al*, 2017). In contrast, our reconstructions show that the longitudinal cardiomyocyte arrangements account for only a small fraction of the cardiomyocytes in the heart wall, suggesting that they have highly specialized roles. It remains to be seen whether perturbations of the long‐axis arrangements are associated with heart diseases, including cardiomyopathies or electrical conduction disorders. The embryonic stage of heart development at which the outer wall long‐axis cardiomyocyte array begins to form is also not known.

Hypertrophic (HCM) and dilated (DCM) cardiomyopathies are clinically evaluated as a thickening or thinning of the left ventricular and septal walls, respectively (Marian & Braunwald, 2017). At the histological level, excess fibrosis and fiber disarray are frequently reported to be associated with these diseased states. However, a correlative evaluation of enlarged or diminished ventricular wall dimension and fiber architecture in a volumetric sense at the scale of individual cardiomyocytes is still missing. A spectrum of HCM clinical phenotypes has also been observed from echocardiogram studies among different patients (Marian & Braunwald, 2017). However, disruptions in myofiber organization caused by HCM have so far been described at a gross morphological level only using only DT‐MRI studies (Chen *et al*, 2003; Schmitt *et al*, 2009; Garcia‐Canadilla *et al*, 2019). The myofiber geometry at the micron scale reconstructed by our methods lays a foundation for analysis at a finer scale, to discriminate between symmetrical and asymmetrical fiber disruption, and more generally for the study of the association between pathological heart conditions and cardiomyocyte arrangements.

## Materials and Methods

### Experimental model and subject details

The animal preparation and image work were conducted at the NCBS/inStem Animal Care and Resource Centre and was approved by the inStem Institutional Animal Ethics Committee, following the norms specified by the Committee (Approval Numbers: INS‐IAE‐2018/03(E), INS‐IAE‐2020/12(N), NCBS‐IAE‐2012/05 (R1) SC‐5/2009 SC‐5/2012, with CPCSEA registration no. 109/GO/bc/99/CPCSEA) for control and supervision of experiments on animals (Government of India). We used the C57BL/6 strain of female mice and the Wistar strain of male rat. These were housed in the institute animal house and were maintained in a 12‐h light/dark cycle. The animals used in our studies were 6–8 weeks in age.

A flow chart illustrating the steps in the preparation and imaging of biological tissue samples is shown in Appendix Fig S3.

### Tissue clearing

The mouse and rat hearts were collected from wild‐type female C57BL/6 and Wistar strains, respectively. All the heart samples were perfused during the collection with heparinized 1× PBS to remove blood clots, followed by 4% paraformaldehyde (PFA). The fixed hearts were stored at 4°C until further use. To clear the heart tissue, we applied the CLARITY method (Tomer *et al*, 2014) with the following modifications: The fixed mouse hearts were transferred to a hydrogel monomer solution (PBS, 4% acrylamide, 4% PFA, 0.5% Bisacrylamide, and 0.25% photo‐initiator 2, 20‐Azobis[2‐(2‐imidazolin‐2‐yl) propane] dihydrochloride (VA‐044, Wako Chemicals USA) for 7 days. For initiating the hydrogel hybridization and polymerization the processed heart tissues were incubated for 3 h at 37°C. After polymerization, the excess gel material was removed. The tissue was transferred to 50 ‐ml tubes and washed 3 times with PBS, and then incubated with a clearing buffer (8% SDS and 4% boric acid in 1X PBS (pH 8.5) for 20–30 days at 37°C, in a shaking incubator at 180 rpm, with the exchange of a new clearing buffer every week. This CLARITY‐based approach applied to heart tissue samples resulted in a transparent tissue (Figs 1 and EV1A).

### Sample preparation and imaging

The cleared mouse hearts and uncleared mouse and rat hearts were subjected to short‐ and long‐axis sectioning using a Compresstome VF‐300, as illustrated in Fig EV1B. The sectioned heart tissues were processed for staining by washing them with PBS 3 times, followed by the application of PBST (PBS + 1% Triton X‐100) for 24 h at 37°C. Subsequently, the heart sections were incubated in 150 μg/ml of Alexa Fluor™ 633 conjugated wheat germ agglutinin (WGA, W21404, Thermo Fisher) for 24 h and washed with PBS, 3 times, for 10 min each time. The WGA stained cleared and uncleared heart tissues were then transferred into RIMS imaging media (88% Histodenz Sigma D2158 in 20 mM Phosphate buffer pH 7.5). The heart tissues were then mounted with fresh RIMS, sandwiched between two coverslips using 500 μm spacers (IS002, SUNjin Lab, Taiwan). The confocal images were acquired using an Olympus FV3000 microscope with an Olympus PlanApo 1.25× and Olympus UCPLFN 20X CorrM32 85 mm scale air objective (NA = 0.73). For each section, using the 1.25× objective, a lower magnification image encompassing the whole area of the section was obtained, which was then used to map the fields of view using the Olympus fluoView™ software. Micron‐scale imaging was carried out using a 20× objective, with each field of view covering 320 × 320 pixels with an isometric voxel size of 1.98 μm^3^. For the cleared and uncleared heart tissues, a maximum depth of 300 and 50 μm of image stacks was acquired, respectively. The samples were excited using a 640 nm laser line and emission was detected over a range of 650–670 nm using high sensitivity spectral detectors (gallium arsenide phosphide photo‐multiplier tube).

A flow chart illustrating the steps in the computational pipeline is shown in Appendix Fig S4.

### Preprocessing of heart tissue image stacks

At the deeper end of the image stacks, we observed a poor signal to noise ratio. In order to improve the fluorescence signal, we performed deconvolution and denoising using custom‐built algorithms. For the deconvolution, we corrected the nonideal point spread function (PSF), shown in Appendix Fig S5, using the iterative Richardson–Lucy deconvolution method (Dey *et al*, 2006) with Total variation regularization. The deconvolved image stacks were then subjected to unsupervised denoising using a dictionary learning method (Mairal *et al*, 2014). The dictionary was extracted from the shallow layers of image stacks under the assumption that the deeper and shallow layers contained common substructure elements of cells. This set of learned dictionary patches (a sparse 256 element 2D dictionary of patches of size 16 × 16, shown in Appendix Fig S6) from cardiac tissue was used to computationally clear the noise and improve the visibility of cell structures at the deeper end of the stacks. A separate dictionary file was generated for each individual field of view to prevent structures from one region from influencing the reconstruction in other regions of the heart tissue sample. The deconvolved and denoised image stacks were stitched together to yield a micron‐scale complete short‐ and long‐axis section, with a depth of about 300 μm. To achieve this, during imaging, each individual field of view was set to have at least a 25% overlap with its neighboring fields of view. The overlapping fields of view were tiled using phase correlation between adjacent fields of view in the Fourier domain (Zukić *et al*, 2021). The tiled reconstructions were globally aligned with American Heart Association (AHA) sectors in a manual fashion, using features including capillary vessels and papillary muscle placement and orientation.

### Cell orientation estimation using a structure tensor

The intensity of the WGA stain, which is absorbed by the cell membranes of the myocytes, was used to estimate myocyte orientation. At each voxel in the deconvolved and denoised image stack, we computed the structure tensor (Knutsson *et al*, 2011). We then associated to each voxel the orientation of the direction in which the intensity varied the least, capturing the long‐axis orientation of myocytes, by selecting the eigenvector of the structure tensor corresponding to the eigenvalue with smallest magnitude. The mapping of myocyte orientation to coarser spatial scales was carried out by element‐wise averaging of local structure tensors, followed by eigenvector decomposition of the smoothed tensors. To validate the structure tensor‐based estimates of cell orientations, we compared them with the ground truth orientations of several hand‐segmented myocytes. The cell orientation was then interpreted in terms of its projection in the short‐axis plane θ, the component out of the short‐axis plane Φ, and the angle *α*_*H*_ between its projection onto the heart wall tangent plane and the long‐axis direction (Fig 2A).

### Colormaps, glyphs, and streamline generation

The orientation field was represented by a 3‐dimensional array of rotated cylinders, which we refer to as “orientation glyphs.” The input vector field was approximated by a 3‐dimensional grid of equally spaced vertices, down‐sampled such that the number of vertices was not larger than 75,000. At each vertex of the down‐sampled grid, a cylinder primitive shape was created and rotated proportionally to the components of the vector field at the vertex position. A parula colormap was applied to the cylinders in a manner that was proportional to the Φ angle, using the arc cosine of the absolute value of the component of the vector field in the Z‐axis direction. Bidirectional streamlines were represented as curves extruded from polylines, whose points were computed as follows: A set of up to 25,000 points were selected from a random sample of voxels from the vector field, and each sample voxel location was the initial point for a streamline. Since the vector field represents the orientation of the tissue, each starting point initialized both positive and negative streamlines, each of which was grown by iteratively adding new points along the polyline. At each new position, the value of the field at that voxel acted to determine the position of the following point along the polyline. A parula colormap was applied to the streamlines in proportion to the Φ angle at the starting point within the vector field. To avoid artifacts where the streamlines extended beyond imaged data, a binary mask of the tissue volume with the same dimensions as the vector field served as a boundary condition.

## Author contributions

**Drisya Dileep:** Resources; data curation; software; formal analysis; validation; investigation; visualization; methodology; writing – original draft; project administration; writing – review and editing. **Tabish A Syed:** Resources; data curation; software; formal analysis; validation; investigation; visualization; methodology; writing – original draft; project administration; writing – review and editing. **Tyler FW Sloan:** Software; formal analysis; visualization; methodology. **Perundurai S Dhandapany:** Resources; funding acquisition; methodology. **Kaleem Siddiqi:** Conceptualization; supervision; funding acquisition; investigation; writing – original draft; project administration; writing – review and editing. **Minhajuddin Sirajuddin:** Conceptualization; supervision; funding acquisition; investigation; writing – original draft; project administration; writing – review and editing.

## Disclosure and competing interests statement

DD, TAS, PSD, KS, and MS declare no competing interests. TFWS is the sole proprietor of Quorumetrix Studio and provided custom scientific data processing and 3D visualization services used in this study.

## Supporting information



Appendix S1

Expanded View Figures PDF

Movie EV1

Movie EV2

Movie EV3

Movie EV4

Movie EV5

Movie EV6

Movie EV7

Movie EV8

PDF+

## Data Availability

Sample datasets and customized implementations of algorithms used for the current study are available in the GitHub repository: [https://gitfront.io/r/myoarch/fZmkh5iHEJae/myofibrometry/]. A detailed account of the methods is available as Appendix.

## References

[embj2022113288-bib-0001] Agger P , Omann C , Laustsen C , Stephenson RS , Anderson RH (2020) Anatomically correct assessment of the orientation of the cardiomyocytes using diffusion tensor imaging. NMR Biomed 33: e4205 31829484 10.1002/nbm.4205

[embj2022113288-bib-0002] Anderson RH , Smerup M , Sanchez‐Quintana D , Loukas M , Lunkenheimer PP (2009) The three‐dimensional arrangement of the myocytes in the ventricular walls. Clin Anat 22: 64–76 18567009 10.1002/ca.20645

[embj2022113288-bib-0003] Aumentado‐Armstrong T , Kadivar A , Savadjiev P , Zucker SW , Siddiqi K (2018) Conduction in the heart wall: helicoidal fibers minimize diffusion bias. Sci Rep 8: 7165 29739992 10.1038/s41598-018-25334-7 PMC5940931

[embj2022113288-bib-0004] Bayer JD , Blake RC , Plank G , Trayanova NA (2012) A novel rule‐based algorithm for assigning myocardial fiber orientation to computational heart models. Ann Biomed Eng 40: 2243–2254 22648575 10.1007/s10439-012-0593-5 PMC3518842

[embj2022113288-bib-0005] Beg MF , Helm PA , McVeigh E , Miller MI , Winslow RL (2004) Computational cardiac anatomy using MRI. Magn Reson Med 52: 1167–1174 15508155 10.1002/mrm.20255 PMC1317108

[embj2022113288-bib-0006] Cerqueira MD , Weissman NJ , Dilsizian V , Jacobs AK , Kaul S , Laskey WK , Pennell DJ , Rumberger JA , Ryan T , Verani MS *et al* (2002) Standardized myocardial segmentation and nomenclature for tomographic imaging of the heart. A statement for healthcare professionals from the Cardiac Imaging Committee of the Council on Clinical Cardiology of the American Heart Association. Circulation 105: 539–542 11815441 10.1161/hc0402.102975

[embj2022113288-bib-0007] Chang H , Liu Q , Zimmerman JF , Lee KY , Jin Q , Peters MM , Rosnach M , Choi S , Kim SL , Ardoña HAM *et al* (2022) Recreating the heart's helical structure‐function relationship with focused rotary jet spinning. Science 377: 180–185 35857545 10.1126/science.abl6395 PMC10077766

[embj2022113288-bib-0008] Chen J , Song S‐K , Liu W , McLean M , Allen JS , Tan J , Wickline SA , Yu X (2003) Remodeling of cardiac fiber structure after infarction in rats quantified with diffusion tensor MRI. Am J Physiol Heart Circ Physiol 285: H946–H954 12763752 10.1152/ajpheart.00889.2002

[embj2022113288-bib-0009] Chen J , Liu W , Zhang H , Lacy L , Yang X , Song S‐K , Wickline SA , Yu X (2005) Regional ventricular wall thickening reflects changes in cardiac fiber and sheet structure during contraction: quantification with diffusion tensor MRI. Am J Physiol Heart Circ Physiol 289: H1898–H1907 16219812 10.1152/ajpheart.00041.2005

[embj2022113288-bib-0010] Corno AF , Kocica MJ , Torrent‐Guasp F (2006) The helical ventricular myocardial band of Torrent‐Guasp: potential implications in congenital heart defects. Eur J Cardiothorac Surg 29: S61–S68 16567102 10.1016/j.ejcts.2006.02.049

[embj2022113288-bib-0011] Cretoiu D , Pavelescu L , Duica F , Radu M , Suciu N , Cretoiu SM (2018) Myofibers. In Muscle atrophy, Xiao J (ed), pp 23–46. Singapore: Springer 10.1007/978-981-13-1435-3_2 30390246

[embj2022113288-bib-0012] von Deuster C , Sammut E , Asner L , Nordsletten D , Lamata P , Stoeck CT , Kozerke S , Razavi R (2016) Studying dynamic myofiber aggregate reorientation in dilated cardiomyopathy using in vivo magnetic resonance diffusion tensor imaging. Circ Cardiovasc Imaging 9: e005018 27729361 10.1161/CIRCIMAGING.116.005018 PMC5068188

[embj2022113288-bib-0013] Dey N , Blanc‐Feraud L , Zimmer C , Roux P , Kam Z , Olivo‐Marin J‐C , Zerubia J (2006) Richardson–Lucy algorithm with total variation regularization for 3D confocal microscope deconvolution. Microsc Res Tech 69: 260–266 16586486 10.1002/jemt.20294

[embj2022113288-bib-0014] Diamant NE (1989) Physiology of esophageal motor function. Gastroenterol Clin North Am 18: 179–194 2668166

[embj2022113288-bib-0015] Drouin E , Charpentier F , Gauthier C , Laurent K , Le Marec H (1995) Electrophysiologic characteristics of cells spanning the left ventricular wall of human heart: evidence for presence of M cells. J Am Coll Cardiol 26: 185–192 7797750 10.1016/0735-1097(95)00167-x

[embj2022113288-bib-0016] Garcia‐Canadilla P , Cook AC , Mohun TJ , Oji O , Schlossarek S , Carrier L , McKenna WJ , Moon JC , Captur G (2019) Myoarchitectural disarray of hypertrophic cardiomyopathy begins pre‐birth. J Anat 235: 962–976 31347708 10.1111/joa.13058 PMC6794206

[embj2022113288-bib-0017] Geerts‐Ossevoort L , Bovendeerd P , Prinzen F , Arts T , Nicolay K (2001) Myofiber orientation in the normal and infarcted heart, assessed with MR‐diffusion tensor imaging. Comput Cardiol 28: 621–624

[embj2022113288-bib-0018] Gilbert SH , Benson AP , Li P , Holden AV (2007) Regional localisation of left ventricular sheet structure: integration with current models of cardiac fibre, sheet and band structure. Eur J Cardiothorac Surg 32: 231–249 17462906 10.1016/j.ejcts.2007.03.032

[embj2022113288-bib-0019] Granlund GH , Knutsson H (1994) Signal processing for computer vision. Berlin, Germany: Springer Science & Business Media

[embj2022113288-bib-0020] Greenbaum RA , Ho SY , Gibson DG , Becker AE , Anderson RH (1981) Left ventricular fibre architecture in man. Br Heart J 45: 248–263 7008815 10.1136/hrt.45.3.248 PMC482521

[embj2022113288-bib-0021] Helm P , Beg MF , Miller MI , Winslow RL (2005) Measuring and mapping cardiac fiber and laminar architecture using diffusion tensor MR imaging. Ann N Y Acad Sci 1047: 296–307 16093505 10.1196/annals.1341.026

[embj2022113288-bib-0022] Horowitz A , Perl M , Sideman S (1993) Geodesics as a mechanically optimal fiber geometry for the left ventricle. Basic Res Cardiol 88: 67–74 8147836

[embj2022113288-bib-0023] Jouk PS , Usson Y , Michalowicz G , Grossi L (2000) Three‐dimensional cartography of the pattern of the myofibres in the second trimester fetal human heart. Anat Embryol (Berl) 202: 103–118 10985430 10.1007/s004290000103

[embj2022113288-bib-0024] Knutsson H , Westin C‐F , Andersson M (2011) Representing local structure using tensors II. In Image analysis, Heyden A , Kahl F (eds), pp 545–556. Berlin, Heidelberg: Springer

[embj2022113288-bib-0025] Leemans A (2010) Visualization of diffusion MRI data. In Diffusion MRI: theory, methods, and applications, Jones DK (ed), pp 354–380. Oxford, UK: Oxford University Press

[embj2022113288-bib-0026] LeGrice IJ , Smaill BH , Chai LZ , Edgar SG , Gavin JB , Hunter PJ (1995) Laminar structure of the heart: ventricular myocyte arrangement and connective tissue architecture in the dog. Am J Physiol 269: H571–H582 7653621 10.1152/ajpheart.1995.269.2.H571

[embj2022113288-bib-0027] Libby P , Bonow R , Mann D , Tomaselli G , Bhatt D , Solomon S , Braunwald E (2018) Braunwald's heart disease: a textbook of cardiovascular medicine, 2‐volume set 11^th^ edition. Philadelphia, PA: Elsevier Science

[embj2022113288-bib-0028] Lombaert H , Peyrat J‐M , Croisille P , Rapacchi S , Fanton L , Cheriet F , Clarysse P , Magnin I , Delingette H , Ayache N (2012) Human atlas of the cardiac fiber architecture: study on a healthy population. IEEE Transactions on Medical Imaging 31: 1436–1447 22481815 10.1109/TMI.2012.2192743

[embj2022113288-bib-0029] Mairal J , Bach F , Ponce J (2014) Sparse modeling for image and vision processing. Hanover, MA: Now Publishers Inc.

[embj2022113288-bib-0030] Marian AJ , Braunwald E (2017) Hypertrophic Cardiomyopathy. Circ Res 121: 749–770 28912181 10.1161/CIRCRESAHA.117.311059 PMC5654557

[embj2022113288-bib-0031] Merz SF , Korste S , Bornemann L , Michel L , Stock P , Squire A , Soun C , Engel DR , Detzer J , Lörchner H *et al* (2019) Contemporaneous 3D characterization of acute and chronic myocardial I/R injury and response. Nat Commun 10: 2312 31127113 10.1038/s41467-019-10338-2 PMC6534576

[embj2022113288-bib-0032] Mittal RK (2013) Longitudinal muscle of the esophagus: its role in esophageal health and disease. Curr Opin Gastroenterol 29: 421–430 23739655 10.1097/MOG.0b013e3283622b57

[embj2022113288-bib-0033] Morales‐Navarrete H , Nonaka H , Scholich A , Segovia‐Miranda F , de Back W , Meyer K , Bogorad RL , Koteliansky V , Brusch L , Kalaidzidis Y *et al* (2019) Liquid‐crystal organization of liver tissue. eLife 8: e44860 31204997 10.7554/eLife.44860 PMC6598764

[embj2022113288-bib-0034] Nehrhoff I , Ripoll J , Samaniego R , Desco M , Gómez‐Gaviro MV (2017) Looking inside the heart: a see‐through view of the vascular tree. Biomed Opt Express 8: 3110–3118 28663930 10.1364/BOE.8.003110 PMC5480453

[embj2022113288-bib-0035] Perbellini F , Liu AKL , Watson SA , Bardi I , Rothery SM , Terracciano CM (2017) Free‐of‐Acrylamide SDS‐based Tissue Clearing (FASTClear) for three dimensional visualization of myocardial tissue. Sci Rep 7: 5188 28701763 10.1038/s41598-017-05406-w PMC5507863

[embj2022113288-bib-0036] Peskin CS (1989) Fiber architecture of the left ventricular wall: an asymptotic analysis. Commun Pure Appl Math 42: 79–113

[embj2022113288-bib-0037] Peyrat J‐M , Sermesant M , Pennec X , Delingette H , Xu C , McVeigh ER , Ayache N (2007) A computational framework for the statistical analysis of cardiac diffusion tensors: application to a small database of canine hearts. IEEE Trans Med Imaging 26: 1500–1514 18041265 10.1109/TMI.2007.907286

[embj2022113288-bib-0038] Pope AJ , Sands GB , Smaill BH , LeGrice IJ (2008) Three‐dimensional transmural organization of perimysial collagen in the heart. Am J Physiol Heart Circ Physiol 295: H1243–H1252 18641274 10.1152/ajpheart.00484.2008 PMC2544485

[embj2022113288-bib-0039] Poveda F , Gil D , Martí E , Andaluz A , Ballester M , Carreras F (2013) Helical structure of the cardiac ventricular anatomy assessed by diffusion tensor magnetic resonance imaging with multiresolution tractography. Rev Esp Cardiol (Engl Ed) 66: 782–790 24773858 10.1016/j.rec.2013.04.021

[embj2022113288-bib-0040] Rohmer D , Sitek A , Gullberg GT (2007) Reconstruction and visualization of fiber and laminar structure in the normal human heart from ex vivo diffusion tensor magnetic resonance imaging (DTMRI) data. Invest Radiol 42: 777–789 18030201 10.1097/RLI.0b013e3181238330

[embj2022113288-bib-0041] Romero D , Camara O , Sachse F , Sebastian R (2016) Analysis of microstructure of the cardiac conduction system based on three‐dimensional confocal microscopy. PLoS One 11: e0164093 27716829 10.1371/journal.pone.0164093 PMC5055359

[embj2022113288-bib-0042] Rushmer RF , Crystal DK , Wagner C (1953) The functional anatomy of ventricular contraction. Circ Res 1: 162–170 13042943 10.1161/01.res.1.2.162

[embj2022113288-bib-0043] Sallin EA (1969) Fiber orientation and ejection fraction in the human left ventricle. Biophys J 9: 954–964 5791550 10.1016/S0006-3495(69)86429-5 PMC1367490

[embj2022113288-bib-0044] Savadjiev P , Strijkers GJ , Bakermans AJ , Piuze E , Zucker SW , Siddiqi K (2012) Heart wall myofibers are arranged in minimal surfaces to optimize organ function. Proc Natl Acad Sci USA 109: 9248–9253 22645368 10.1073/pnas.1120785109 PMC3386057

[embj2022113288-bib-0045] Schmitt B , Fedarava K , Falkenberg J , Rothaus K , Bodhey NK , Reischauer C , Kozerke S , Schnackenburg B , Westermann D , Lunkenheimer PP *et al* (2009) Three‐dimensional alignment of the aggregated myocytes in the normal and hypertrophic murine heart. J Appl Physiol 107: 921–927 19628727 10.1152/japplphysiol.00275.2009

[embj2022113288-bib-0046] Scimone ML , Cote LE , Reddien PW (2017) Orthogonal muscle fibres have different instructive roles in planarian regeneration. Nature 551: 623–628 29168507 10.1038/nature24660 PMC6263039

[embj2022113288-bib-0047] Scollan DF , Holmes A , Winslow R , Forder J (1998) Histological validation of myocardial microstructure obtained from diffusion tensor magnetic resonance imaging. Am J Physiol 275: H2308–H2318 9843833 10.1152/ajpheart.1998.275.6.H2308

[embj2022113288-bib-0048] Sedmera D , Gourdie RG (2014) Why do we have Purkinje fibers deep in our heart? Physiol Res 63: S9–S18 24564668 10.33549/physiolres.932686

[embj2022113288-bib-0049] Seidel T , Edelmann J‐C , Sachse FB (2016) Analyzing remodeling of cardiac tissue: a comprehensive approach based on confocal microscopy and 3D reconstructions. Ann Biomed Eng 44: 1436–1448 26399990 10.1007/s10439-015-1465-6 PMC4805509

[embj2022113288-bib-0050] Sivaguru M , Fried G , Sivaguru BS , Sivaguru VA , Lu X , Choi KH , Saif MTA , Lin B , Sadayappan S (2015) Cardiac muscle organization revealed in 3‐D by imaging whole‐mount mouse hearts using two‐photon fluorescence and confocal microscopy. Biotechniques 59: 295–308 26554507 10.2144/000114356

[embj2022113288-bib-0051] Spotnitz HM (2000) Macro design, structure, and mechanics of the left ventricle. J Thorac Cardiovasc Surg 119: 1053–1077 10788831 10.1016/S0022-5223(00)70106-1

[embj2022113288-bib-0052] Streeter DD , Spotnitz HM , Patel DP , John R , Sonnenblick EH (1969) Fiber orientation in the canine left ventricle during diastole and systole. Circ Res 24: 339–347 5766515 10.1161/01.res.24.3.339

[embj2022113288-bib-0053] Teh I , McClymont D , Zdora M‐C , Whittington HJ , Davidoiu V , Lee J , Lygate CA , Rau C , Zanette I , Schneider JE (2017) Validation of diffusion tensor MRI measurements of cardiac microstructure with structure tensor synchrotron radiation imaging. J Cardiovasc Magn Reson 19: 31 28279178 10.1186/s12968-017-0342-x PMC5345150

[embj2022113288-bib-0054] Tomer R , Ye L , Hsueh B , Deisseroth K (2014) Advanced CLARITY for rapid and high‐resolution imaging of intact tissues. Nat Protoc 9: 1682–1697 24945384 10.1038/nprot.2014.123 PMC4096681

[embj2022113288-bib-0055] Vetter FJ , Simons SB , Mironov S , Hyatt CJ , Pertsov AM (2005) Epicardial fiber organization in swine right ventricle and its impact on propagation. Circ Res 96: 244–251 15618536 10.1161/01.RES.0000153979.71859.e7

[embj2022113288-bib-0056] Vinegoni C , Fumene Feruglio P , Courties G , Schmidt S , Hulsmans M , Lee S , Wang R , Sosnovik D , Nahrendorf M , Weissleder R (2020) Fluorescence microscopy tensor imaging representations for large‐scale dataset analysis. Sci Rep 10: 5632 32221334 10.1038/s41598-020-62233-2 PMC7101442

[embj2022113288-bib-0057] Wilson AJ , Han QJ , Perotti LE , Ennis DB (2023) Ventricular helix angle trends and long‐range connectivity. In Functional imaging and modeling of the heart, Bernard O , Clarysse P , Duchateau N , Ohayon J , Viallon M (eds), pp 64–73. Cham: Springer Nature Switzerland

[embj2022113288-bib-0058] Yan GX , Shimizu W , Antzelevitch C (1998) Characteristics and distribution of M cells in arterially perfused canine left ventricular wedge preparations. Circulation 98: 1921–1927 9799214 10.1161/01.cir.98.18.1921

[embj2022113288-bib-0059] Young RJ , Panfilov AV (2010) Anisotropy of wave propagation in the heart can be modeled by a Riemannian electrophysiological metric. Proc Natl Acad Sci USA 107: 15063–15068 20696934 10.1073/pnas.1008837107 PMC2930580

[embj2022113288-bib-0060] Young AA , Legrice IJ , Young MA , Smaill BH (1998) Extended confocal microscopy of myocardial laminae and collagen network. J Microsc 192: 139–150 9853371 10.1046/j.1365-2818.1998.00414.x

[embj2022113288-bib-0061] Zukić D , Jackson M , Dimiduk D , Donegan S , Groeber M , McCormick M (2021) ITKMontage: a software module for image stitching. Integr Mater Manuf Innov 10: 115–124

