# Supplementary material for: Cardiomyocyte orientation recovery at micrometer scale reveals long‐axis fiber continuum in heart walls
Source: EMBO J. 2023 Sep 6;42(19):e113288. doi: 10.15252/embj.2022113288 (PMC10548172; doi:10.15252/embj.2022113288)
Supplement: Supplementary file 1 — Appendix S1 [file EMBJ-42-e113288-s003.pdf]

Cardiomyocyte orientation recovery at micron scale reveals long-axis fiber continuum in heart walls

Drisya Dileep[*,†] Tabish A. Syed[*,‡] Tyler F. W. Sloan[§]
Perundurai S. Dhandapany[†] Kaleem Siddiqi[‡] Minhajuddin Sirajuddin[†]

Appendix Table of Contents

Appendix Figure S1 2

Appendix Figure S2 3

1 Introduction to Methods 4

2 Biological Methods 5

2.1 Experimental procedures 5
2.2 Tissue preparation 6
2.3 Alignment of different short-axis sections 8
2.4 Analysis of short-axis sections from uncleared mouse and rat hearts 8

3 Computer Vision Methods 9

3.1 Deconvolution of acquired data 9
3.2 Denoising 9
3.3 Stitching 3D blocks 10
3.4 Orientation field estimation 11
3.5 Computation of the Helix Angle 11
3.6 Smoothing the Estimated Orientation Field 11
3.7 Validating Orientation Estimates 12
3.8 Φ, θ and α_H calculation and colormap generation 13
3.9 Computing the transmural rate of change of α_H 13
3.10 3D Rendered Visualizations and Animations 13

Appendix Table S1 15

*Equal Contribution
†Institute for Stem Cell Science and Regenerative Medicine, Bangalore, India.
‡School of Computer Science and Centre for Intelligent Machines, McGill University, and MILA - Québec AI Institute, Montréal, Canada.
§Quorumetrix Studio, Montréal

Appendix Figure S1

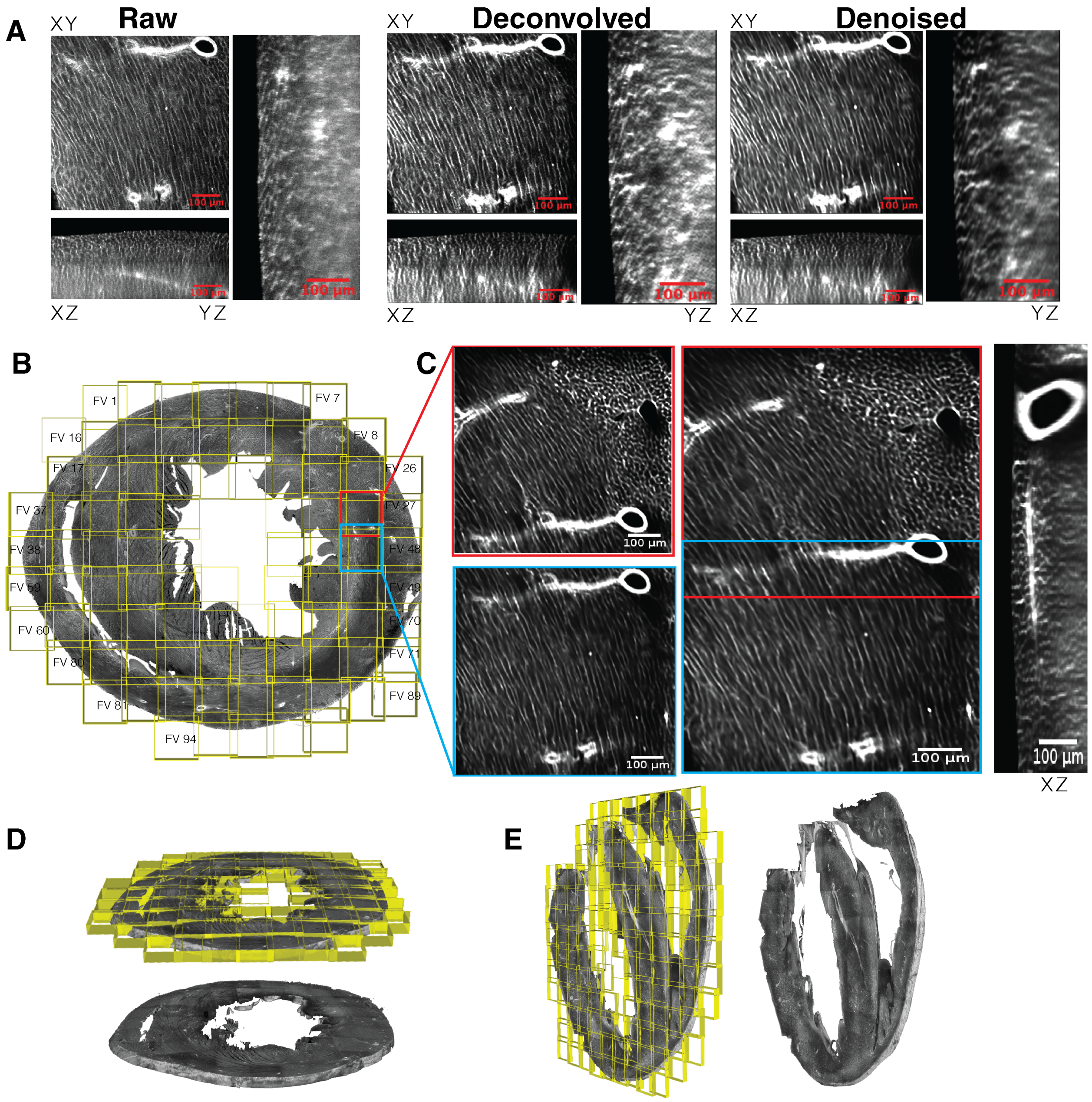

Appendix Figure S1: Preprocessing and stitching individual fields of view.

A. A representative field of view (left), followed by deconvolution (middle) and denoising (right). The orthogonal views (XZ and YZ) show an improvement in the signal to noise ratio towards the deeper Z-sections. The scale bar is 100 microns.

B. Individual fields of view (FVs) overlayed as a grid on the reconstructed full short-axis section. The individual FVs were imaged using a snake pattern, row by row. Each FV is 320 microns2 in dimension and has a 25% overlap with its neighboring FVs.

C. Left panel; An example showing the stitching of two neighboring FVs (FV28 and FV47, shown in red and blue boxes, respectively). Middle panel; A zoomed in view of the stitched result, showing the alignment of features in the common region. Right panel; An XZ/YZ view of the common region. The scale bar is as indicated.

D-E. 3D-views of the stitched short-axis and long-axis sections, with the individual FVs shown in yellow in an overlayed grid pattern.

Appendix Figure S2

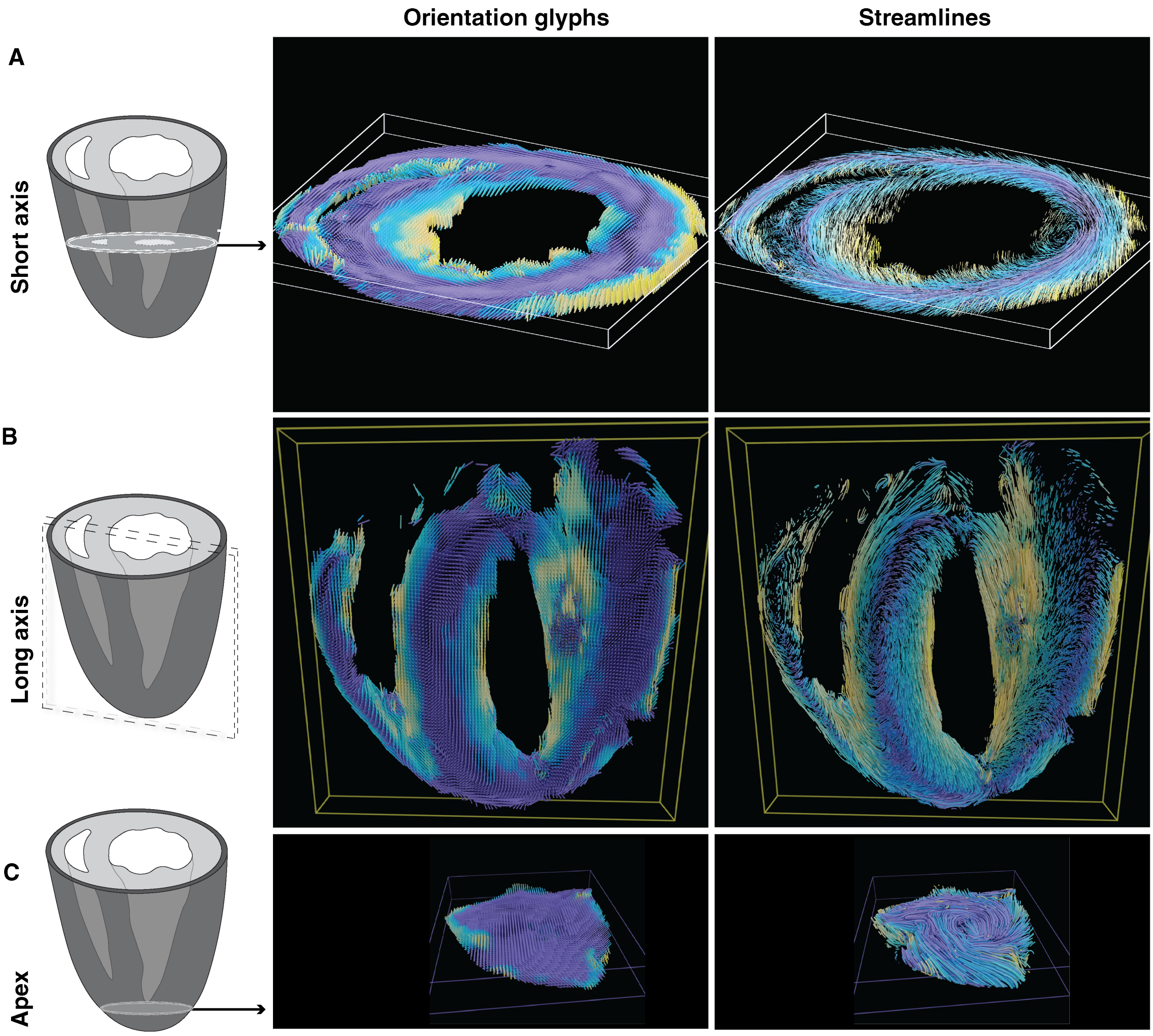

Appendix Figure S2: Long-axis and circumferential myofibers in the heart ventricular walls.

A. A short-axis (mid-ventricular region) section.

B. A long-axis (transverse section).

C. An apical section.

Data information: Schematics of the heart ventricle walls and the heart sections analyzed (left). Structure tensor based orientations are visualized as glyphs (middle) and streamlines (right) (Methods). The colors follow a parula colormap, where the blue and yellow tones indicate orientations that are in or out of the short-axis plane, respectively.

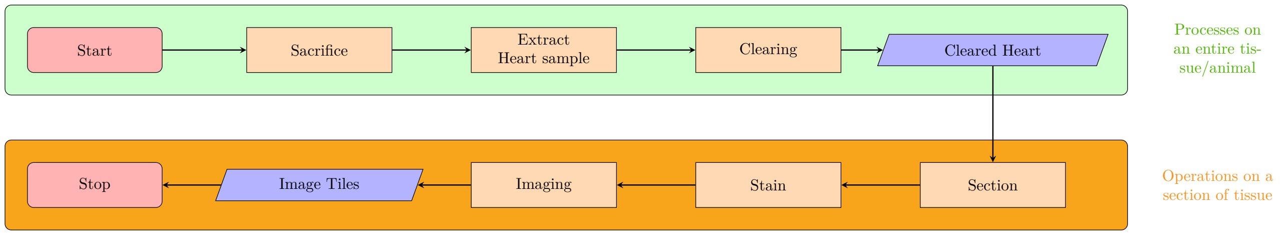

Appendix Figure S3: A flowchart of the steps in biological tissue preparation. The top row depicts operations performed on the entire tissue (or animal) while the bottom row depicts operations performed on sections of tissue.

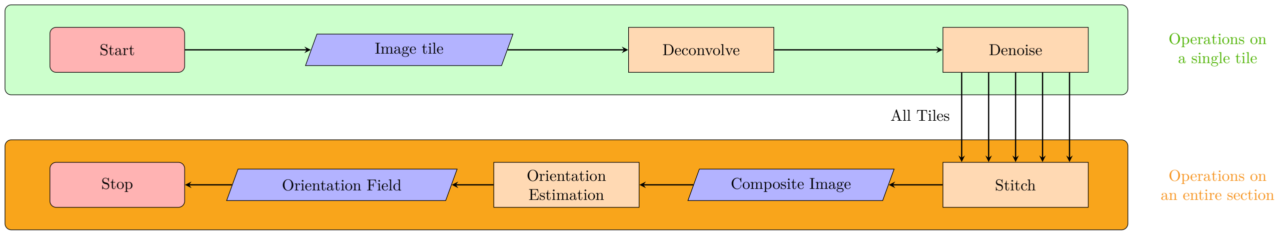

Appendix Figure S4: A flowchart illustrating the computational pipeline, starting from input image tiles, to generate a composite tiled image and a dense myocyte orientation field. The top row shows operations performed on individual image tiles while the bottom row shows operations performed on the entire stitched composite image.

1 Introduction to Methods

Here we present a detailed account of the steps used to recover myofiber organization in the mouse heart. All experiments were performed on wild type female mice of the C57BL/6 strain. All the experimental animals used in this study were maintained in the NCBS/inStem Animal Care and Resource facility in well ventilated cages with 12-hour light/12-hour dark cycles. The heart samples were collected from mice aged between 6 and 8 weeks. In Section 2 we describe the biological tissue preparation methods up to the imaging of the tissue samples using a confocal microscope. The flowchart in Fig. **S3** outlines the steps used for preparing heart samples and then tissue sections for imaging. In Section 3 we describe the computational methods used to estimate the cellular orientation of myocytes and to then reconstruct streamlines to represent the orientations of myofibers in entire heart sections. The flowchart in Fig. **S4** depicts the computational steps for generating a field representing myocyte orientations, starting from 3D images of small tiles of heart tissue sections.

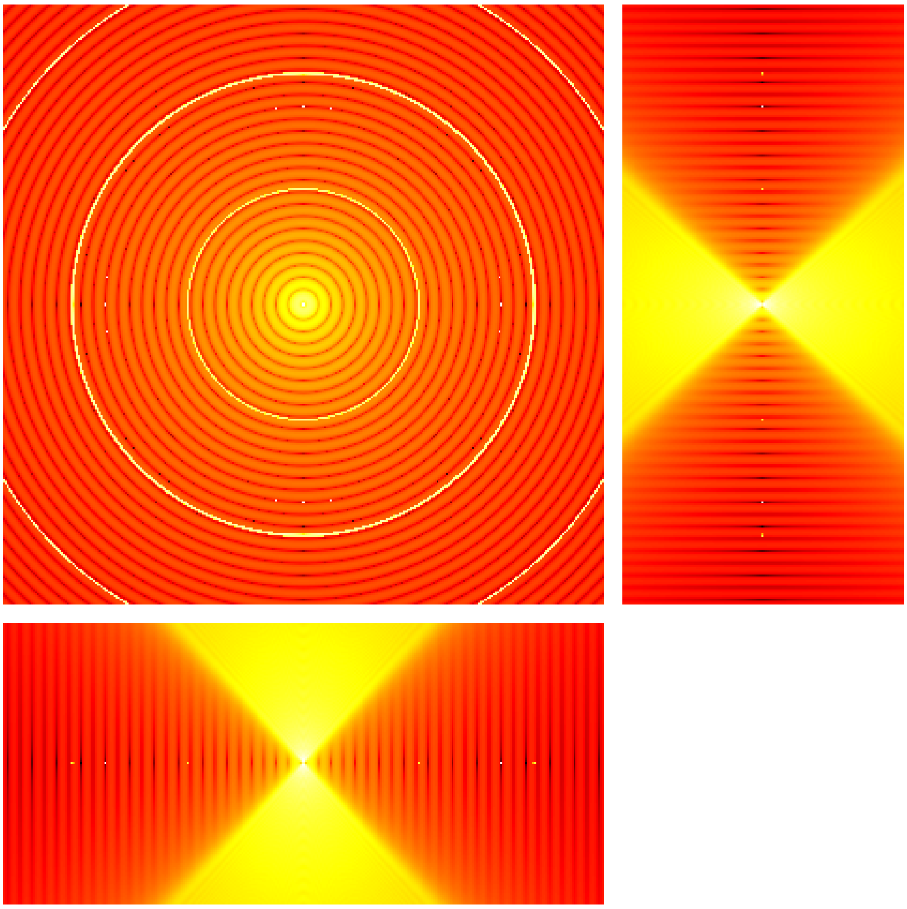

Appendix Figure S5: A visualization of the middle planes of a 3D point spread function (PSF) along the XY (top left), XZ (bottom left) and YZ (top right) directions, shown in log intensity scale (increasing from black through yellow and red to white).

2 Biological Methods

2.1 Experimental procedures

CLARITY based clearing protocol applied to the Mouse Heart: Each animal was sacrificed and its heart was gently accessed by cutting the abdominal cavity. The heart was then immediately perfused to remove blood and clots from the tissue. A small incision was made in the right atrium to facilitate fast perfusion of the heart chambers. The perfusion was carried out manually using a 26 Gauge syringe needle, inserted at an inclined angle, in the apex region of the right ventricle. We injected 10X phosphate-buffered saline solution (PBS) with a stock solution containing 1.37 M NaCl, 27 mM KCl, 100 mM Na_2HPO_4, and 18 mM KH_2PO_4, with the pH adjusted to 7.4. Initially, ice cold heparinized 1X PBS was passed through the syringe, followed by ice cold 4% paraformaldehyde (PFA). Subsequently, a hydrogel monomer solution consisting of 4% acrylamide, 4% PFA, 0.5% Bisacrylamide and 0.25% photo-initiator 2, 20-Azobis[2-(2-imidazolin-2-yl)propane] dihydrochloride (VA-044, Wako Chemicals USA) in PBS was perfused through the heart, as described previously for CLARITY based clearing of brain tissue [1]. The fixed mouse heart sample was transferred into a 50ml tube and incubated at 4 °C for seven days in the hydrogel monomer solution. The fixed heart

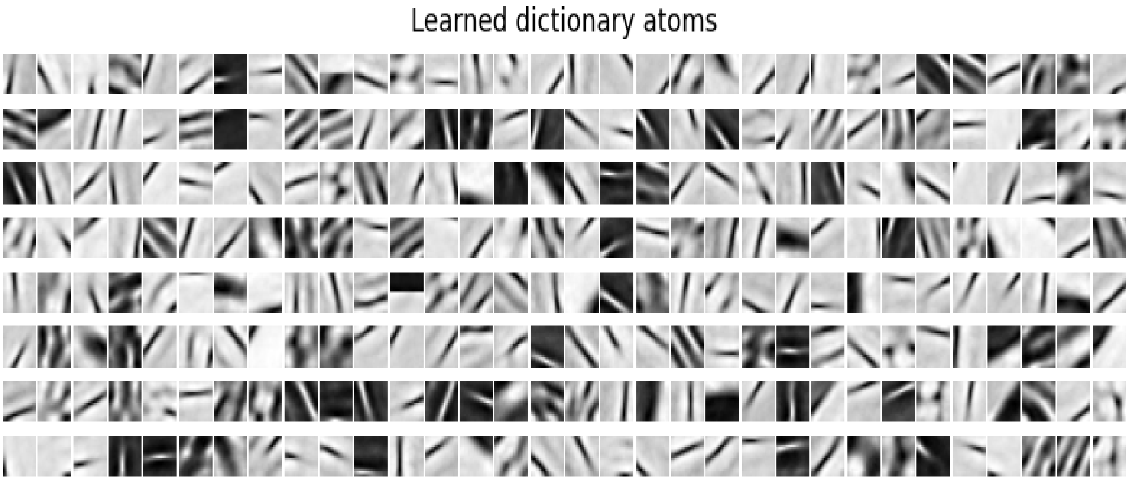

Appendix Figure S6: A visualization of the dictionary atoms learned for the mouse cardiac tissue microscopy image dataset SAS1.

tissues were then degassed for 10 minutes using a vacuum chamber at room temperature. To initiate tissue-hydrogel hybridization and polymerization, the processed heart tissues were then incubated for 3 hours at 37 °C. After polymerization, excess gel material was carefully removed by gently rubbing the tissue with soft tissue wipes. The tissue was transferred to 50 ml tubes for 1X PBS washes, which were carried out 3 times for a duration of 10 minutes each time. The tissue was further incubated with a clearing buffer (8% SDS and 4% boric acid in 1X PBS (pH 8.5)) for 20-30 days at 37 °C, in a shaking incubator (180 rpm) with a buffer exchange occurring every week. This CLARITY based approach applied to the heart tissue samples resulted in transparent tissue (Figure 1 and Supplementary Figure 1a) which could be imaged using a confocal microscope.

2.2 Tissue preparation

Sectioning: The cleared heart tissue was affixed with superglue along either its short axis or long axis orientation in a specimen tube (Compresstome® VF-300 OZ, Precisionary instruments). The specimen tube was a cylindrical holder with its outer rim fixed with a movable inside platform (or stage). The glued heart tissue was embedded in 2.5% low-melting agarose. For section along the short axis the tissue was placed so that the base of the heart was touching the stage of the specimen tube. For long axis sections, the tissue was kept in a plane on the stage, allowing for its four chambers to be seen. We used the compresstome to obtain $500\mu m$ thick tissue sections with an oscillation frequency of 7 units and a speed of $1.5 mm$/sec. The compresstome blade was kept close to the specimen tube, enabling the compression effect of sectioning to be distributed perpendicular to the sectioning axis. The tissue sections were collected in 1X PBS solution in the buffer tray of the compresstome. Each section was carefully transferred to one of 24 well plates, filled with 1X PBS, while maintaining the sectioning order. For this study, the short axis sections were approximately $3mm$ away from the apex and the long axis sections were approximately $3mm$ from the opposing outer walls of the heart. The

apex sections were cut in the short axis plane from the apical tip of the heart.

Staining: Each 500 µm processed section was washed in 1X PBS thrice over a day and permeabilized using a buffer containing 1% Triton X-100 (H5141, Promega) in PBS (PBST) for one day in a 37 °C incubator shaker. Subsequently, the tissue sections were incubated in 150 µg/mL of Alexa Fluor^{TM} 633 conjugated wheat germ agglutinin (WGA, W21404, ThermoFisher) for one day, to stain the cell membranes. The samples were washed with 1X PBS (3 times for 10 minutes each) before incubating them in imaging media (RIMS). For the preparation of RIMS, 40g of Histodenz (Sigma, D2158) was dissolved in 30 ml of 0.02 M phosphate buffer with 0.01% sodium azide, pH 7.5, resulting in a final concentration of 88% w/v Histodenz. The labelled tissue samples were incubated in RIMS until the tissue became more transparent [9]. All the staining and washing steps were carried out at room temperature, with gentle shaking. The cleared tissue samples were mounted with fresh RIMS solution using spacers (IS002, SUNjin Lab, Taiwan) of 500 µm such that the tissue was sandwiched between coverslips of size $60mm \times 20mm$.

For image acquisition, we used an Olympus FV3000 microscope. Images were first obtained using a lower magnification objective (Olympus PlanApo 1.25X/ air objective) to image a complete area of the heart tissue section. This low-resolution image was used to map high-resolution imaging areas of interest, using the Olympus fluoView^{TM} software. Then, micron scale imaging was carried out with the Olympus UCPLFN 20X Corr M32 85 mm scale air objective (NA=0.73). We used a 640 nm laser line for excitation and FV3000 high sensitivity spectral detectors (gallium arsenide phosphide (GaAsP) photomultiplier tube (PMT)) for detection of emission over a range of $650 - 670nm$. Each field of view covered approximately 320×320 pixels, with a voxel size of $1.98 \times 1.98 \times 1.98$ µm^3 and a depth of $\sim 300\mu m$. Here we under-sampled in the X and Y directions to obtain an isotropic voxel resolution, equivalent to sampling interval in the Z direction. Using the fluoview-map function, we ensured that the acquired 3D images were continuous, and had at least 25% overlap with their respective neighbouring fields of view. To minimize the laser attenuation at deeper regions of the tissue sample, the laser power was corrected (i.e., increased) with the help of the Bright Z function, with a manual judgement based on the quality of the intensity obtained at deeper layers. Each field of view was manually corrected for laser power intensity, increasing this by up to 10% with increased depth. The images were acquired following a snake pattern from row to row. Image reconstructions were performed using computer vision algorithms (as described in Section 3) using custom-written MATLAB and C++ scripts. After imaging, the heart tissue samples were stored in RIMS at room temperature and protected from exposure to light. In order to observe other cell types or Z-disc arrangement (α-actinin) as shown in Extended Data Fig. 1, a 60X magnified imaging of cleared tissue was carried out using PlanApo N 60X oil objective (NA=1.42) with a scan size of 320×320 pixels, with a voxel size of $0.66 \times 0.66 \times 0.79$ µm^3.

2.3 Alignment of different short-axis sections

We aligned the short axis datasets to ensure uniformity across different short axis sections, using the AHA classification and the capillary vessel description for the PSAX-PML level, used in echocardiography studies. We used the SAS3 dataset (Table1) as a reference. First, we aligned the posterior and anterior papillary muscles to the positions of the anterior and inferior regions globally. All other datasets were aligned using the outer wall and papillary muscle morphology to the SAS3 dataset, with the help of a MATLAB script and the ImageJ package. To obtain consistency between section alignments at a coarser scale some datasets were reflected (horizontally or vertically) when needed, using ImageJ. For example, the SAS2 and SAS4 samples both required a vertical reflection. The MATLAB script we wrote takes the SAS3 dataset as a reference and shows it as a transparent layer. This transparent layer can be rotated by an angular value to allow for fine alignment changes. Once the angular value for in-plane rotation had been determined, the short axis datasets were rotated in-plane and saved using ImageJ. The SAS3 and SAS4 had similar morphology. The SAS1 dataset required a $-10\,°C$ in-plane rotation and the SAS2 dataset required a $-7\,°C$ in-plane rotation. The SAS5 dataset was manually rotated by $180\,°C$ in-plane. At the end of this process, all the short axis datasets had a consistent alignment according to the AHA classification, including the positioning of major blood vessels.

2.4 Analysis of short-axis sections from uncleared mouse and rat hearts

We analyzed uncleared hearts from mouse (SAS7) of 1.5 months old and also from a different species, a wild-type Wistar strain of a male rat (RSAS1), approximately 8 weeks in age, which had been scheduled for culling in the NCBS/inStem animal facility. Once the mouse/rat had been sacrificed we performed a similar procedure as described in Section 2 . Once the perfusion was completed, the heart was excised and stored in 4% PFA at $4\,°C$. To enable the tissue to withstand freezing temperatures, the fixed heart was incubated in 30% sucrose for 5hrs before sectioning. The mouse/rat heart was cut into two thick blocks perpendicular to the long axis of the heart (i.e., short-axis-views). The resulting mid-ventricular region was suitable for cryo-sectioning. On the sectioning day, the mouse/rat heart was inserted in a mould containing tissue freezing medium and allowed to solidify at $-20\,°C$. The frozen sample was attached to a holder for cryostat (slee mev+), where the heart specimen was placed perpendicular to the long axis of the heart and sectioned into $100\,\mu m$ slices from the midventricular region. The sections were carefully transferred to 24 well plates containing 1X PBS, and were then washed (3 times for 10 minutes each) to remove freezing media. Afterwards, the tissue sections were incubated in $150\,\mu g/mL$ of Alexa Fluor^{TM}633 conjugated wheat germ agglutinin (WGA, W21404, ThermoFisher) for one day to stain the cell membrane. For α-actinin staining of uncleared or cleared mouse tissue, the tissue was blocked with 2.5% BSA + 2.5% neonatal goat serum in 1X PBST (1% Triton X) for 2hours. 1:200 dilution of primary antibody (Monoclonal anti-α-actinin rabbit antibody, Cell signalling, D6F6, 6487) and 1:400 dilution of secondary antibody (Anti Rabbit alexa-647 Goat antibody, Life technologies, A21245) were used sequentially with an incubation of a day at room

temperature. The samples were washed with 1X PBS (3 times for 10 minutes each) and incubated in RIMS subsequently for another day. We used a positively charged glass slide for mounting the uncleared mouse/rat heart tissue sections and custom made 100 µm spacers (100 µm plastic sheets). The stained heart sections were placed in this glass slide set up with RIMS and sealed with a cover glass in preparation for imaging. We used an Olympus FV3000 microscope and a lower magnification objective (Olympus PlanApo 1.25X/ air objective) to image the complete area of the tissue section to determine the best plane of view. This low-resolution image was used to map the high-resolution imaging area of interest using the Olympus fluoView software. The micron scale imaging was done with the Olympus UCPLFN 20X Corr M32 85 mm scale air objective (NA=0.73). Each field of view consisted of 320×320 voxels per slice, with a voxel size of $1.98 \times 1.98 \times 1.98$ μm^3, over a depth of 10 µm and 50 µm for mouse and rat respectively. As with the cleared mouse hearts, we undersampled in the X, Y directions to obtain isotropic pixels at the resolution of the sampling in the Z direction. We used automatic tile acquisition via the fluoView software platform. Using the fluoview-map function, we ensured that the acquired 3D images were continuous with an overlap of 25 % with their neighbouring tiles, regulated by a motorized stage of the microscope. The fields of view were obtained row by row, following a snake pattern, and were then stitched using custom-built software.

3 Computer Vision Methods

3.1 Deconvolution of acquired data

Since the confocal images were acquired in three dimensions the resulting data was blurred in a manner that depended on the shape of the point spread function (PSF) of the microscope. A pseudo-color image of PSF generated based on our microscope settings is shown in Supplementary Information Figure (SI-Fig.) **S5**. To mitigate the effects of this blur we deconvolved each tile using an iterative Richardson-Lucy (RL) deconvolution method, with Total variation (TV) regularization (RL-TV), as described in [2]. The algorithm minimized the following objective function:

$$\min_{I} ||I \circledast PSF - I_o||_2 + \lambda ||I||_{TV}, \tag{1}$$

where $\circledast$ represents the convolution operation and TV is the total variation norm. In our implementation we set $\lambda = 0.01$ and processed each field of view for 20 iterations.

3.2 Denoising

As depth in the tissue increased, the signal to noise ratio decreased. As a result, the visual quality of the deeper layers was poorer than that of the shallow layers. To mitigate this effect we applied an unsupervised dictionary based method for denoising the images following the deconvolution stage. The method was based on the assumption that layers in the tissue are self similar so that the ultrastructure

of the tissue is similar in different depth layers of a single field of view. We trained a sparse ($m = 256$) element 2D dictionary of patches of size 16×16 using the sparse dictionary learning method of [6]. The shallow layers were relatively free from both depth and other optical degradation effects. The dictionary (D) was learned from data samples (x_i) from the shallow layers using the alternating minimization approach in [7]. The method involved alternating between between fixing D and solving the resulting basis pursuit denoising problem in Eq. (2) below

$$\min_{D \in C, \alpha \in \mathbb{R}^{256 \times n}} \frac{1}{n} \sum_{i=1}^{n} \left(\frac{1}{2} ||x_i - D\alpha_i||_2^2 + \lambda ||\alpha_i||_1 \right), \tag{2}$$

and fixing α and updating the dictionary D using coordinate descent. Here, $C = \{D \in \mathbb{R}^{256 \times 256}\ s.t.\ \forall j,\ ||d_j||_2^2 \leq 1\}$, and α_i are the sparse codes corresponding to data element x_i. We used a value of $\lambda = 0.15$ as the regularization parameter in our experiments and ran the optimization for 1000 iterations. The set of learned dictionary patches using tissue samples from the SAS1 dataset is shown in SI-Fig. **S6**.

The images acquired from deeper layers in the tissue sample can then be denoised using this learned sparse dictionary. To accomplish this, at each voxel in a degraded image we constructed a 16×16 patch centered at the voxel and estimated a denoised patch α by solving the sparse coding problem in Eq. (3) using a lars/homotopy method [3]:

$$\min_{\alpha \in \mathbb{R}^{256}} \frac{1}{2} ||x - D\alpha||_2^2 + \lambda ||\alpha||_1. \tag{3}$$

The final denoised image was reconstructed as an average of the denoised patches of the overlapping windows at each voxel. We learned a separate dictionary for each field of view so that the structure in one field of view did not affect the reconstruction in another.

3.3 Stitching 3D blocks

Each tissue section was too large to be imaged at once so we imaged multiple square shaped fields of view (tiles) of 320×320 isotropic voxels of length $1.98\mu m$ in each dimension, in the regions containing tissue samples. Adjacent tiles were set to have an overlap of 12.5% in every direction. We used the image registration method described in [8] for regions with valid data. The method involved a two stage registration process, with a local pairwise registration followed by a global registration. In the first local registration stage, we started with an initial guess for the location of each tile, derived from the microscope stage settings and assumed a 40 voxel (12.5%) overlap value. For every pair of adjacent tiles (a, b) we used the maximum phase correlation[5] based registration to estimate the relative shift, p_{ab} between the pair.

$$p_{ab} = \arg\max \mathscr{F}^{-}1 \left(\frac{A(\omega)B^*(\omega)}{|A(\omega)B^*(\omega)|} \right) \tag{4}$$

where, $\mathscr{F}^{-1}$ represents the inverse Fourier transform and $A(\omega)$ and $B^*(\omega)$ are the Fourier transform and the complex conjugate of the Fourier transform of tile a and tile b, respectively. For every imaged tile, a shift was computed with each of its 4-neighbours in the 2D imaging plane. This local pairwise registration process resulted in a refined list of (p_{ab}) pairwise relative shift values.

In the second global registration stage, a global optimal tile location of each tile $(p_a, p_b, ...)$ was computed with respect to the top left corner of the image. In all our datasets this corner tile was empty and was only used to define a common reference frame. The vector of optimal tile positions P for all tiles $T = \{a, b, ..\}$ was then given by

$$P = \arg\min \sum_{a \in T} \left(\sum_{b \in \mathcal{N}(a)} c_{ab} ||p_b - p_a - p_{ab}||_2^2 \right) \tag{5}$$

where c_{ab} was the correlation value between the pair a, b. This global registration was accomplished by solving an over-determined system of sparse linear equations for position [10]. This was done by iteratively eliminating all pairs of outlier pairwise distances. A local shift was labelled an outlier if it was over three standard deviations away from the mean shift.

Figures S2B and S2C illustrate the stitching process for a short axis section of a mouse heart. Two sample tiles are demarcated by red and blue bounding boxes.

3.4 Orientation field estimation

The orientation field was estimated at each voxel in the stitched and denoised image stack. We used the structure tensor [4] $s = G_\rho \circledast (\nabla_\sigma I)(\nabla_\sigma I)^T$, where G_ρ is a Gaussian with standard deviation ρ, $\circledast$ is the convolution operation and ∇_σ represents the intensity gradient at a Gaussian smoothing scale of σ. We used a noise scale $\sigma = 0.5$ and feature scale of $\rho = 3$ voxel units. The orientation was then set to align with the eigenvector of the structure tensor corresponding to the eigenvalue with smallest magnitude.

3.5 Computation of the Helix Angle

The helix angle α_H is typically defined in a manner that is relative to the local direction normal to the outer heart wall. To estimate the wall normal we computed a single pixel wide boundary of the heart in a short axis section and fit a circle tangential at each point along the outer boundary, using 80 sample points along the boundary in each direction. The direction of the heart wall normal was then associated with the inward radial vector of the circular fit (Figure S3B). The local helix angle α_H, as illustrated in Figure 2A, was calculated using the projection of the orientation onto the tangential plane defined by the heart wall normal. The angle varied from $-90°$ to $90°$, with $0°$ representing the in plane circumferential fibers and $\pm 90°$ representing fibers pointing out of the short axis plane, in the long axis direction of the heart.

3.6 Smoothing the Estimated Orientation Field

Given the thickness of tissue samples used in our study, imaging data in deeper layers, where light penetration was reduced, was noisy. In addition, optical factors including light scattering, photo bleaching and optical aberrations in the microscope lens, also diminished the image quality. To mitigate the affect of the reduced image quality on orientation estimation we averaged the orientation field over

small neighborhoods. Whereas orientations are directionless, their representation using the eigenvector with the smallest eigenvalue of the structure tensor is not. Two vectors whose components have the same magnitude but differ in signs represent the same orientation, so these direction vectors cannot be directly averaged component wise.

To smooth the orientations we first computed the rank-1 tensor constructed as $s = uu^T$, where s is a 3×3 matrix and u was the local unit direction vector. This rank-1 tensor was invariant to flips, since $s = uu^T = (-u)(-u)^T$. In fact, $s \in Gr(3,1)$, represents the Grassmann manifold of one dimensional subspaces (lines) in 3 dimensional Euclidean space. While it was possible to use the weighted Karcher mean to smooth the resulting tensors component-wise, an iterative approach to doing so was slow and did not scale well to handle large volumes of data. We therefore opted for an approximate strategy. We averaged the orientation tensors s component-wise using a local weighted average, and then projected back to the space of direction vectors. Specifically, the smoothed tensor $\hat{s}$ at any location $\mathbf{x}$ was given by

$$\hat{s}(\mathbf{x}) = \sum_{y \in Nbd(\mathbf{x})} w(\mathbf{y}-\mathbf{x}) s(\mathbf{y}). \quad (6)$$

Here, $w(\cdot)$ is a scalar weight, which was empirically chosen to be a Gaussian with $\sigma = 4$, as defined below. The smoothed direction vector $\hat{u}$ at $\mathbf{x}$ was then given by the eigenvector of the $\hat{s}$ matrix corresponding to the eigenvalue with the largest magnitude. We only carried out smoothing in regions within the heart tissue by setting the weight $w(\mathbf{z})$ to zero in regions with missing data:

$$w(\mathbf{z}) = \begin{cases} \frac{1}{\sqrt{(2\pi\sigma^2)^3}} e^{-\frac{\mathbf{z}^2}{\sigma^2}} & \text{if } \mathbf{z} \in Supp(data) \\ 0 & \text{otherwise.} \end{cases} \quad (7)$$

3.7 Validating Orientation Estimates

To verify the accuracy of the orientations estimated using the structure tensor we hand segmented 70 myocytes in a field of view from the dataset SAS3, by manually tracing the boundaries of individual cells across multiple slices. The myocytes were marked by labeling the cytoplasm within each cell boundary, as signalled by WGA staining. The orientation of each hand segmented cell was then calculated using the second moment matrix of the interior voxels of each labeled myocyte. Each hand segmented cell was assigned a ground truth cell orientation derived from the eigenvector corresponding to the eigenvalue of the second moment matrix with the largest magnitude. We then associated a structure tensor based orientation estimate for the segmented cell using the average of the estimated field orientations over each of its interior voxels. A cell wise comparison revealed close agreement between the ground truth and the structure tensor based estimates of the orientations from the raw WGA images. For 70 hand segmented cells we observed an average difference of $6.13°$ with a standard deviation of $3.56°$. Figure 1 and Supplementary Figure 3a provide a comparison of the ground truth myocyte orientations, represented by purple cylinders, and the structure tensor based estimated orientations, represented by golden yellow cylinders, with the manually segmented myocytes shown

in gray. We computed the Fractional Anisotropy (FA) score for each tensor, to confirm that the orientation estimates based on it were valid in that they reflected local elongation. The FA score as defined below in equation (8) measures the degree of anisotropy of the structure tensor, where the λ_i's are the eigenvalues of the structure tensor matrix.

$$FA = \sqrt{\frac{1}{2}} \frac{\sqrt{(\lambda_1 - \lambda_2)^2 + (\lambda_2 - \lambda_3)^2 + (\lambda_3 - \lambda_1)^2}}{\sqrt{\lambda_1^2 + \lambda_2^2 + \lambda_3^2}}. \tag{8}$$

An FA score close to one indicates strong directionality of the gradients at a location, while a value closer to zero reflects an isotropic region. The computed FA values were found to be closer to 1 for most of the tissue section, confirming that the WGA data reflected locally elongated cardiomyocytes.

3.8 Φ, θ and α_H calculation and colormap generation

The colormaps for Φ, θ and α_H were generated using the smoothed orientation field. We calculated the Φ and θ angles at each voxel with valid data and then mapped these angles to color values using a linear scale in the parula colormap.

To obtain the α_H colormaps we considered voxels with valid data and then computed the average value of the helix angle over all radial penetration lines overlapping at the voxel. A penetration line at a point (x,y) in a short axis plane was considered to overlap an integer valued voxel (i,j) when $i \leq x \leq i+1$ and $j \leq y \leq j+1$. Due to irregularities in boundary shape, which in turn affected the estimate of the heart wall normal, it was possible for a few isolated voxels, containing valid orientation estimates, to have no penetration line passing through them. The α_H value at each such location was set to the average α_H value over a 3×3 voxel neighbourhood.

3.9 Computing the transmural rate of change of α_H

To measure the rate of change of α_H in the transmural direction we compute the forward difference between its values along the outer to inner wall direction. This difference is computed by calculating the smaller of the angle between two successive α_H vectors. To ensure that these rate of change estimates are well defined, we smoothed α_H using a 1D Gaussian with $\sigma = 7$ voxels, along the transmural penetration direction, prior to computing the forward difference.

3.10 3D Rendered Visualizations and Animations

Orientation glyphs and streamlines were procedurally generated and rendered using a custom Python module within the open-source animation software Blender. For each sample volume, an orientation field and a tiff stack representing the WGA tissue staining were imported as N-dimensional NumPy arrays.

Glyphs The orientation field was represented by a 3-dimensional array of rotated cylinders, referred to as orientation glyphs. The input vector field was approximated by a 3-dimensional grid of equally spaced vertices, downsampled such that the number of vertices was not larger than 75,000. At each vertex of the downsampled grid, a cylinder primitive shape was created, and rotated proportionally to the components of the vector field at the vertex position. A parula colormap was applied to the cylinders proportional to the Φ angle, defined as the arc cosine of the absolute value of the z component of the vector field.

Streamlines Bidirectional streamlines were represented as curves extruded from polylines, whose points were computed as follows. A set of up to 25,000 points were selected from a random sample of voxels from the vector field, and each sample voxel location was the initial point for a streamline. Since the direction of the vector field represents the orientation of the tissue, and the sign of the orientation does not matter, each starting point initialized both a positive and negative streamline. Both positive and negative streamlines originated from a single starting point, and were grown by iteratively adding new points along the polyline. For each iteration, the location of the next point was calculated by adding a displacement proportional to the vector field and the current point, multiplied by the sign, such that the positive streamline is displaced by the positive value of the field, and the negative streamline by the negative value of the field. At each new position, the value of the field at that voxel acts to determine the position of the following point along the polyline. A parula colormap was applied to the streamlines in proportion to the Φ angle at the streamlines starting point within the vector field. To avoid artifacts arising where the streamlines extend beyond tissue, a binary mask of the tissue volume with the same dimensions as the vector field served as a boundary condition.

Appendix Table S1

<table>
<tr><th>Dataset</th><th>Orientation of dataset</th><th>Animal Sex</th><th>Derived from animal number</th><th>Cleared (by CLARITY) or Uncleared</th></tr>
<tr><td>SAS1*
SAS2*
SAS3*
SAS4*</td><td rowspan="4">Short Axis</td><td rowspan="7">Female</td><td>Mouse 1</td><td rowspan="3">Cleared</td></tr>
<tr><td>SAS5</td><td>Mouse 2</td></tr>
<tr><td>SAS6</td><td>Mouse 3</td></tr>
<tr><td>SAS7</td><td>Mouse 4</td><td>Uncleared</td></tr>
<tr><td>LAS1#
LAS2#
LAS3#
LAS4#</td><td rowspan="2">Long Axis</td><td>Mouse 5</td><td rowspan="3">Cleared</td></tr>
<tr><td>LAS5</td><td>Mouse 6</td></tr>
<tr><td>APEX1*
APEX2*
APEX3*
APEX4*</td><td>Short Axis</td><td>Mouse 1</td></tr>
<tr><td>RSAS1</td><td>Short Axis</td><td>Male</td><td>Rat 1</td><td>Uncleared</td></tr>
</table>

Appendix Table S1: A list of sections and animals used in this study (*/# indicate serial sections from same animal)

References

[1] Kwanghun Chung, Jenelle Wallace, Sung-Yon Kim, Sandhiya Kalyanasundaram, Aaron S Andalman, Thomas J Davidson, Julie J Mirzabekov, Kelly A Zalocusky, Joanna Mattis, Aleksandra K Denisin, et al. "Structural and molecular interrogation of intact biological systems". In: *Nature* 497.7449 (2013), pp. 332–337.

[2] Nicolas Dey, Laure Blanc-Feraud, Christophe Zimmer, Pascal Roux, Zvi Kam, Jean-Christophe Olivo-Marin, and Josiane Zerubia. "Richardson–Lucy algorithm with total variation regularization for 3D confocal microscope deconvolution". In: *Microscopy research and technique* 69.4 (2006), pp. 260–266.

[3] Iddo Drori and David L Donoho. "Solution of L1 minimization problems by LARS/homotopy methods". In: *2006 IEEE International Conference on Acoustics Speech and Signal Processing Proceedings*. Vol. 3. IEEE. 2006, pp. III–III.

[4] Hans Knutsson, Carl-Fredrik Westin, and Mats Andersson. "Representing local structure using tensors II". In: *Scandinavian conference on image analysis*. Springer. 2011, pp. 545–556.

[5] C.D. Kuglin and D.C. Hines. "The phase correlation image alignment method". In: *Proc. Int. Conf. on Cybernetics and Society*. IEEE, Sept. 1975, pp. 163–165.

[6] Julien Mairal, Francis Bach, Jean Ponce, et al. "Sparse modeling for image and vision processing". In: *Foundations and Trends® in Computer Graphics and Vision* 8.2-3 (2014), pp. 85–283.

[7] Julien Mairal, Francis Bach, Jean Ponce, and Guillermo Sapiro. "Online dictionary learning for sparse coding". In: *Proceedings of the 26th annual international conference on machine learning*. 2009, pp. 689–696.

[8] Stephan Preibisch, Stephan Saalfeld, and Pavel Tomancak. "Globally optimal stitching of tiled 3D microscopic image acquisitions". In: *Bioinformatics* 25.11 (2009), pp. 1463–1465.

[9] Bin Yang, Jennifer B Treweek, Rajan P Kulkarni, Benjamin E Deverman, Chun-Kan Chen, Eric Lubeck, Sheel Shah, Long Cai, and Viviana Gradinaru. "Single-cell phenotyping within transparent intact tissue through whole-body clearing". In: *Cell* 158.4 (2014), pp. 945–958.

[10] Dženan Zukić, Michael Jackson, Dennis Dimiduk, Sean Donegan, Michael Groeber, and Matthew McCormick. "ITKMontage: A Software Module for Image Stitching". In: *Integrating Materials and Manufacturing Innovation* 10.1 (2021), pp. 115–124.
