# Supplementary material for: Cardiomyocyte orientation recovery at micrometer scale reveals long‐axis fiber continuum in heart walls
Source: EMBO J. 2023 Sep 6;42(19):e113288. doi: 10.15252/embj.2022113288 (PMC10548172; doi:10.15252/embj.2022113288)
Supplement: Supplementary file 2 — Expanded View Figures PDF [file EMBJ-42-e113288-s008.pdf]

Expanded View Figures

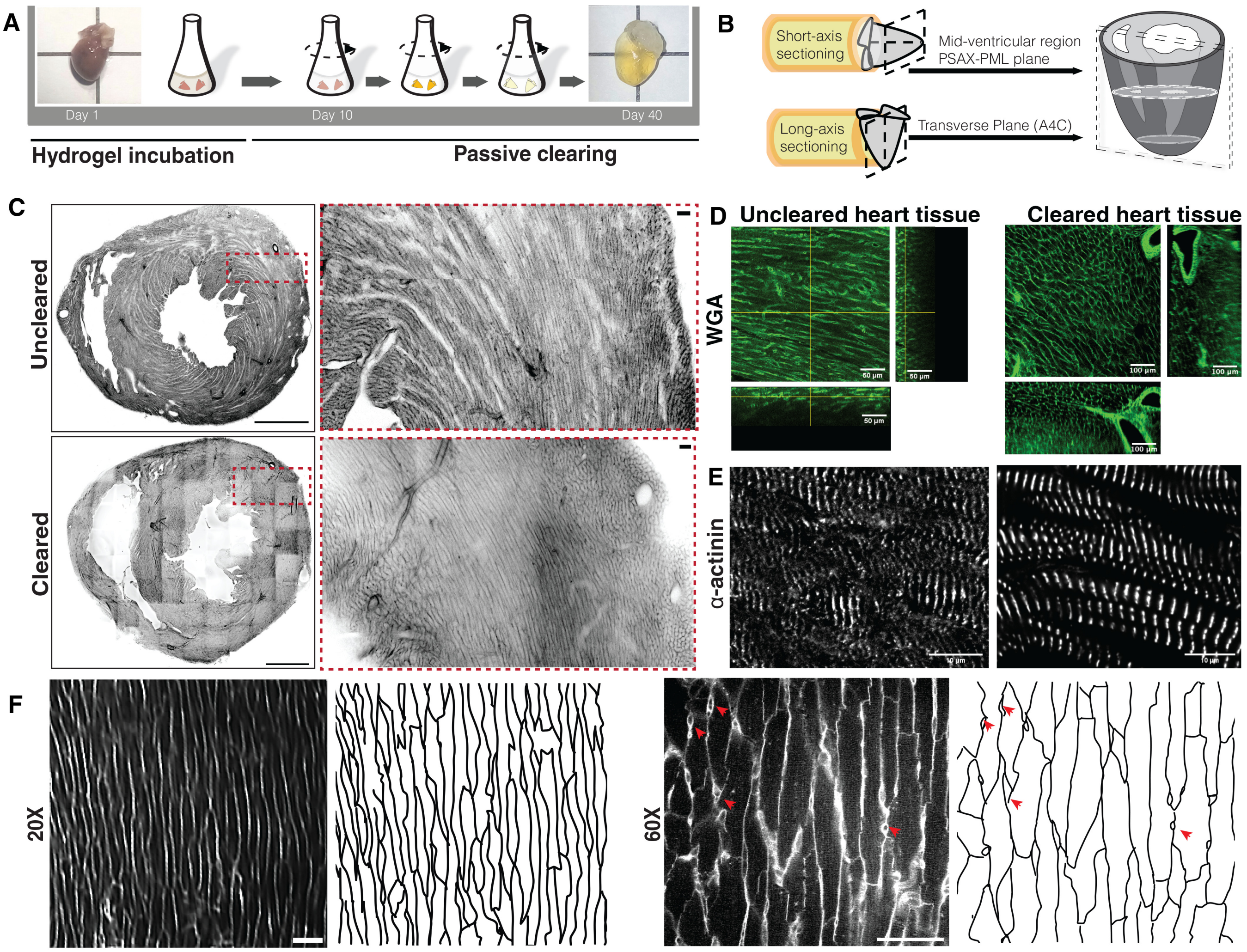

Figure EV1. A comparison of cleared and uncleared mouse heart tissue by imaging.

A A schematic illustration of the CLARITY method. The harvested heart tissues are incubated in a hydrogel/PFA mixture at 4°C for approximately 9 days. Following hydrogel incubation, the solution mixture with the heart tissues is polymerized at 37°C. The heart tissues are then excised from the polymerized hydrogel and shaken at 37°C with a clearing solution until they attain a desirable level of transparency (see Materials and Methods for details).

B The clarified heart is sectioned along its short- or long-axis. For the short-axis, a midventricular region that approximates the PSAX-PML (parasternal short-axis—papillary muscle level) plane was chosen. For the long-axis, a transversal plane that represents the A4C (apical four chambers) plane was used.

C–E A comparison of uncleared and cleared heart tissue sections stained with WGA and the alpha-actinin antibody.

F A comparison of 20× (2-µm isotropic resolution) and 60× (0.663 * 0.663 * 0.79 μm^3 x, y, and z resolution, respectively) cleared heart tissue sections stained with WGA. The raw and skeletonized images are shown for each magnification. The visible cell boundaries that are not of cardiomyocytes are marked with arrow heads (red) in the 60× images. The scale bar is 1,000 and 50 µm for the full view and zoomed in regions, respectively, in the WGA uncleared and cleared tissue images. The scale bar for the alpha-actinin images is 10 µm.

 © 2023 The Authors

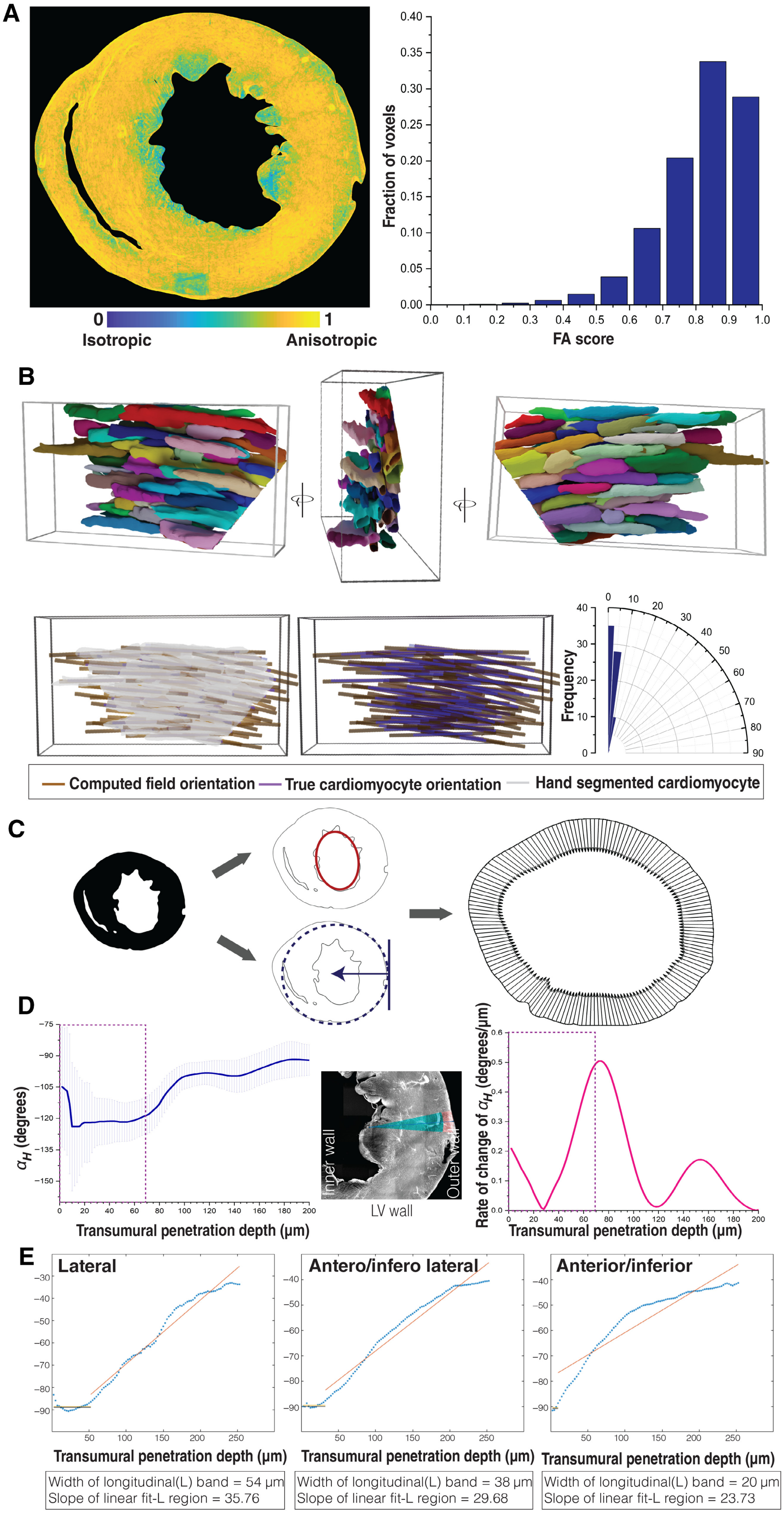

Figure EV2. The structure tensor method for orientation estimation.

A Fractional anisotropy scores are shown using a parula colormap (left) with a value in the range 0–1. The histogram (right) shows the fraction of pixels in each respective FA bin. The majority of the pixels have a high FA score, indicating the presence of dominant local orientations in the tissue stack. The color bar for the FA score is as indicated.

B Representative 3D views of hand-segmented cardiomyocytes with randomly assigned colors to each cell (top panel). A representative 3D view of ground truth orientation (purple) based on the second moment matrix for hand-segmented myocytes (gray) on the left, with the estimated field orientation from the WGA image (golden yellow) using a structure tensor approach (Materials and Methods) in the middle (bottom panel). The magnitude of the difference between the ground truth and the estimated orientation in degrees is illustrated with a graph on the right. The mean difference between two ground truth and estimated orientations is 5.98° ± 2.3°.

C Helix angle calculation: Masking (left) followed by centroid estimation (middle, top); Masking the short-axis section and estimating the tangent plane and normal for the penetration axis (middle, bottom). The set of penetration directions is shown on the right, from outer to inner wall.

D αH plot (left), region of LV wall analyzed (middle) and rate of change of αH calculated over a neighborhood of 15 voxels. The region marked by a dashed box represents the outer wall longitudinal cells, where the rate of change of αH is initially small and then increases sharply as one approaches the middle wall region, after which it plateaus and then increases again.

E Line fits for αH plots were calculated for the lateral, antero/infero lateral, and anterior/inferior regions, with the extent of the outer wall longitudinal cells shown by length of the first line in each plot.

© 2023 The Authors

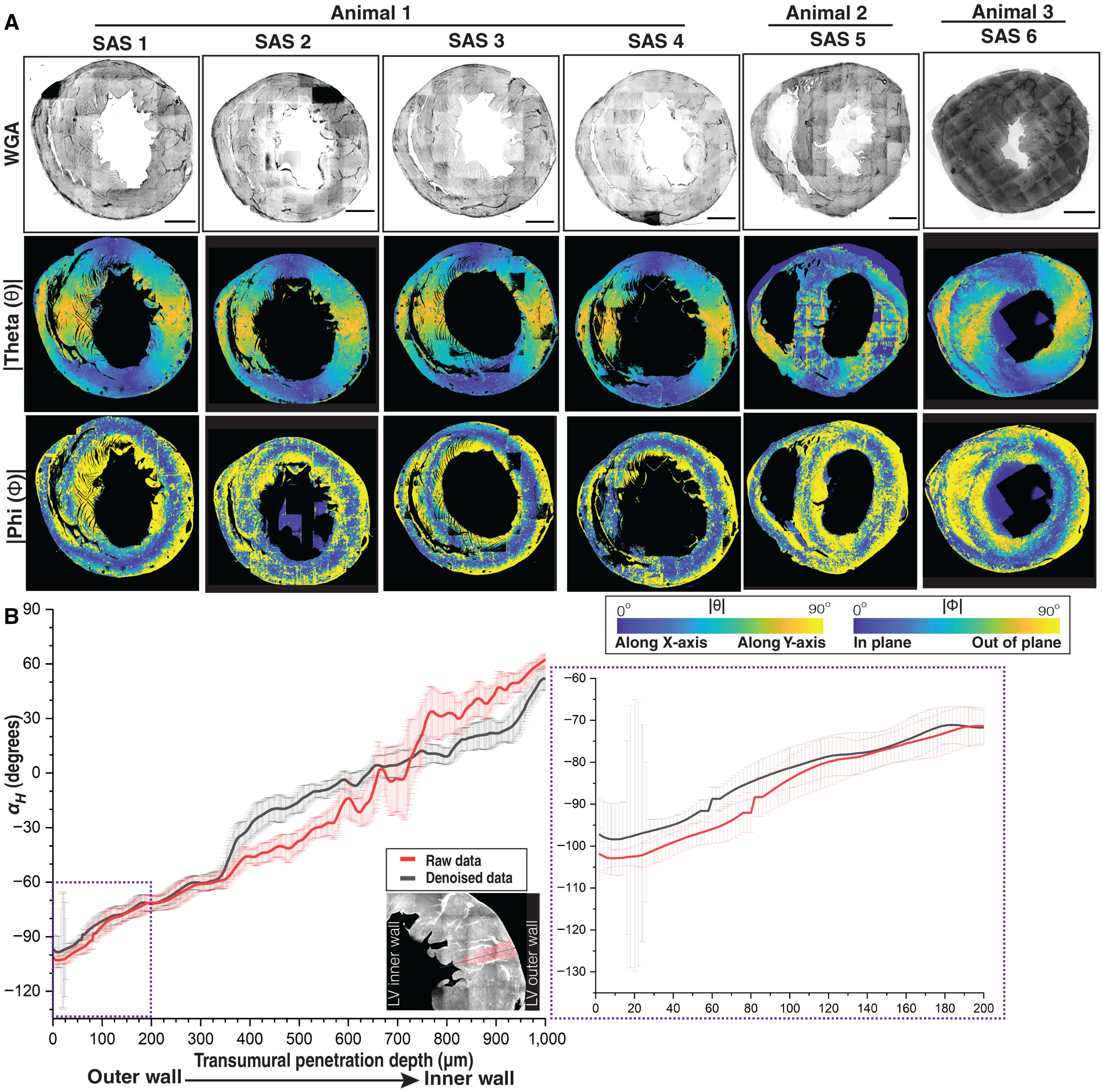

Figure EV3. WGA staining and angular colormaps of different short-axis sections.

A A maximum intensity Z-projection of the WGA-stained short-axis section from different mouse hearts as indicated, shown in grayscale, with the |Φ| and |θ| angles for cell orientations shown using parula colormaps, with the color bars as indicated. The scale bar is 1,000 μm.

B A comparison of αH estimates from raw (red line) and denoised short-axis section image stacks (black line) are shown for comparison. The region of the LV wall analyzed is shown in the bottom left panel, with a zoom in on the first 200 μm on the bottom right. The transmural penetration direction is from the outer to the inner LV wall. The raw data agree with denoised αH estimates in most of the places; however, we observed that estimation was much more consistent and robust to nonuniform illumination changes with denoised data. The intensity between two fields of view (~ 350 μm in transmural penetration depth) and the αH estimates begin to deviate marking the edges.

© 2023 The Authors

Figure EV4. Analysis of a short-axis section of a rat heart.

A A short-axis view of the ventricular chambers of a rat heart, sectioned at PSAX-PML (parasternal short-axis—papillary muscle level). A maximum intensity projection of the WGA stain is shown in grayscale, with the |Φ| angle shown using a parula colormap. The scale bar is 1,000 μm.

B–D Zoomed in regions from the left ventricle, septum and right ventricle, with the WGA stain shown in grayscale and the |Φ| angle shown using a parula colormap. The scale bars are 1,000 μm 100 μm for (A) and (B–D), respectively. The color bar is as indicated.

© 2023 The Authors

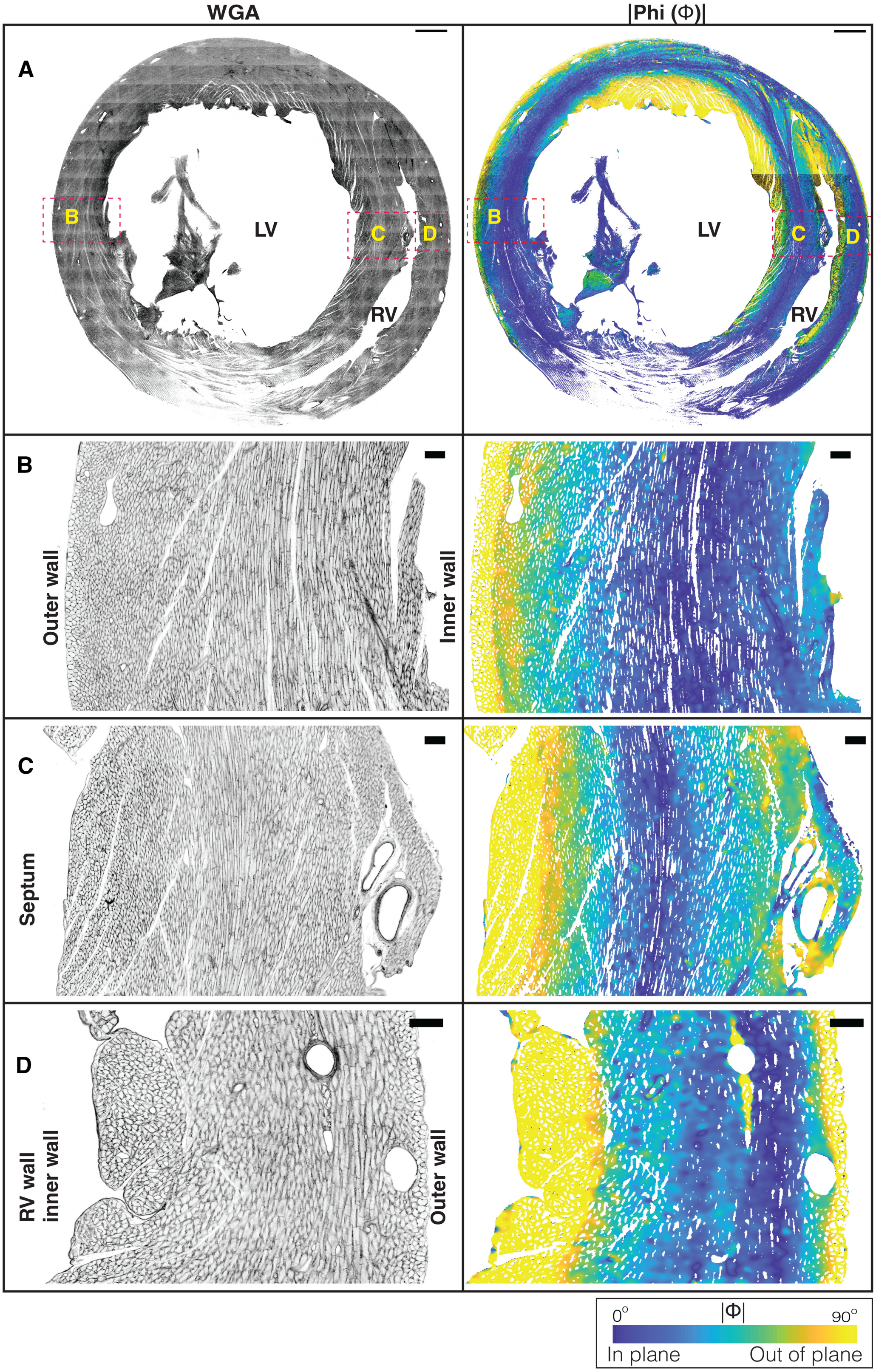

Figure EV4.

 © 2023 The Authors

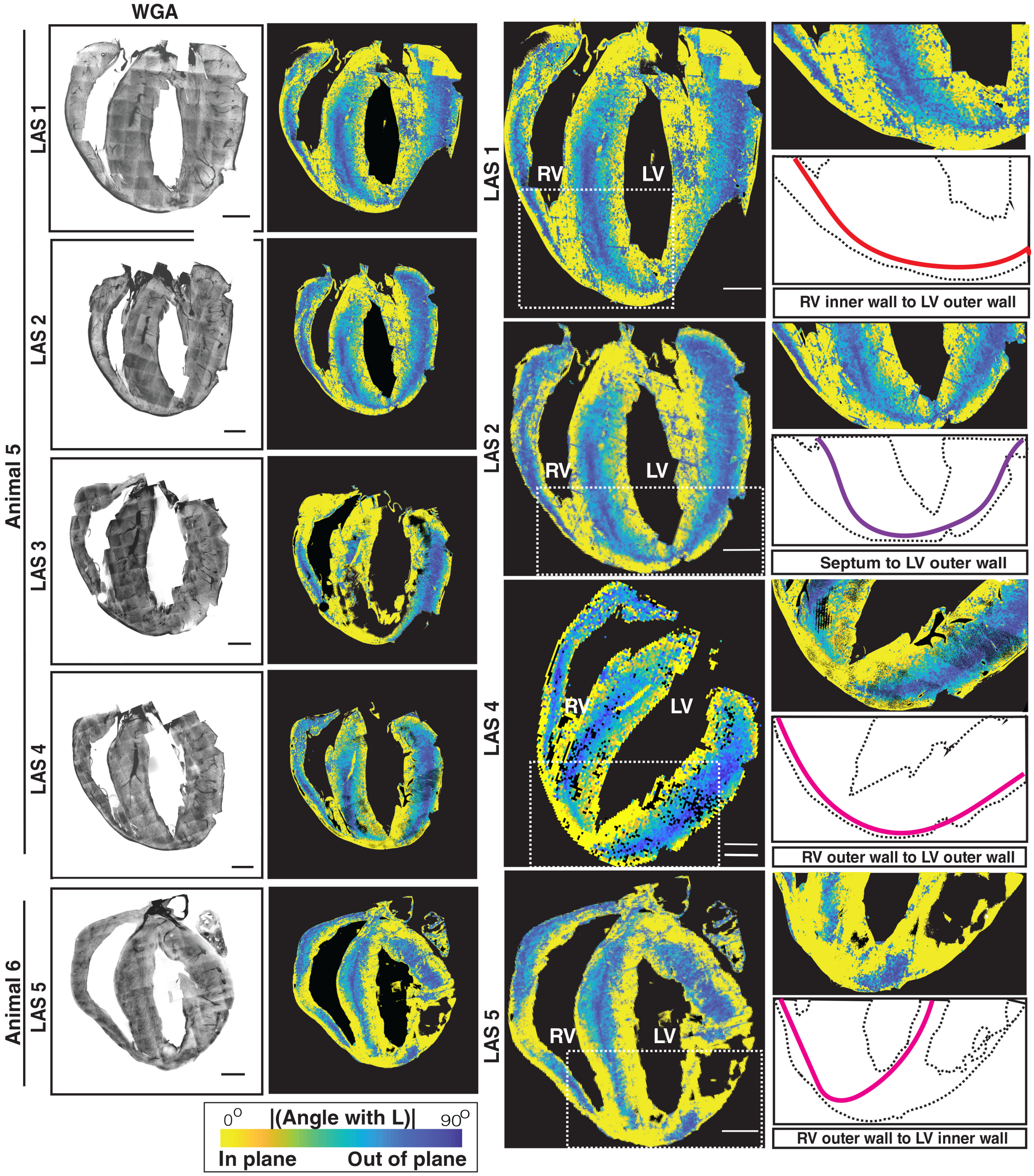

Figure EV5. WGA staining and colormaps for the magnitude of the angle with the longitudinal axis, for different long-axis sections and their connections at the apex.

Five mouse heart ventricle long-axis sections are shown as maximum intensity Z-projections of the WGA stain, with the magnitude of the angle between the aggregate cell orientation and longitudinal axis shown with a parula colormap marked, with a white dotted box highlighting the apex region under consideration (left column). A zoomed-in view of the highlighted apex region from the respective long-axis sections and illustration of long-axis connections between different ventricular walls is also shown (right column). The continuity of the long-axis fibers from the septum to the left ventricle outer wall, from the right ventricle inner wall to the left ventricle outer wall, from the right ventricle outer wall to the left ventricle outer wall and from the right ventricle inner wall to the left ventricle inner wall, is highlighted in the first, second, third, and fifth rows, respectively. The colorbar is as indicated, and the scale bar is 1,000 μm.

© 2023 The Authors
