# Supplementary material for: Cardiomyocyte orientation recovery at micrometer scale reveals long‐axis fiber continuum in heart walls
Source: EMBO J. 2023 Sep 6;42(19):e113288. doi: 10.15252/embj.2022113288 (PMC10548172; doi:10.15252/embj.2022113288)
Supplement: Supplementary file 11 — PDF+ [file EMBJ-42-e113288-s005.pdf]

Article

TRANSPARENT PROCESS OPEN ACCESS

THE EMBO JOURNAL

Cardiomyocyte orientation recovery at micrometer scale reveals long-axis fiber continuum in heart walls

Drisya Dileep[1,2,†], Tabish A Syed[3,†], Tyler FW Sloan[4], Perundurai S Dhandapany[1], Kaleem Siddiqi[3,*] & Minhajuddin Sirajuddin[1,**]

Abstract

Coordinated cardiomyocyte contraction drives the mammalian heart to beat and circulate blood. No consensus model of cardiomyocyte geometrical arrangement exists, due to the limited spatial resolution of whole heart imaging methods and the piecemeal nature of studies based on histological sections. By combining microscopy and computer vision, we produced the first-ever three-dimensional cardiomyocyte orientation reconstruction across mouse ventricular walls at the micrometer scale, representing a gain of three orders of magnitude in spatial resolution. We recovered a cardiomyocyte arrangement aligned to the long-axis direction of the outer ventricular walls. This cellular network lies in a thin shell and forms a continuum with longitudinally arranged cardiomyocytes in the inner walls, with a complex geometry at the apex. Our reconstruction methods can be applied at fine spatial scales to further understanding of heart wall electrical function and mechanics, and set the stage for the study of micron-scale fiber remodeling in heart disease.

Keywords 3D reconstruction; cardiomyocyte geometry; computer vision; fluorescent microscopy; heart wall structure

Subject Categories Cardiovascular System; Computational Biology; Methods & Resources

DOI 10.15252/embj.2022113288 | Received 14 December 2022 | Revised 1 August 2023 | Accepted 6 August 2023 | Published online 6 September 2023

The EMBO Journal (2023) 42: e113288

Introduction

The mammalian heart wall is densely packed with cardiomyocytes that are geometrically aligned end-on-end to constitute cardiac muscle (Cretoiu *et al*, 2018). The geometric organization of cardiomyocytes supports synchronous contraction and electrical conduction while also offering mechanical strength (Streeter *et al*, 1969; LeGrice *et al*, 1995; Gilbert *et al*, 2007; Young & Panfilov, 2010; Savadjiev *et al*, 2012; Aumentado-Armstrong *et al*, 2018; Libby *et al*, 2018). Malformations in cardiomyocyte arrangement and heart tissue fibrosis can lead to pathological conditions of the heart, including cardiomyopathies, remodeling following myocardial infarction, and disorders related to electrical propagation (Geerts-Ossevoort *et al*, 2001; Chen *et al*, 2003; von Deuster *et al*, 2016). At a coarse spatial scale, the geometric organization of cardiomyocytes has been described as a helical continuum, wrapping around the chambers of the heart (Sallin, 1969; Streeter *et al*, 1969; Scollan *et al*, 1998; Beg *et al*, 2004; Chen *et al*, 2005; Gilbert *et al*, 2007; Rohmer *et al*, 2007; Bayer *et al*, 2012; Savadjiev *et al*, 2012; Libby *et al*, 2018). However, several competing models of cardiac myofiber organization still exist (Gilbert *et al*, 2007; Anderson *et al*, 2009), including a three-layer model (Rushmer *et al*, 1953) and characterizations as nested donuts (Streeter *et al*, 1969), toroids (Peskin, 1989), pretzels (Jouk *et al*, 2000), or a single helical band (Corno *et al*, 2006).

Despite this lack of consensus, the present models of heart wall cardiomyocyte geometry in use for cardiovascular research are largely derived from millimeter resolution diffusion-tensor magnetic resonance imaging (DT-MRI; Peskin, 1989; Horowitz *et al*, 1993; Helm *et al*, 2005; Gilbert *et al*, 2007; Savadjiev *et al*, 2012). These models all support a smooth clockwise rotation of cardiomyocytes in a transmural penetration from outer to inner wall and do not predict any singularities in their aggregate orientation. Rule-based models of cardiac myofiber orientation (Bayer *et al*, 2012) as well as those based on minimal surfaces (Savadjiev *et al*, 2012) also exist, but these are largely consistent with those afforded by DT-MRI. DT-MRI has also been used to obtain statistical atlases of heart wall myofiber geometry from the imaging of multiple subjects (Peyrat *et al*, 2007; Lombaert *et al*, 2012). Whereas such models can advance studies of heart wall electrical and mechanical function (Vetter *et al*, 2005; Young & Panfilov, 2010), they all suffer from limitations in spatial resolution. In fact,

1 Centre for Cardiovascular Biology and Disease, Institute for Stem Cell Science and Regenerative Medicine, Bengaluru, India
2 The University of Trans-Disciplinary Health Sciences and Technology (TDU), Bengaluru, India
3 School of Computer Science and Centre for Intelligent Machines, McGill University, and MILA – Québec AI Institute, Montréal, QC, Canada
4 Quorumetrix Studio, Montréal, QC, Canada
*Corresponding author. Tel: +1 5143983371; E-mail: siddiqi@cim.mcgill.ca
**Corresponding author. Tel: +91 8061948133; E-mail: minhaj@instem.res.in
†These authors contributed equally to this work as first authors

© 2023 The Authors. Published under the terms of the CC BY 4.0 license.

hundreds of cardiomyocytes can occupy a single voxel at this millimeter scale.

Micron-scale light microscopy methods have the potential to recover orientation at the scale of individual cardiomyocytes. However, such efforts have concentrated thus far on imaging small sections (Seidel *et al*, 2016) or 3D stacks of heart tissue (Pope *et al*, 2008; Sivaguru *et al*, 2015; Nehrhoff *et al*, 2017; Perbellini *et al*, 2017; Teh *et al*, 2017; Garcia-Canadilla *et al*, 2019; Merz *et al*, 2019) and not on modeling cardiomyocyte or myofiber geometry at the organ scale. Approaches based on histological sections are typically limited to two-dimensional imaging (Greenbaum *et al*, 1981; Young *et al*, 1998; Anderson *et al*, 2009). Such methods also have not recovered the orientation of individual cardiomyocytes or their aggregate orientation at the whole heart scale. Thus, the geometric organization of cardiomyocytes at the micron scale across entire heart walls remains a fundamental unaddressed question in organ biology.

To tackle this problem, we developed new tissue structure orientation analysis methods by combining confocal light microscopy-based deep and wide imaging with computer vision techniques. Our methods extract information from the fluorescence signal at cardiomyocyte boundaries, where the intensity gradient provides unbiased estimates of the eigenvectors associated with the structure tensor. This allowed us to estimate micron-scale cardiomyocyte orientations across entire long-axis and short-axis ventricular sections from mouse hearts. Our three-dimensional reconstructions at unprecedented spatial resolution revealed never before reported long-axis bands of fibers extending along the entire length of the outer ventricular walls, different from the known smoothly varying helical arrangements (Scollan *et al*, 1998; Chen *et al*, 2005; Gilbert *et al*, 2007; Bayer *et al*, 2012; Savadjiev *et al*, 2012). This finding could advance present understanding of heart wall mechanics and electrical conduction, as well as open new opportunities to interrogate heart wall tissue structure and diseases related to alterations of cardiomyocyte and hence myofiber organization, across a range of spatial scales.

Results

An integrated pipeline for estimating micron-scale cell orientations from *ex vivo* tissue

To employ fluorescence based deep tissue imaging, we applied a tissue clearing method based on the CLARITY protocol (Tomer *et al*, 2014), optimizing it for *ex vivo* heart tissue (Materials and Methods; Figs 1 and EV1A). The intact cleared hearts from normal mice were serially sectioned in short- and long-axis views (Fig EV1B), approximately at the parasternal short axis-papillary muscle (PSAX-PML) and the horizontal long axis-4 chamber (HLA-4C) regions, respectively, following the American Heart Association (AHA) nomenclature (Cerqueira *et al*, 2002). Post sectioning, the tissue was stained with fluorescent wheat germ agglutinin (WGA), which marks cell membranes (Materials and Methods) (Figs 1 and EV1C and D). The WGA-stained tissue sections were subjected to confocal imaging (Methods) (Fig 1). A comparison with uncleared heart images showed no significant alterations in the tissue due to clearing procedures (Fig EV1D and E). The images were acquired at 2-μm isotropic resolution such that only cardiomyocyte boundaries were captured (Fig EV1F). Every intact short- and long-axis section was imaged in its entirety, as blocks of individual fields of view, each being ~ 640 × 640 × 300 μm in dimension (Materials and Methods) (Fig 1; Appendix Fig S1A). The images from cleared heart tissues were further processed by deconvolution, denoising, and stitching algorithms to recover micron-scale resolution for each field of view and then stitched to obtain a full view of the entire section (Fig 1; Appendix Fig S1B–E).

A notable feature of the WGA-stained stitched images is the appearance of a distinct pattern of cells restricted to a particular geometric location (Fig EV1C). To quantitatively assess the cell patterns, we estimated the orientations at the scale of individual cardiomyocytes using a structure tensor method (Granlund & Knutsson, 1994; Materials and Methods; Figs 1 and EV2A). The fractional anisotropy colormap and the distribution of anisotropy values across the short-axis section illustrate the output of our automated structure tensor method for estimating the longest axis of a cardiomyocyte (Fig EV2A). To further validate our numerically obtained structure tensor orientation estimates, we compared them with the orientations of a selection of hand-segmented cardiomyocytes and found there to be a strong agreement between the two (mean difference 5.98° ± 2.3°; Figs 1 and EV2B).

Visualizing cell orientations using glyphs, streamlines, and angular projections

In order to visualize the estimated cardiomyocyte orientations, we adopted the use of glyphs and streamlines, that are often used to convey orientation estimates from DT-MRI data (Materials and Methods; Rohmer *et al*, 2007; Leemans, 2010). For visualizing the local orientation of each cardiomyocyte at the submicron voxel scale, we display every 12th voxel as an individual glyph (Fig 1; Movie EV1). We used bidirectional streamlines as a proxy for visualizing the aggregate cardiomyocyte orientations across the heart wall in both short- and long-axis sections. Our micron-scale 3D recovery of cardiomyocyte orientation across entire thick tissue sections of the heart represents a significant advance in recovering the geometric organization of cardiomyocytes since our reconstructions are at a much higher spatial resolution than that provided by previous studies (Gilbert *et al*, 2007; Materials and Methods; Fig 1; Movie EV1).

To quantitively analyze the estimated cardiomyocyte orientations, we plotted them using three angles: Theta (θ), Phi (Φ), and the Helix angle (α_H; Materials and Methods; Fig 2A). θ represents the acute angle between the projection of the estimated cell orientation (myofiber orientation) onto the short-axis (XY) plane and the X-axis. Φ represents the acute angle between the estimated cell orientation and the short-axis (XY) plane in the heart tissue. α_H is the angle between the projection of the myofiber onto the local tangent plane to the heart wall and the circumferential direction (Fig 2A). This corresponds to the helix angle, which is widely used in a body of work on macro-scale myofiber organization (Beg *et al*, 2004; Chen *et al*, 2005; Gilbert *et al*, 2007; Peyrat *et al*, 2007; Lombaert *et al*, 2012; Agger *et al*, 2020). Several studies have reported that α_H undergoes a smooth rotation along a penetration axis from epicardium to endocardium in the left ventricle (Beg *et al*, 2004; Chen *et al*, 2005; Gilbert *et al*, 2007). We therefore computed α_H using an

 © 2023 The Authors

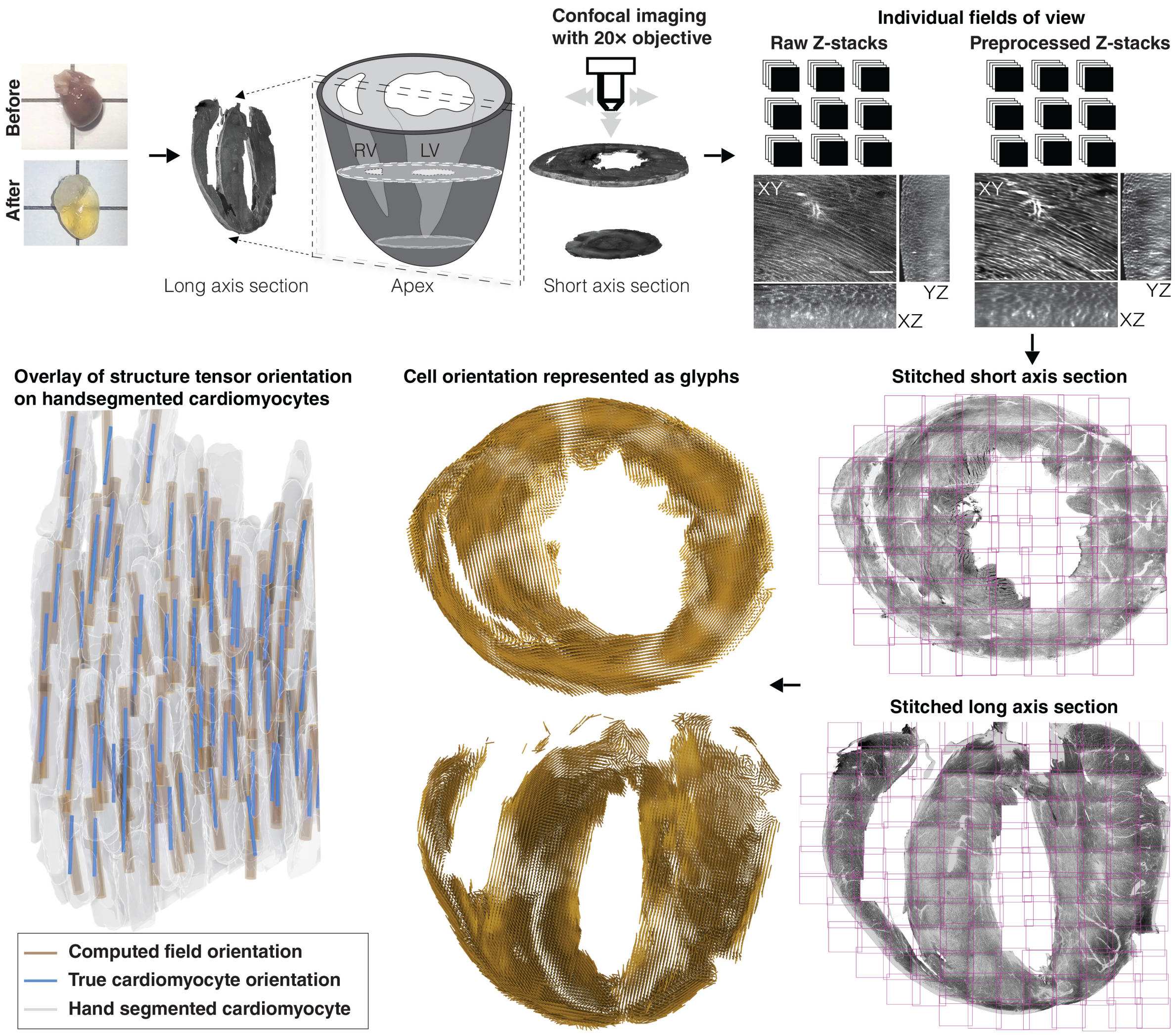

Figure 1. Overview of the heart tissue preparation, imaging and analysis pipeline.

Top panels from left to right: Representative photographs of a mouse heart before and after applying the CLARITY procedure (Tomer *et al*, 2014). An illustration of the cleared mouse heart sections analyzed in this study. The long-axis (LA) sections correspond to longitudinal dissections of the mouse heart through a transverse plane equivalent to the HLA-4C views of the AHA (or echocardiogram) nomenclature, revealing the right and left ventricular chambers. The short-axis (SA) sections are dissected at the midventricular plane, equivalent to PSAX-PML views of the AHA (or echocardiogram) nomenclature (Cerqueira *et al*, 2002). At least one mouse heart in this study provided four continuous sections, each being approximately 300 μm in thickness, for both the LA and SA analyses (Appendix Table S1; Materials and Methods). After sectioning and WGA staining, the tissue slices were imaged using confocal microcopy spanning the entire length, breadth, and width of the LA and SA sections (Materials and Methods). Using custom-built algorithms, the individual confocal stacks were preprocessed for contrast enhancement, denoised, and then stitched (Materials and Methods). *Bottom panels from right to left*: The imaging and preprocessing pipeline results at a 2-μm voxel isometric resolution of the entire SA and LA sections, up to a depth of about 300 μm in thickness. The estimated structure tensor vectors are visualized as glyphs represented as golden yellow lines for the entire SA and LA sections (Materials and Methods). A representative 3D view (bottom left) of the hand-segmented cardiomyocytes (gray) from the WGA stain, overlayed with the cell orientations estimated using the true myocyte orientation (purple) and the estimated field orientation (golden yellow) by the structure tensor method (Materials and Methods).

estimate of the radial penetration direction at each location in the heart wall (Materials and Methods; Figs 2A and EV2C).

Regimes of discrete cell orientations across ventricular walls

To demarcate the discrete cell arrangements across the ventricular walls (Fig 2B) and the boundaries between these different cell arrangements, we computed the Φ and θ angles defined in Fig 2A and visualized them using quantitative colormaps (Materials and Methods). The colormaps for the θ angle of a single plane in the short-axis (XY) view show that the cardiomyocytes are arranged in a radial direction following a smooth continuum (Fig 2C). Our findings at the micron scale are thus consistent with previous reports that heart wall myofibers wind around the ventricles in a circumferential manner (Spotnitz, 2000; Chen *et al*, 2005; Gilbert *et al*, 2007; Savadjiev *et al*, 2012; Poveda *et al*, 2013) (Movies EV2 and EV3).

© 2023 The Authors

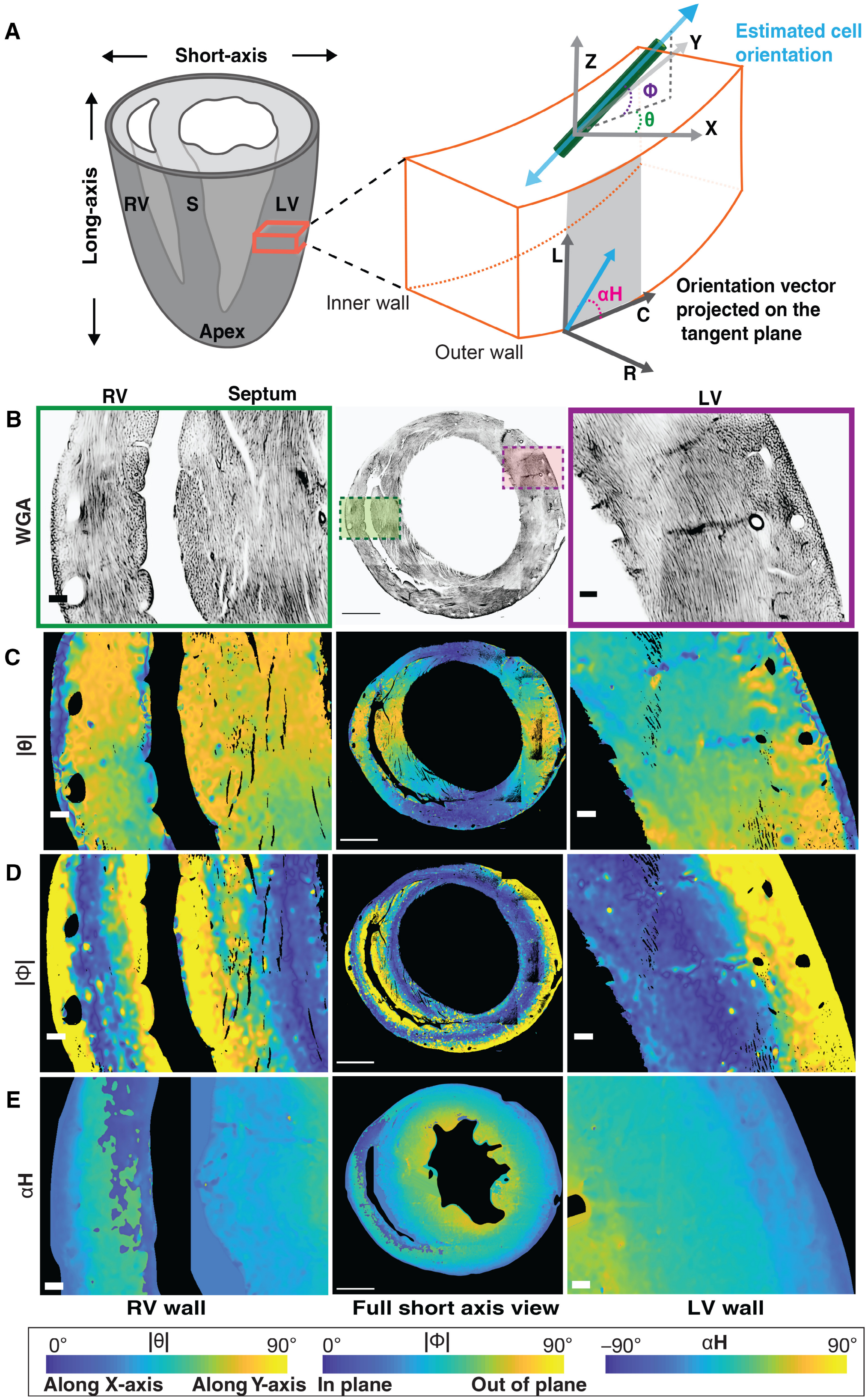

Figure 2.

 © 2023 The Authors

◀ **Figure 2. Cell orientation across a midventricular short-axis section of the heart.**

A An illustration of the angles measured to represent the cell orientations across the ventricular walls. The red box represents a magnified view of the ventricle wall, with the global axes and labels as indicated: C-Circumferential L-Longitudinal R-Radial. The green cylinder and the blue bidirectional arrow represent the cardiomyocyte and its long-axis, that is, the estimated cell orientation. Φ is the angle between the projection of the estimated cell orientation onto the XY plane and the estimated cell orientation. θ is the angle between the projection of the estimated cell orientation onto the XY plane and the X-axis direction. αH, the helix angle is the angle between the projection of the cell orientation onto the plane perpendicular to the transmural penetration direction and the circumferential direction.

B–E A magnified view of the right (green rectangle) and the left (magenta rectangle) ventricular regions for a full view of the WGA stain (middle). The |θ|, |Φ|, and αH angles for the mouse SA sections are shown using a parula colormap. The yellow tones for the |θ| angle represent cell orientation along the global Y-Axis while the blue tones represent cell orientation along the X-axis. For |Φ|, the blue and yellow tones represent cells with orientations aligned with the short-axis section, and orthogonal to it, respectively. The colormaps scale with the angles, as indicated. The scale bar for the full view images is 1,000 μm and for the magnified views is 100 μm.

In contrast to θ, the Φ angle reveals a discrete and significant change in cardiomyocyte orientation across the ventricular walls (Fig 2D). The out-of-plane cardiomyocytes are approximately perpendicular to the short-axis plane. This arrangement shown in yellowish tones in the colormaps forms a crescent shape along the outer walls of both ventricles and an orbicular shape in the inner walls surrounding both the chambers (Figs 2D, EV3A and EV4A–D). Taken together, the orientation reconstructions suggest that the out-of-plane cardiomyocytes at the edges of the ventricular walls have an orientation arrangement that is different from that of the well-established circumferential heart fibers (Movie EV3).

Sharp changes in myocyte orientation at ventricular wall boundaries

In a short-axis section, the Φ and α_H angles are related to cardiomyocyte orientation with respect to the viewing plane. α_H has been widely used to capture orientation changes along a transmural penetration from the outer to the inner heart wall (Beg *et al*, 2004; Chen *et al*, 2005; Gilbert *et al*, 2007). We therefore computed α_H at the micron scale using a ventricular outer boundary-based estimate of the radial penetration direction at each location in the left ventricular wall (Materials and Methods; Figs 2A and E and EV2C; Movie EV4). The 3D average α_H values for several wedge-shaped sectors were plotted as a function of transmural depth (Fig 3). The patterns of α_H values appear to be very similar at fixed angular distances with respect to the lateral region of short-axis view of the left ventricular wall (Fig 3). Sectors in the vicinity of the lateral region reveal a sharp drop of about 20° within the first 50 μm near the circumference of the outer wall (Figs 3 and EV2D). However, the sharp change in α_H from the outer wall boundary gradually disappears further from the lateral sectors toward inferior and anterior regions (Fig 3). Immediately after this sharp decline, α_H undergoes a gradual increase of about 180° for the remainder of the left ventricular wall, in a manner that is approximately linearly proportional to transmural depth (Fig 3) although the rate of change of α_H does vary (Fig EV2D and E). This latter smooth transition of α_H through the myocardium (middle wall) until the endocardium (inner wall) is consistent with earlier findings (Beg *et al*, 2004; Chen *et al*, 2005; Gilbert *et al*, 2007; Peyrat *et al*, 2007; Lombaert *et al*, 2012; Savadjiev *et al*, 2012). However, the initial sharp transition of an approximately 20° change in α_H, in the outer wall, is an entirely new discovery (Fig 3). We hypothesize that earlier studies lacked the required spatial resolution to identify the sharp change in α_H near the outer wall. To test this, we averaged the structure tensor, estimated at the micron scale, to a coarser spatial resolution, resulting in a pseudo low-resolution (~500 μm voxel resolution scale) estimate of cardiomyocyte orientation (Materials and Methods). This orientation estimate is qualitatively equivalent to the millimeter or submillimeter scale of previous DT-MRI studies. A comparison of our micron scale and pseudo low-resolution α_H angle estimates confirms the disappearance of the sharp change near the outermost layer, at the simulated coarser resolution (Fig 3). The long-axis cardiomyocyte layer we have reconstructed in the outer ventricular wall might have remained obscure in past studies (Beg *et al*, 2004; Chen *et al*, 2005; Gilbert *et al*, 2007; Peyrat *et al*, 2007; Lombaert *et al*, 2012; Savadjiev *et al*, 2012) due to its narrow width of only approximately 50 μm. As such, in our simulated submillimeter-scale coarse voxel analysis, it disappears in the orientation plots (Fig 3). In addition, a comparison of α_H between raw and preprocessed short-axis images shows that denoising improves the α_H angle estimates (Fig EV3B). Our reconstructions also demonstrate the presence of narrow longitudinal arrangements of cardiomyocytes in the inner ventricular chamber and septum walls, which have also been described in previous studies involving histological sections (Greenbaum *et al*, 1981; Fig 2E; Movie EV3).

Charting the continuity of the outer and inner wall long-axis myofibers

The analysis of short-axis sections allowed us to identify narrow bands of cardiomyocytes near the boundaries of the ventricular walls that are aligned to the long-axis direction of the heart (Fig 4A–D; Movies EV3 and EV4). To determine the spatial extent of this cardiomyocyte arrangement across the entire length of the ventricular walls, we turned our attention to the analysis of long-axis sections (HLA-4C). The WGA images and angular colormaps for the long-axis sections showed that the cardiomyocytes at the edges of the ventricular wall are aligned to be parallel to the section plane (Figs 5A and B and EV5; Movie EV5). The cardiomyocyte orientations at the ventricular edges are geometrically aligned with the long-axis direction (close to 0° with the section plane, or yellow tones in the colormaps, Figs 4 and 5 and EV5). This arrangement of cardiomyocytes extends all the way to the apex, where a complex geometry emerges. From our streamline visualizations, we infer that the bands of long-axis myofibers extend, intertwine, and continue into the opposite ends of the ventricular walls (Figs 5 and EV5; Movies EV6 and EV7).

In order to elucidate the complex geometry at the apex, we extended our sectioning toward the apex region along the short-axis

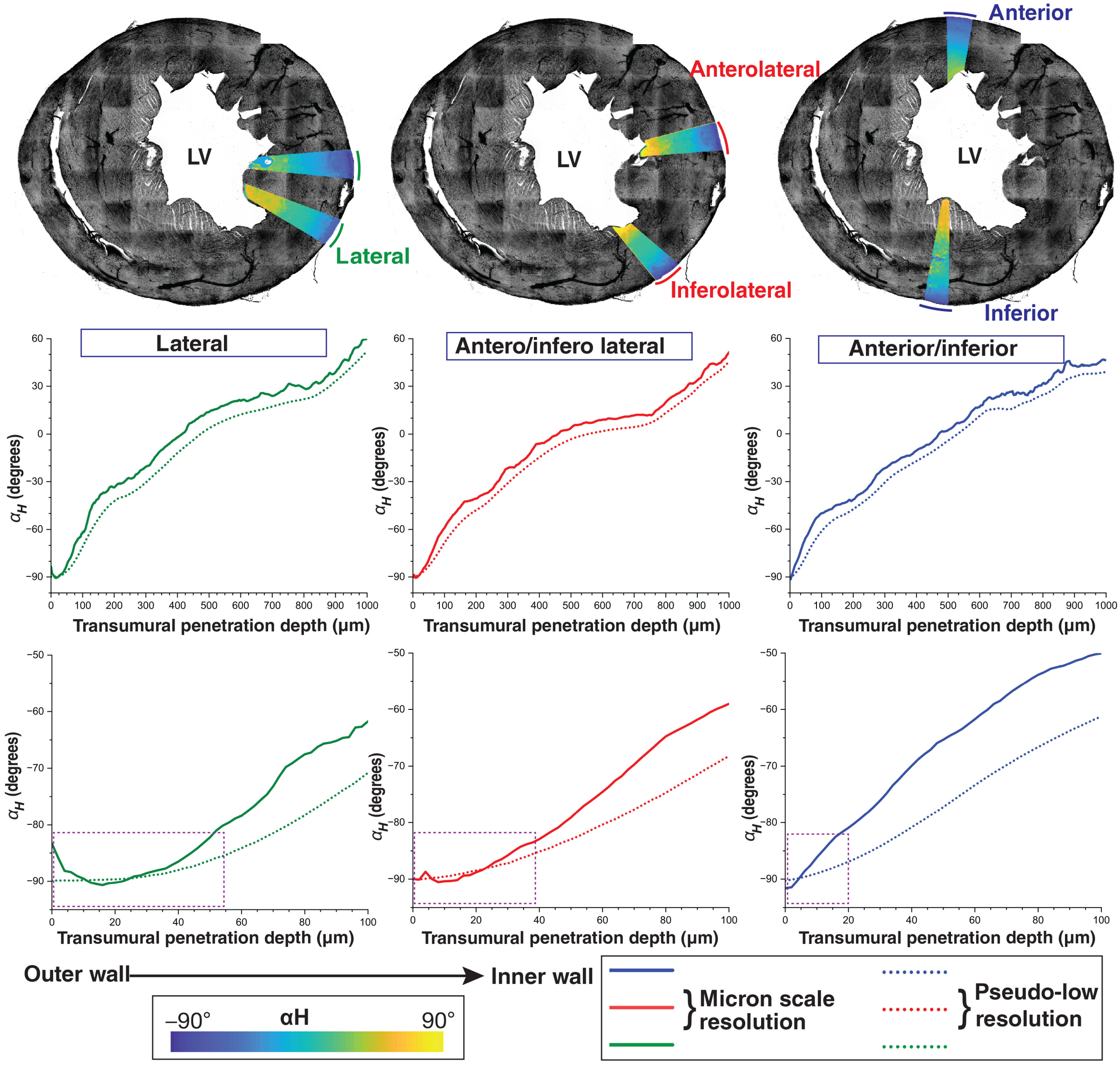

Figure 3. Helix angle plots for left ventricular wall sectors in a short-axis section.

The individual 10° wedge-shaped sectors are shown in three different groups; anterior/inferior, anterolateral, inferolateral, and lateral regions (top row panel). The wedge sector 3D average αH values were plotted and are highlighted as αH colormaps on a maximum intensity Z-projection of the WGA-stained short-axis section in grayscale. The 3D average αH values* are plotted as a function of the transmural penetration depth walls, depicted as distance in microns along the X-axis from the outer to the inner walls. The 3D average αH values from micron-scale and pseudo-resolution analyses of the selected sectors of the LV region from one representative data set of a short-axis section (SAS3) are shown in the middle row panel. A zoomed in version of the outer-ventricular region is provided in the bottom row panels. The region marked with a box in the bottom left panel represents the LV outer wall longitudinal cells. Data information: The values plotted here are derived from combining two sectors, spanning 10° in each, as illustrated.

plane (Fig 6; Materials and Methods). Here, the θ angle, depicting the component of cardiomyocyte orientation in the XY plane, shows a smooth transition, similar to that in the midventricular short-axis sections (Fig 2). The Φ angle reveals a confluence of three different bands of cardiomyocytes in the long-axis direction (Figs 5 and EV5). At the apex, these fiber systems turn upwards toward the base of the heart and then continue to the adjacent boundaries of the heart wall (Fig 6; Movie EV8). The long-axis cardiomyocyte arrangement in the left ventricular outer wall appears to smoothly continue into cardiomyocyte arrangements in the right ventricular outer and inner walls (Fig EV5). Thus, we observe a layer of cardiomyocytes that forms a continuum across the boundaries of the ventricular walls, running from base to apex (Appendix Fig S2A–C; Movies EV3, EV6 and EV7). This layer of cardiomyocyte arrangement when visualized using bidirectional streamlines portrays an entire long-axis myofiber system that is orthogonal to the established circumferential myofibers (Appendix Fig S2A–C; Movies EV3, EV6, and EV7).

 © 2023 The Authors

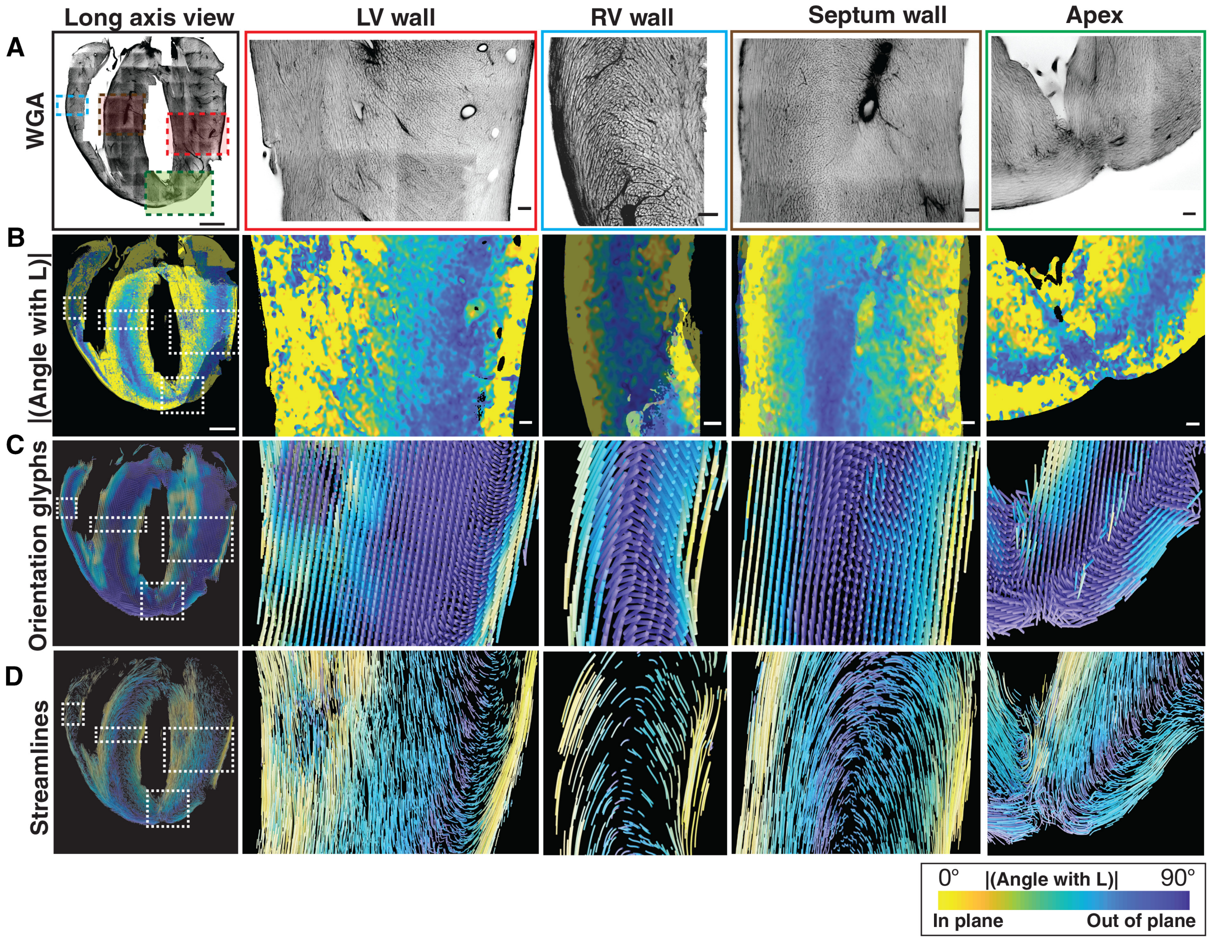

Figure 4. A detailed view of a long-axis section.

A WGA stain.

B Colormaps for the magnitude of the angle with the longitudinal axis (L).

C Estimated orientations as glyphs.

D Estimated orientations as streamlines.

Data information: The colors follow a parula colormap, where the blue and yellow colors indicate in and out of the short-axis plane cell orientations, respectively. Magnified views of the left ventricle (2nd column), right ventricle (3rd column), septum wall (4th column) and the apex region (5th column) are shown for the regions in panel (A). The color bar for the angle with the longitudinal axis is as indicated. The scale bar for the complete view is 1,000 μm and for the zoomed in regions is 100 μm.

In summary, by combining tissue clearing, light microscopy, and computer vision, our micron-scale recovery of cardiomyocyte orientation and myofiber geometry has revealed a long-axis fiber system in the outer wall of the heart (Fig 7). Both the outer wall long-axis system and the longitudinal fibers in the endocardium appear to be conserved features in the ventricular walls of four-chambered rodent hearts (Fig EV4A–D).

Discussion

Tissue clearing methods, together with three-dimensional imaging of biological samples, have revealed new cell types and their organization within organs (Tomer *et al*, 2014; Morales-Navarrete *et al*, 2019). Other studies have used tractography to visualize biological structures at various voxel resolutions including at the

Figure 5. Evidence for a long-axis fiber continuum from the analysis of a long-axis section.

A, B Estimated myofiber orientations are shown as glyphs and streamlines for the mouse LA sections as indicated. In each panel, a 3D visualization of the orientations is shown using either glyphs or streamlines, with a view obtained by rotation in a clockwise direction shown on the right (Materials and Methods). The colors follow a parula colormap, where the blue and yellow tones indicate cell orientations that are in and out of the short-axis plane, respectively. These visualizations reveal the continuity of cell orientations across the entire long-axis section, from base to apex.

© 2023 The Authors

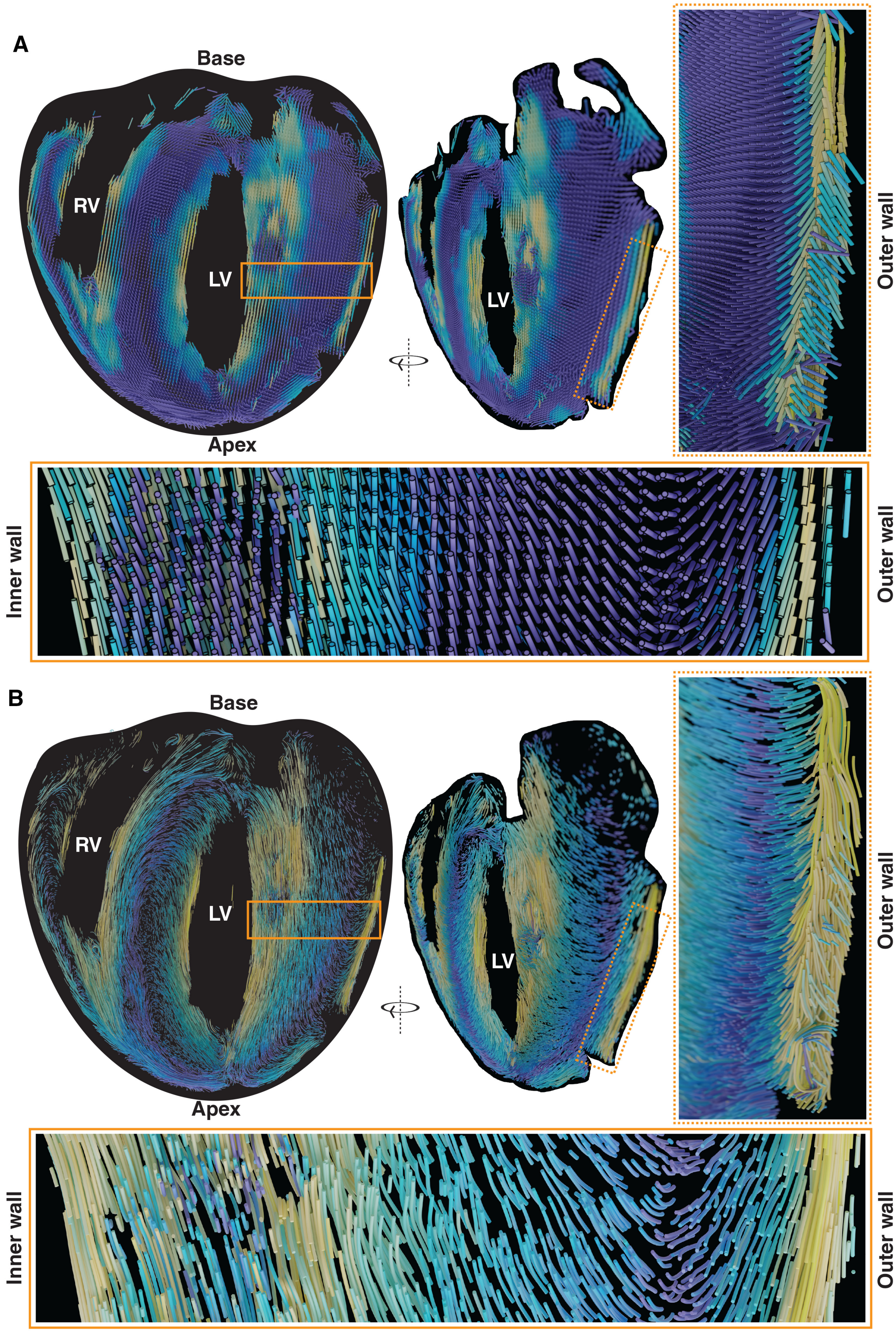

Figure 5.

millimeter, submillimeter, and micron scales (Beg *et al*, 2004; Chen *et al*, 2005; Gilbert *et al*, 2007; Savadjiev *et al*, 2012; Vinegoni *et al*, 2020). However, whole organ or tissue studies have seldom combined high-resolution three-dimensional volumetric imaging with quantitative assessment of cell orientations using computer vision methods. Here, we have presented an integrated pipeline

 © 2023 The Authors

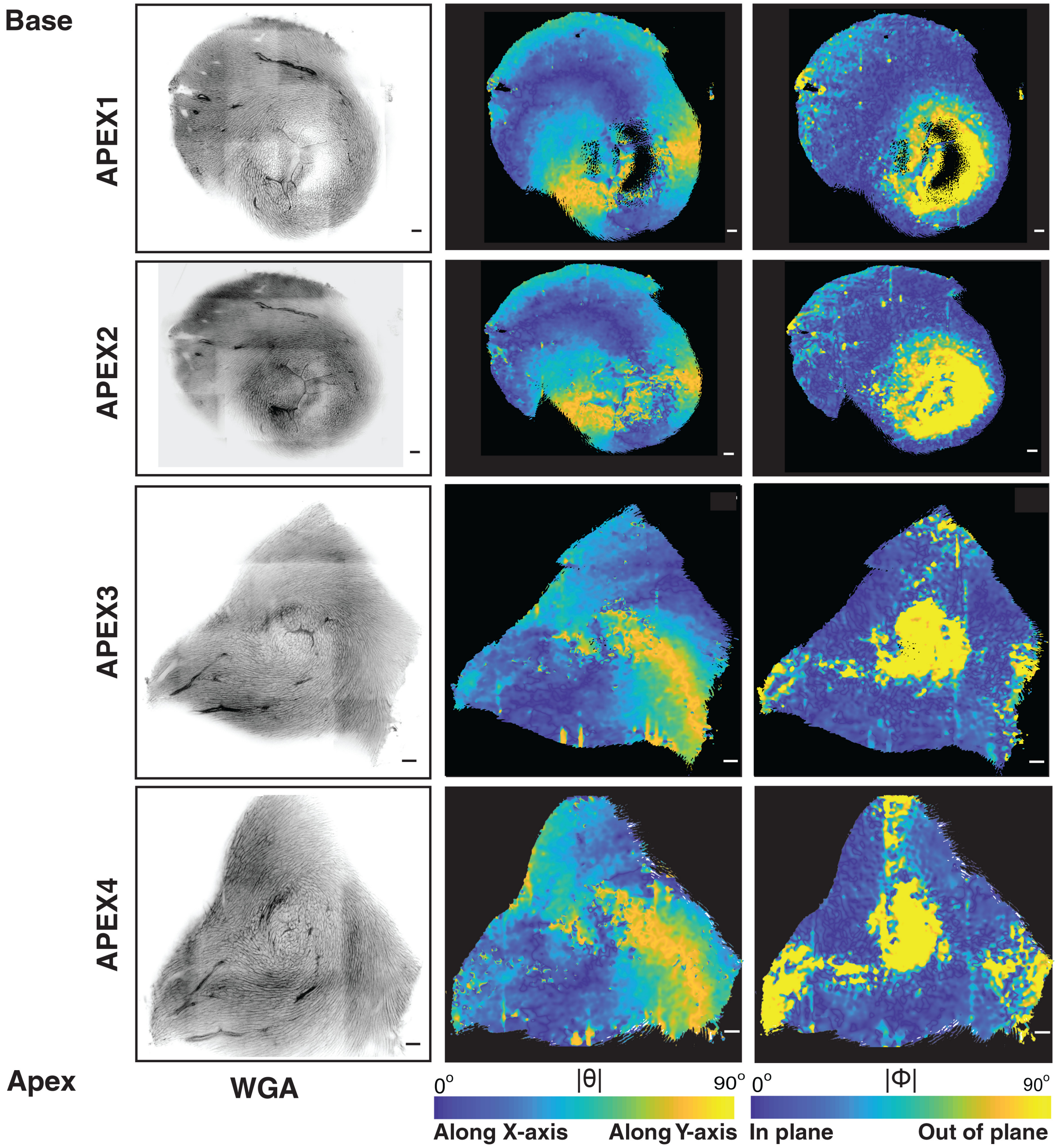

Figure 6. Analysis of a short-axis section from the apex region.

Representative Z-planes from four mouse heart apical serial sections are shown with WGA staining (left), and the |θ| (middle) and |Φ| (right) angles related to cell orientation using parula colormaps. The colormap scales are as indicated, with the scale bar being 100 μm.

to recover the micron-scale organization of densely packed cardiomyocytes in heart ventricular walls. A key advancement in our study is the integration of fluorescence-based micron-scale heart imaging with geometric image analysis of cardiomyocyte orientation and myofiber reconstruction methods from the microscopy images. This combination led to a resolution gain of about three orders of magnitude in scale (i.e., from millimeter to micron) for cell membrane orientation recovery, revealing a long-axis arrangement of cardiomyocytes, a system that has eluded discovery by previous lower resolution imaging methods (Gilbert *et al*, 2007). Other high-resolution imaging methods, such as synchrotron radiation imaging of rat hearts at 3 μm voxel resolution, have also revealed ultrastructural properties of heart tissue, including its cellular and extracellular features (Teh *et al*, 2017). However, due to a lack of cellular boundary information in such images, the geometric arrangements of cardiomyocytes cannot easily be recovered. Such methods have

© 2023 The Authors

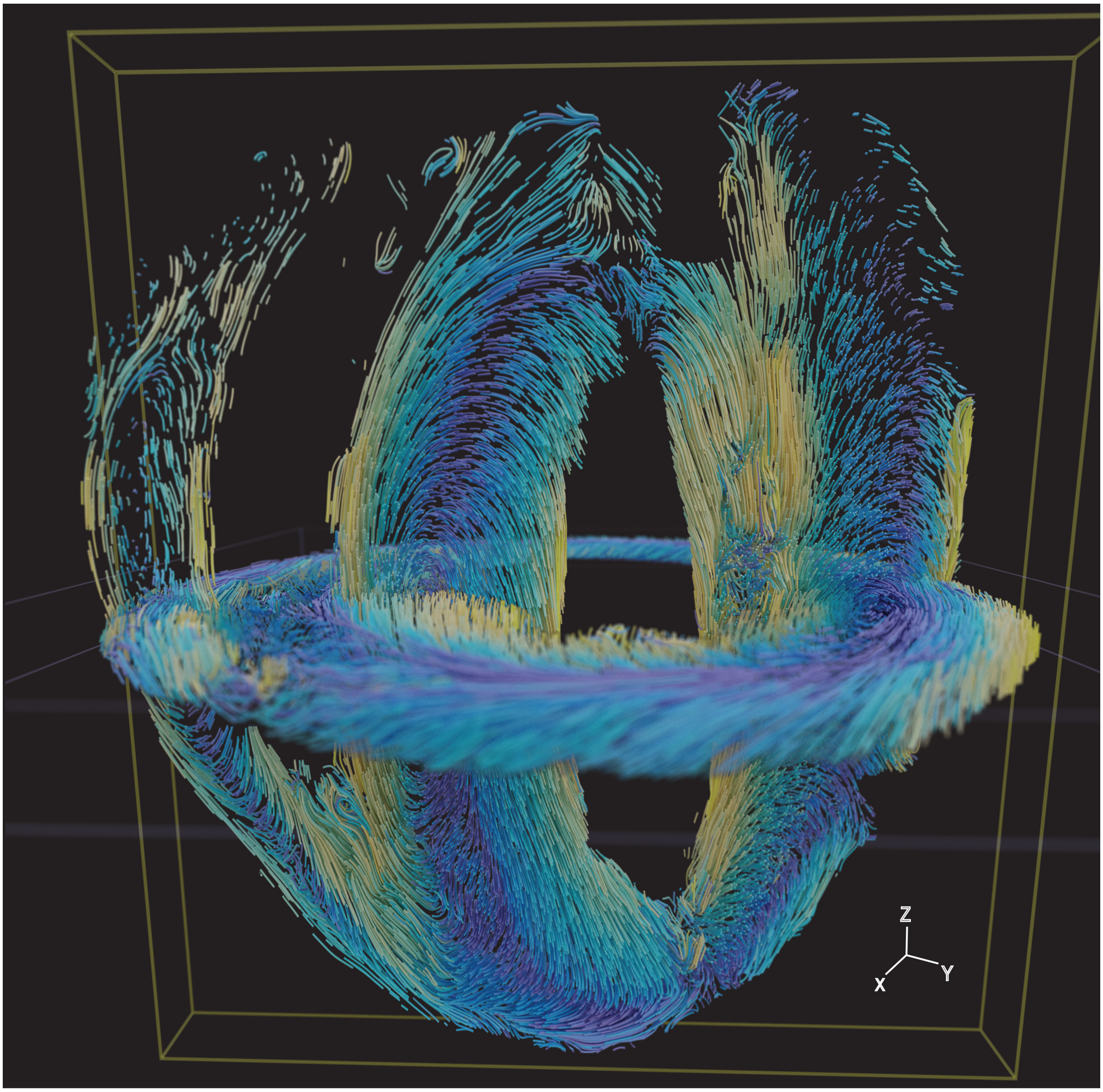

Figure 7. A model for orthogonal myofiber systems in heart ventricular walls.
The composite model is obtained by superposition of the reconstructions of the short-axis and long-axis sections from different mouse hearts. The structure tensor-based orientations are visualized as streamlines (Materials and Methods). The colors follow a parula colormap, where the blue and yellow tones indicate orientations in or out of the short-axis plane, respectively.

not reported sharp transitions in the helix angle in the anterolateral, inferolateral, and lateral regions of the LV wall. Reports of micron scale heart imaging focusing on collagen have observed a distinct epicardial pattern for the extracellular matrix (Pope *et al*, 2008), complementing our finding of long-axis cardiomyocyte arrangements. The integrated pipeline we have described can be applied to compare alterations in cellular and subcellular structures between healthy and diseased hearts. Our approach to estimate cell orientations at the micron scale can also be used to study the arrangement of ordered cell arrays in other biological systems, such as in tissues where cells have a dominant local orientation. The application of our methodology also provides an opportunity to generate and share digitized atlases of cell orientation field data of entire organs and tissues with the research community.

The vast majority of models for heart wall contraction and electrical wave conduction have only considered a helical arrangement of cardiomyocytes, rotating smoothly in a clockwise manner in a transmural penetration from outer wall to inner wall (Vetter *et al*, 2005; Young & Panfilov, 2010; Aumentado-Armstrong *et al*, 2018), consistent with models of fiber geometry based on DT-MRI (Beg *et al*, 2004; Chen *et al*, 2005; Gilbert *et al*, 2007; Bayer *et al*, 2012; Savadjiev *et al*, 2012). Our reconstruction of cardiomyocyte orientations at the micron scale and descriptions of arrangements of cardiomyocytes along the long-axis direction in the outer ventricular walls

© 2023 The Authors

of rodent hearts could advance present understanding of heart structure and function. During heart contraction, the ventricular walls undergo a wringing motion from the apex toward the base, accompanied by a slight shortening of the heart along its long-axis direction. Together, these motions generate an ejection force that empties the chambers. While the bulk of the contraction is carried out by the helical myofibers, which wrap around the chambers in a circumferential direction, the continuum of long-axis fibers might also play a role in long-axis shortening. A mathematical consideration of alternate geometries does in fact demonstrate the need for helical myofiber systems to optimize ejection fraction (Sallin, 1969). While the most recent tissue engineering papers (Chang *et al*, 2022) build on this and use a combination of helical and circular myofiber systems to simulate ventricular motion with wringing, the long-axis fiber systems in the outer walls are still conspicuously absent. Additionally, we have observed that the three bands of outer wall long-axis cardiomyocyte arrangements continue upwards to the base from the apex (Fig EV5).

Our reconstructions support a previous proposal, based on transmural penetrations of histological sections (Vetter *et al*, 2005), that a distinct long-axis epicardial myofiber system exists in the right ventricle and that it plays a vital role in electrical conduction, as demonstrated by simulations of electrical wave propagation. Our 3D reconstructions at the micron scale confirm the presence of such a system, but further reveal a much more prominent system in the outer wall of the left ventricle (Figs 2, 4, and 5), which might play a complementary role in facilitating electrical conduction in the heart walls. The geometry of the outer wall long-axis cardiomyocyte arrangements is quite complex at the apex, resembling a confluence of three systems (Fig 6). It appears to not be smooth at the apex, as a multitude of models derived from DT-MRI have predicted. Our reconstruction of a prominent geometrically distinct long-axis epicardial arrangement of cardiomyocytes is further supported by observations that a thin epicardial cell layer orthogonal to that in the midwall exists in humans (Drouin *et al*, 1995) and that in the rabbit and the pig (Scollan *et al*, 1998; Vetter *et al*, 2005), sharp changes of action potential coincide with changes in cell geometry and orientation in the outer wall (Yan *et al*, 1998). In a recent article using millimeter scale diffusion imaging of pig hearts, Wilson *et al* report a nonuniform variation of α_H, in different regimes of the heart wall (Wilson *et al*, 2023).

The electrical signals from the sinoatrial node propagate to the atrioventricular node to cause the ventricles to pump blood, via a network of Purkinje fibers (Sedmera & Gourdie, 2014; Romero *et al*, 2016). Analyses have shown that a helical continuum of fibers, together with their associated transmural turning from the outer wall to the inner wall, can explain the point-to-point time to arrival conduction wave propagation maps in the heart wall, under a model of anisotropic diffusion (Young & Panfilov, 2010). Moreover, transmural turning has been shown to play a role in minimizing diffusion bias and mitigating the potentially harmful effects of stochastic propagation (Aumentado-Armstrong *et al*, 2018). However, how the timing of the contraction is controlled at any short-axis section is an open question. We hypothesize that the propagation of the conduction wave through the heart wall along the transmural direction could be mediated by the helical continuum of myofibers (Young & Panfilov, 2010; Aumentado-Armstrong *et al*, 2018) and that the long-axis outer wall fibers facilitate the propagation of the signal from the apex to the base in a longitudinal direction. This would then explain the overall timing behavior observed in the contraction of the ventricles and the need for such geometrically distinct orthogonal fiber systems.

Orthogonal fiber systems are, in fact, not restricted to mammalian hearts. Similar fiber organizations have been reported in other organs and entire animal body plans (Diamant, 1989; Mittal, 2013; Scimone *et al*, 2017). At the organ level, the coexistence of a circumferential and longitudinal fiber system is known to be important for the smooth muscle peristaltic motions seen in esophageal and intestinal tubes (Diamant, 1989; Mittal, 2013). In planarians, in addition to the circumferential and longitudinal muscle fibers, a diagonal fiber system has been reported (Scimone *et al*, 2017). However, a crucial difference between the heart and these other systems is that in the latter, the orthogonal arrays occupy an equal volume (Diamant, 1989; Mittal, 2013; Scimone *et al*, 2017). In contrast, our reconstructions show that the longitudinal cardiomyocyte arrangements account for only a small fraction of the cardiomyocytes in the heart wall, suggesting that they have highly specialized roles. It remains to be seen whether perturbations of the long-axis arrangements are associated with heart diseases, including cardiomyopathies or electrical conduction disorders. The embryonic stage of heart development at which the outer wall long-axis cardiomyocyte array begins to form is also not known.

Hypertrophic (HCM) and dilated (DCM) cardiomyopathies are clinically evaluated as a thickening or thinning of the left ventricular and septal walls, respectively (Marian & Braunwald, 2017). At the histological level, excess fibrosis and fiber disarray are frequently reported to be associated with these diseased states. However, a correlative evaluation of enlarged or diminished ventricular wall dimension and fiber architecture in a volumetric sense at the scale of individual cardiomyocytes is still missing. A spectrum of HCM clinical phenotypes has also been observed from echocardiogram studies among different patients (Marian & Braunwald, 2017). However, disruptions in myofiber organization caused by HCM have so far been described at a gross morphological level only using only DT-MRI studies (Chen *et al*, 2003; Schmitt *et al*, 2009; Garcia-Canadilla *et al*, 2019). The myofiber geometry at the micron scale reconstructed by our methods lays a foundation for analysis at a finer scale, to discriminate between symmetrical and asymmetrical fiber disruption, and more generally for the study of the association between pathological heart conditions and cardiomyocyte arrangements.

Materials and Methods

Experimental model and subject details

The animal preparation and image work were conducted at the NCBS/inStem Animal Care and Resource Centre and was approved by the inStem Institutional Animal Ethics Committee, following the norms specified by the Committee (Approval Numbers: INS-IAE-2018/03(E), INS-IAE-2020/12(N), NCBS-IAE-2012/05 (R1) SC-5/2009 SC-5/2012, with CPCSEA registration no. 109/GO/bc/99/CPCSEA) for control and supervision of experiments on animals (Government of India). We used the C57BL/6 strain of female mice and the Wistar strain of male rat. These were housed in the institute

animal house and were maintained in a 12-h light/dark cycle. The animals used in our studies were 6–8 weeks in age.

A flow chart illustrating the steps in the preparation and imaging of biological tissue samples is shown in Appendix Fig S3.

Tissue clearing

The mouse and rat hearts were collected from wild-type female C57BL/6 and Wistar strains, respectively. All the heart samples were perfused during the collection with heparinized 1× PBS to remove blood clots, followed by 4% paraformaldehyde (PFA). The fixed hearts were stored at 4°C until further use. To clear the heart tissue, we applied the CLARITY method (Tomer *et al*, 2014) with the following modifications: The fixed mouse hearts were transferred to a hydrogel monomer solution (PBS, 4% acrylamide, 4% PFA, 0.5% Bisacrylamide, and 0.25% photo-initiator 2, 20-Azobis [2-(2-imidazolin-2-yl) propane] dihydrochloride (VA-044, Wako Chemicals USA) for 7 days. For initiating the hydrogel hybridization and polymerization the processed heart tissues were incubated for 3 h at 37°C. After polymerization, the excess gel material was removed. The tissue was transferred to 50 -ml tubes and washed 3 times with PBS, and then incubated with a clearing buffer (8% SDS and 4% boric acid in 1X PBS (pH 8.5) for 20–30 days at 37°C, in a shaking incubator at 180 rpm, with the exchange of a new clearing buffer every week. This CLARITY-based approach applied to heart tissue samples resulted in a transparent tissue (Figs 1 and EV1A).

Sample preparation and imaging

The cleared mouse hearts and uncleared mouse and rat hearts were subjected to short- and long-axis sectioning using a Compresstome VF-300, as illustrated in Fig EV1B. The sectioned heart tissues were processed for staining by washing them with PBS 3 times, followed by the application of PBST (PBS + 1% Triton X-100) for 24 h at 37°C. Subsequently, the heart sections were incubated in 150 μg/ml of Alexa Fluor™ 633 conjugated wheat germ agglutinin (WGA, W21404, Thermo Fisher) for 24 h and washed with PBS, 3 times, for 10 min each time. The WGA stained cleared and uncleared heart tissues were then transferred into RIMS imaging media (88% Histodenz Sigma D2158 in 20 mM Phosphate buffer pH 7.5). The heart tissues were then mounted with fresh RIMS, sandwiched between two coverslips using 500 μm spacers (IS002, SUNjin Lab, Taiwan). The confocal images were acquired using an Olympus FV3000 microscope with an Olympus PlanApo 1.25× and Olympus UCPLFN 20X CorrM32 85 mm scale air objective (NA = 0.73). For each section, using the 1.25× objective, a lower magnification image encompassing the whole area of the section was obtained, which was then used to map the fields of view using the Olympus fluoView™ software. Micron-scale imaging was carried out using a 20× objective, with each field of view covering 320 × 320 pixels with an isometric voxel size of 1.98 μm^3. For the cleared and uncleared heart tissues, a maximum depth of 300 and 50 μm of image stacks was acquired, respectively. The samples were excited using a 640 nm laser line and emission was detected over a range of 650–670 nm using high sensitivity spectral detectors (gallium arsenide phosphide photomultiplier tube).

A flow chart illustrating the steps in the computational pipeline is shown in Appendix Fig S4.

Preprocessing of heart tissue image stacks

At the deeper end of the image stacks, we observed a poor signal to noise ratio. In order to improve the fluorescence signal, we performed deconvolution and denoising using custom-built algorithms. For the deconvolution, we corrected the nonideal point spread function (PSF), shown in Appendix Fig S5, using the iterative Richardson–Lucy deconvolution method (Dey *et al*, 2006) with Total variation regularization. The deconvolved image stacks were then subjected to unsupervised denoising using a dictionary learning method (Mairal *et al*, 2014). The dictionary was extracted from the shallow layers of image stacks under the assumption that the deeper and shallow layers contained common substructure elements of cells. This set of learned dictionary patches (a sparse 256 element 2D dictionary of patches of size 16 × 16, shown in Appendix Fig S6) from cardiac tissue was used to computationally clear the noise and improve the visibility of cell structures at the deeper end of the stacks. A separate dictionary file was generated for each individual field of view to prevent structures from one region from influencing the reconstruction in other regions of the heart tissue sample. The deconvolved and denoised image stacks were stitched together to yield a micron-scale complete short- and long-axis section, with a depth of about 300 μm. To achieve this, during imaging, each individual field of view was set to have at least a 25% overlap with its neighboring fields of view. The overlapping fields of view were tiled using phase correlation between adjacent fields of view in the Fourier domain (Zukić *et al*, 2021). The tiled reconstructions were globally aligned with American Heart Association (AHA) sectors in a manual fashion, using features including capillary vessels and papillary muscle placement and orientation.

Cell orientation estimation using a structure tensor

The intensity of the WGA stain, which is absorbed by the cell membranes of the myocytes, was used to estimate myocyte orientation. At each voxel in the deconvolved and denoised image stack, we computed the structure tensor (Knutsson *et al*, 2011). We then associated to each voxel the orientation of the direction in which the intensity varied the least, capturing the long-axis orientation of myocytes, by selecting the eigenvector of the structure tensor corresponding to the eigenvalue with smallest magnitude. The mapping of myocyte orientation to coarser spatial scales was carried out by element-wise averaging of local structure tensors, followed by eigenvector decomposition of the smoothed tensors. To validate the structure tensor-based estimates of cell orientations, we compared them with the ground truth orientations of several hand-segmented myocytes. The cell orientation was then interpreted in terms of its projection in the short-axis plane θ, the component out of the short-axis plane Φ, and the angle α_H between its projection onto the heart wall tangent plane and the long-axis direction (Fig 2A).

Colormaps, glyphs, and streamline generation

The orientation field was represented by a 3-dimensional array of rotated cylinders, which we refer to as "orientation glyphs." The input vector field was approximated by a 3-dimensional grid of equally spaced vertices, down-sampled such that the number of vertices was not larger than 75,000. At each vertex of the down-sampled grid, a

 © 2023 The Authors

cylinder primitive shape was created and rotated proportionally to the components of the vector field at the vertex position. A parula colormap was applied to the cylinders in a manner that was proportional to the Φ angle, using the arc cosine of the absolute value of the component of the vector field in the Z-axis direction. Bidirectional streamlines were represented as curves extruded from polylines, whose points were computed as follows: A set of up to 25,000 points were selected from a random sample of voxels from the vector field, and each sample voxel location was the initial point for a streamline. Since the vector field represents the orientation of the tissue, each starting point initialized both positive and negative streamlines, each of which was grown by iteratively adding new points along the polyline. At each new position, the value of the field at that voxel acted to determine the position of the following point along the polyline. A parula colormap was applied to the streamlines in proportion to the Φ angle at the starting point within the vector field. To avoid artifacts where the streamlines extended beyond imaged data, a binary mask of the tissue volume with the same dimensions as the vector field served as a boundary condition.

Data availability

Sample datasets and customized implementations of algorithms used for the current study are available in the GitHub repository: [https://gitfront.io/r/myoarch/fZmkh5iHEJae/myofibrometry/]. A detailed account of the methods is available as Appendix.

Expanded View for this article is available online.

Acknowledgments
We thank Drs. Keith Murai, Carsten Janke, Ramkumar Sambasivan, James Spudich, and Logan Walsh and members of the Sirajuddin lab for their insightful comments on the manuscript. We are grateful to the Central Imaging and Flow Facility (CIFF) and the Animal Care and Resource Center (ACRC) at the Bangalore Life Science Cluster, India. The ACRC was partially supported by the National Mouse Research Resource grant (BT/PR5981/MED/31/181/2012;2013-2016 & 102/IFD/SAN/5003/2017-2018) from the Department of Biotechnology, India. MS acknowledges funding support from inStem core grants from the Department of Biotechnology, India, a DBT/Wellcome Trust India Alliance Intermediate Fellowship (IA/I/14/2/501533), an EMBO Young Investigator Programme award, CEFIPRA (5703-1) from the Department of Science and Technology, a SERB-EMR grant (CRG/2019/003246) and a DBT-BIRAC (BT/PR40389/COT/142/6/2020) grant. PSD is supported by the DBT/Wellcome Trust India Alliance Intermediate Fellowship (IA/I/16/1/502367), the Rajiv Gandhi University of Health Sciences (RGUHS), Scientist Development Grant (15SDG23250005) from the American Heart Association (AHA), a Department of Science and Technology Grant (DST/CRG/2019/005401) and inStem core funding. We thank the Perundurai lab for providing the mouse heart microCT 3D image shown in the movies and for their help in obtaining heart tissue samples. KS is grateful to the Natural Sciences and Engineering Research Council of Canada (NSERC: RGPIN-2018-06323 & RGPAS-522584-18) and to the Fonds de Recherche du Québec Nature et Technologies (FRQNT: 256314/201) for research funding. DD is supported by a CSIR-SRF.

Author contributions
Drisya Dileep: Resources; data curation; software; formal analysis; validation; investigation; visualization; methodology; writing – original draft; project administration; writing – review and editing. **Tabish A Syed:** Resources; data curation; software; formal analysis; validation; investigation; visualization; methodology; writing – original draft; project administration; writing – review and editing. **Tyler FW Sloan:** Software; formal analysis; visualization; methodology. **Perundurai S Dhandapany:** Resources; funding acquisition; methodology. **Kaleem Siddiqi:** Conceptualization; supervision; funding acquisition; investigation; writing – original draft; project administration; writing – review and editing. **Minhajuddin Sirajuddin:** Conceptualization; supervision; funding acquisition; investigation; writing – original draft; project administration; writing – review and editing.

Disclosure and competing interests statement
DD, TAS, PSD, KS, and MS declare no competing interests. TFWS is the sole proprietor of Quorumetrix Studio and provided custom scientific data processing and 3D visualization services used in this study.

References

Agger P, Omann C, Laustsen C, Stephenson RS, Anderson RH (2020) Anatomically correct assessment of the orientation of the cardiomyocytes using diffusion tensor imaging. *NMR Biomed* 33: e4205

Anderson RH, Smerup M, Sanchez-Quintana D, Loukas M, Lunkenheimer PP (2009) The three-dimensional arrangement of the myocytes in the ventricular walls. *Clin Anat* 22: 64–76

Aumentado-Armstrong T, Kadivar A, Savadjiev P, Zucker SW, Siddiqi K (2018) Conduction in the heart wall: helicoidal fibers minimize diffusion bias. *Sci Rep* 8: 7165

Bayer JD, Blake RC, Plank G, Trayanova NA (2012) A novel rule-based algorithm for assigning myocardial fiber orientation to computational heart models. *Ann Biomed Eng* 40: 2243–2254

Beg MF, Helm PA, McVeigh E, Miller MI, Winslow RL (2004) Computational cardiac anatomy using MRI. *Magn Reson Med* 52: 1167–1174

Cerqueira MD, Weissman NJ, Dilsizian V, Jacobs AK, Kaul S, Laskey WK, Pennell DJ, Rumberger JA, Ryan T, Verani MS *et al* (2002) Standardized myocardial segmentation and nomenclature for tomographic imaging of the heart. A statement for healthcare professionals from the Cardiac Imaging Committee of the Council on Clinical Cardiology of the American Heart Association. *Circulation* 105: 539–542

Chang H, Liu Q, Zimmerman JF, Lee KY, Jin Q, Peters MM, Rosnach M, Choi S, Kim SL, Ardoña HAM *et al* (2022) Recreating the heart's helical structure-function relationship with focused rotary jet spinning. *Science* 377: 180–185

Chen J, Song S-K, Liu W, McLean M, Allen JS, Tan J, Wickline SA, Yu X (2003) Remodeling of cardiac fiber structure after infarction in rats quantified with diffusion tensor MRI. *Am J Physiol Heart Circ Physiol* 285: H946–H954

Chen J, Liu W, Zhang H, Lacy L, Yang X, Song S-K, Wickline SA, Yu X (2005) Regional ventricular wall thickening reflects changes in cardiac fiber and sheet structure during contraction: quantification with diffusion tensor MRI. *Am J Physiol Heart Circ Physiol* 289: H1898–H1907

Corno AF, Kocica MJ, Torrent-Guasp F (2006) The helical ventricular myocardial band of Torrent-Guasp: potential implications in congenital heart defects. *Eur J Cardiothorac Surg* 29: S61–S68

Cretoiu D, Pavelescu L, Duica F, Radu M, Suciu N, Cretoiu SM (2018) Myofibers. In *Muscle atrophy*, Xiao J (ed), pp 23–46. Singapore: Springer

von Deuster C, Sammut E, Asner L, Nordsletten D, Lamata P, Stoeck CT, Kozerke S, Razavi R (2016) Studying dynamic myofiber aggregate

© 2023 The Authors

reorientation in dilated cardiomyopathy using in vivo magnetic resonance diffusion tensor imaging. *Circ Cardiovasc Imaging* 9: e005018

Dey N, Blanc-Feraud L, Zimmer C, Roux P, Kam Z, Olivo-Marin J-C, Zerubia J (2006) Richardson–Lucy algorithm with total variation regularization for 3D confocal microscope deconvolution. *Microsc Res Tech* 69: 260–266

Diamant NE (1989) Physiology of esophageal motor function. *Gastroenterol Clin North Am* 18: 179–194

Drouin E, Charpentier F, Gauthier C, Laurent K, Le Marec H (1995) Electrophysiologic characteristics of cells spanning the left ventricular wall of human heart: evidence for presence of M cells. *J Am Coll Cardiol* 26: 185–192

Garcia-Canadilla P, Cook AC, Mohun TJ, Oji O, Schlossarek S, Carrier L, McKenna WJ, Moon JC, Captur G (2019) Myoarchitectural disarray of hypertrophic cardiomyopathy begins pre-birth. *J Anat* 235: 962–976

Geerts-Ossevoort L, Bovendeerd P, Prinzen F, Arts T, Nicolay K (2001) Myofiber orientation in the normal and infarcted heart, assessed with MR-diffusion tensor imaging. *Comput Cardiol* 28: 621–624

Gilbert SH, Benson AP, Li P, Holden AV (2007) Regional localisation of left ventricular sheet structure: integration with current models of cardiac fibre, sheet and band structure. *Eur J Cardiothorac Surg* 32: 231–249

Granlund GH, Knutsson H (1994) *Signal processing for computer vision*. Berlin, Germany: Springer Science & Business Media

Greenbaum RA, Ho SY, Gibson DG, Becker AE, Anderson RH (1981) Left ventricular fibre architecture in man. *Br Heart J* 45: 248–263

Helm P, Beg MF, Miller MI, Winslow RL (2005) Measuring and mapping cardiac fiber and laminar architecture using diffusion tensor MR imaging. *Ann N Y Acad Sci* 1047: 296–307

Horowitz A, Perl M, Sideman S (1993) Geodesics as a mechanically optimal fiber geometry for the left ventricle. *Basic Res Cardiol* 88: 67–74

Jouk PS, Usson Y, Michalowicz G, Grossi L (2000) Three-dimensional cartography of the pattern of the myofibres in the second trimester fetal human heart. *Anat Embryol (Berl)* 202: 103–118

Knutsson H, Westin C-F, Andersson M (2011) Representing local structure using tensors II. In *Image analysis*, Heyden A, Kahl F (eds), pp 545–556. Berlin, Heidelberg: Springer

Leemans A (2010) Visualization of diffusion MRI data. In *Diffusion MRI: theory, methods, and applications*, Jones DK (ed), pp 354–380. Oxford, UK: Oxford University Press

LeGrice IJ, Smaill BH, Chai LZ, Edgar SG, Gavin JB, Hunter PJ (1995) Laminar structure of the heart: ventricular myocyte arrangement and connective tissue architecture in the dog. *Am J Physiol* 269: H571–H582

Libby P, Bonow R, Mann D, Tomaselli G, Bhatt D, Solomon S, Braunwald E (2018) *Braunwald's heart disease: a textbook of cardiovascular medicine, 2-volume set 11th edition*. Philadelphia, PA: Elsevier Science

Lombaert H, Peyrat J-M, Croisille P, Rapacchi S, Fanton L, Cheriet F, Clarysse P, Magnin I, Delingette H, Ayache N (2012) Human atlas of the cardiac fiber architecture: study on a healthy population. *IEEE Transactions on Medical Imaging* 31: 1436–1447

Mairal J, Bach F, Ponce J (2014) *Sparse modeling for image and vision processing*. Hanover, MA: Now Publishers Inc.

Marian AJ, Braunwald E (2017) Hypertrophic Cardiomyopathy. *Circ Res* 121: 749–770

Merz SF, Korste S, Bornemann L, Michel L, Stock P, Squire A, Soun C, Engel DR, Detzer J, Lörchner H *et al* (2019) Contemporaneous 3D characterization of acute and chronic myocardial I/R injury and response. *Nat Commun* 10: 2312

Mittal RK (2013) Longitudinal muscle of the esophagus: its role in esophageal health and disease. *Curr Opin Gastroenterol* 29: 421–430

Morales-Navarrete H, Nonaka H, Scholich A, Segovia-Miranda F, de Back W, Meyer K, Bogorad RL, Koteliansky V, Brusch L, Kalaidzidis Y *et al* (2019) Liquid-crystal organization of liver tissue. *eLife* 8: e44860

Nehrhoff I, Ripoll J, Samaniego R, Desco M, Gómez-Gaviro MV (2017) Looking inside the heart: a see-through view of the vascular tree. *Biomed Opt Express* 8: 3110–3118

Perbellini F, Liu AKL, Watson SA, Bardi I, Rothery SM, Terracciano CM (2017) Free-of-Acrylamide SDS-based Tissue Clearing (FASTClear) for three dimensional visualization of myocardial tissue. *Sci Rep* 7: 5188

Peskin CS (1989) Fiber architecture of the left ventricular wall: an asymptotic analysis. *Commun Pure Appl Math* 42: 79–113

Peyrat J-M, Sermesant M, Pennec X, Delingette H, Xu C, McVeigh ER, Ayache N (2007) A computational framework for the statistical analysis of cardiac diffusion tensors: application to a small database of canine hearts. *IEEE Trans Med Imaging* 26: 1500–1514

Pope AJ, Sands GB, Smaill BH, LeGrice IJ (2008) Three-dimensional transmural organization of perimysial collagen in the heart. *Am J Physiol Heart Circ Physiol* 295: H1243–H1252

Poveda F, Gil D, Martí E, Andaluz A, Ballester M, Carreras F (2013) Helical structure of the cardiac ventricular anatomy assessed by diffusion tensor magnetic resonance imaging with multiresolution tractography. *Rev Esp Cardiol (Engl Ed)* 66: 782–790

Rohmer D, Sitek A, Gullberg GT (2007) Reconstruction and visualization of fiber and laminar structure in the normal human heart from ex vivo diffusion tensor magnetic resonance imaging (DTMRI) data. *Invest Radiol* 42: 777–789

Romero D, Camara O, Sachse F, Sebastian R (2016) Analysis of microstructure of the cardiac conduction system based on three-dimensional confocal microscopy. *PLoS One* 11: e0164093

Rushmer RF, Crystal DK, Wagner C (1953) The functional anatomy of ventricular contraction. *Circ Res* 1: 162–170

Sallin EA (1969) Fiber orientation and ejection fraction in the human left ventricle. *Biophys J* 9: 954–964

Savadjiev P, Strijkers GJ, Bakermans AJ, Piuze E, Zucker SW, Siddiqi K (2012) Heart wall myofibers are arranged in minimal surfaces to optimize organ function. *Proc Natl Acad Sci USA* 109: 9248–9253

Schmitt B, Fedarava K, Falkenberg J, Rothaus K, Bodhey NK, Reischauer C, Kozerke S, Schnackenburg B, Westermann D, Lunkenheimer PP *et al* (2009) Three-dimensional alignment of the aggregated myocytes in the normal and hypertrophic murine heart. *J Appl Physiol* 107: 921–927

Scimone ML, Cote LE, Reddien PW (2017) Orthogonal muscle fibres have different instructive roles in planarian regeneration. *Nature* 551: 623–628

Scollan DF, Holmes A, Winslow R, Forder J (1998) Histological validation of myocardial microstructure obtained from diffusion tensor magnetic resonance imaging. *Am J Physiol* 275: H2308–H2318

Sedmera D, Gourdie RG (2014) Why do we have Purkinje fibers deep in our heart? *Physiol Res* 63: S9–S18

Seidel T, Edelmann J-C, Sachse FB (2016) Analyzing remodeling of cardiac tissue: a comprehensive approach based on confocal microscopy and 3D reconstructions. *Ann Biomed Eng* 44: 1436–1448

Sivaguru M, Fried G, Sivaguru BS, Sivaguru VA, Lu X, Choi KH, Saif MTA, Lin B, Sadayappan S (2015) Cardiac muscle organization revealed in 3-D by imaging whole-mount mouse hearts using two-photon fluorescence and confocal microscopy. *Biotechniques* 59: 295–308

Spotnitz HM (2000) Macro design, structure, and mechanics of the left ventricle. *J Thorac Cardiovasc Surg* 119: 1053–1077

 © 2023 The Authors

Streeter DD, Spotnitz HM, Patel DP, John R, Sonnenblick EH (1969) Fiber orientation in the canine left ventricle during diastole and systole. *Circ Res* 24: 339–347

Teh I, McClymont D, Zdora M-C, Whittington HJ, Davidoiu V, Lee J, Lygate CA, Rau C, Zanette I, Schneider JE (2017) Validation of diffusion tensor MRI measurements of cardiac microstructure with structure tensor synchrotron radiation imaging. *J Cardiovasc Magn Reson* 19: 31

Tomer R, Ye L, Hsueh B, Deisseroth K (2014) Advanced CLARITY for rapid and high-resolution imaging of intact tissues. *Nat Protoc* 9: 1682–1697

Vetter FJ, Simons SB, Mironov S, Hyatt CJ, Pertsov AM (2005) Epicardial fiber organization in swine right ventricle and its impact on propagation. *Circ Res* 96: 244–251

Vinegoni C, Fumene Feruglio P, Courties G, Schmidt S, Hulsmans M, Lee S, Wang R, Sosnovik D, Nahrendorf M, Weissleder R (2020) Fluorescence microscopy tensor imaging representations for large-scale dataset analysis. *Sci Rep* 10: 5632

Wilson AJ, Han QJ, Perotti LE, Ennis DB (2023) Ventricular helix angle trends and long-range connectivity. In *Functional imaging and modeling of the heart*, Bernard O, Clarysse P, Duchateau N, Ohayon J, Viallon M (eds), pp 64–73. Cham: Springer Nature Switzerland

Yan GX, Shimizu W, Antzelevitch C (1998) Characteristics and distribution of M cells in arterially perfused canine left ventricular wedge preparations. *Circulation* 98: 1921–1927

Young RJ, Panfilov AV (2010) Anisotropy of wave propagation in the heart can be modeled by a Riemannian electrophysiological metric. *Proc Natl Acad Sci USA* 107: 15063–15068

Young AA, Legrice IJ, Young MA, Smaill BH (1998) Extended confocal microscopy of myocardial laminae and collagen network. *J Microsc* 192: 139–150

Zukić D, Jackson M, Dimiduk D, Donegan S, Groeber M, McCormick M (2021) ITKMontage: a software module for image stitching. *Integr Mater Manuf Innov* 10: 115–124

License: This is an open access article under the terms of the Creative Commons Attribution License, which permits use, distribution and reproduction in any medium, provided the original work is properly cited.

Expanded View Figures

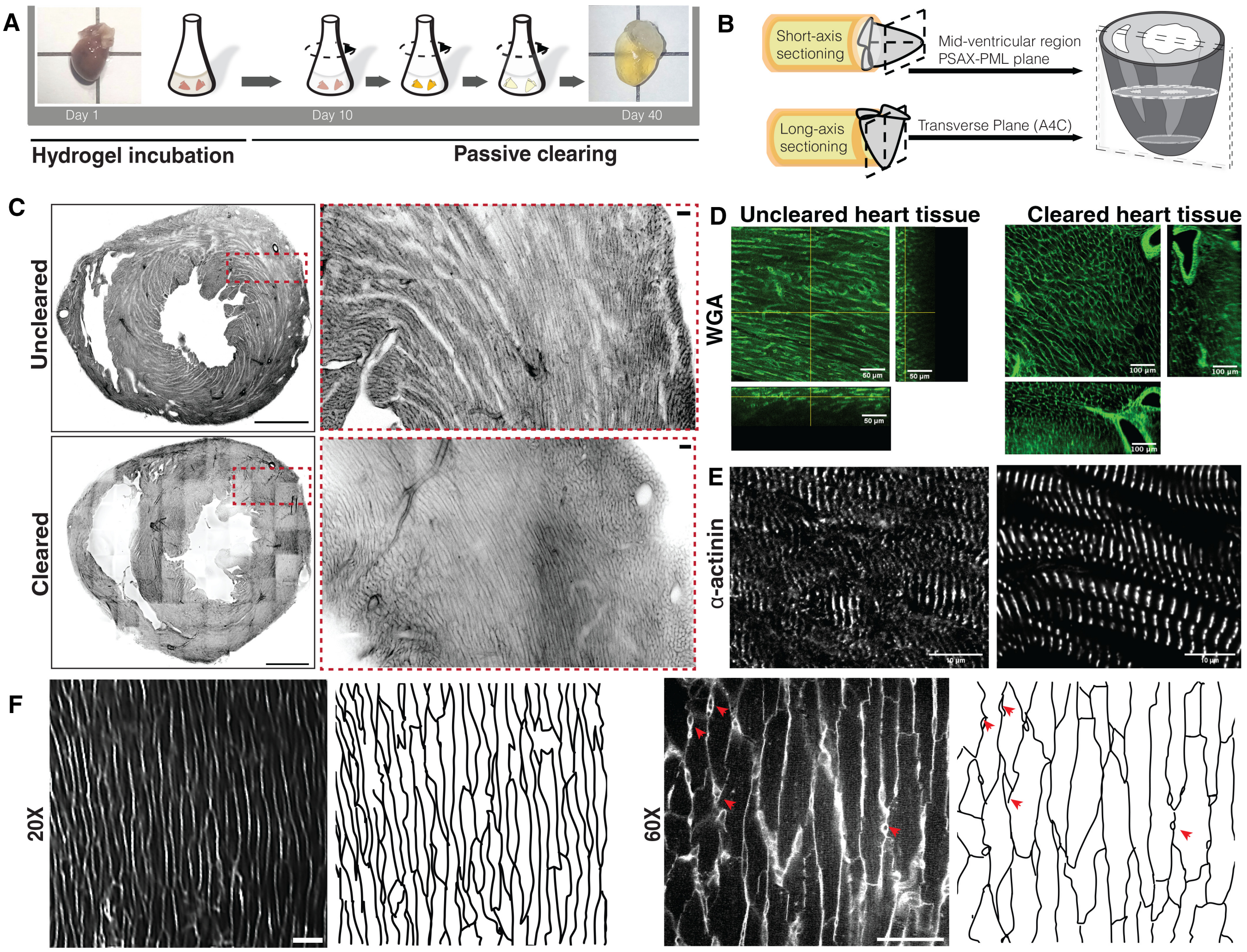

Figure EV1. A comparison of cleared and uncleared mouse heart tissue by imaging.

A A schematic illustration of the CLARITY method. The harvested heart tissues are incubated in a hydrogel/PFA mixture at 4°C for approximately 9 days. Following hydrogel incubation, the solution mixture with the heart tissues is polymerized at 37°C. The heart tissues are then excised from the polymerized hydrogel and shaken at 37°C with a clearing solution until they attain a desirable level of transparency (see Materials and Methods for details).

B The clarified heart is sectioned along its short- or long-axis. For the short-axis, a midventricular region that approximates the PSAX-PML (parasternal short-axis—papillary muscle level) plane was chosen. For the long-axis, a transversal plane that represents the A4C (apical four chambers) plane was used.

C–E A comparison of uncleared and cleared heart tissue sections stained with WGA and the alpha-actinin antibody.

F A comparison of 20× (2-μm isotropic resolution) and 60× (0.663 * 0.663 * 0.79 μm^3 x, y, and z resolution, respectively) cleared heart tissue sections stained with WGA. The raw and skeletonized images are shown for each magnification. The visible cell boundaries that are not of cardiomyocytes are marked with arrow heads (red) in the 60× images. The scale bar is 1,000 and 50 μm for the full view and zoomed in regions, respectively, in the WGA uncleared and cleared tissue images. The scale bar for the alpha-actinin images is 10 μm.

 © 2023 The Authors

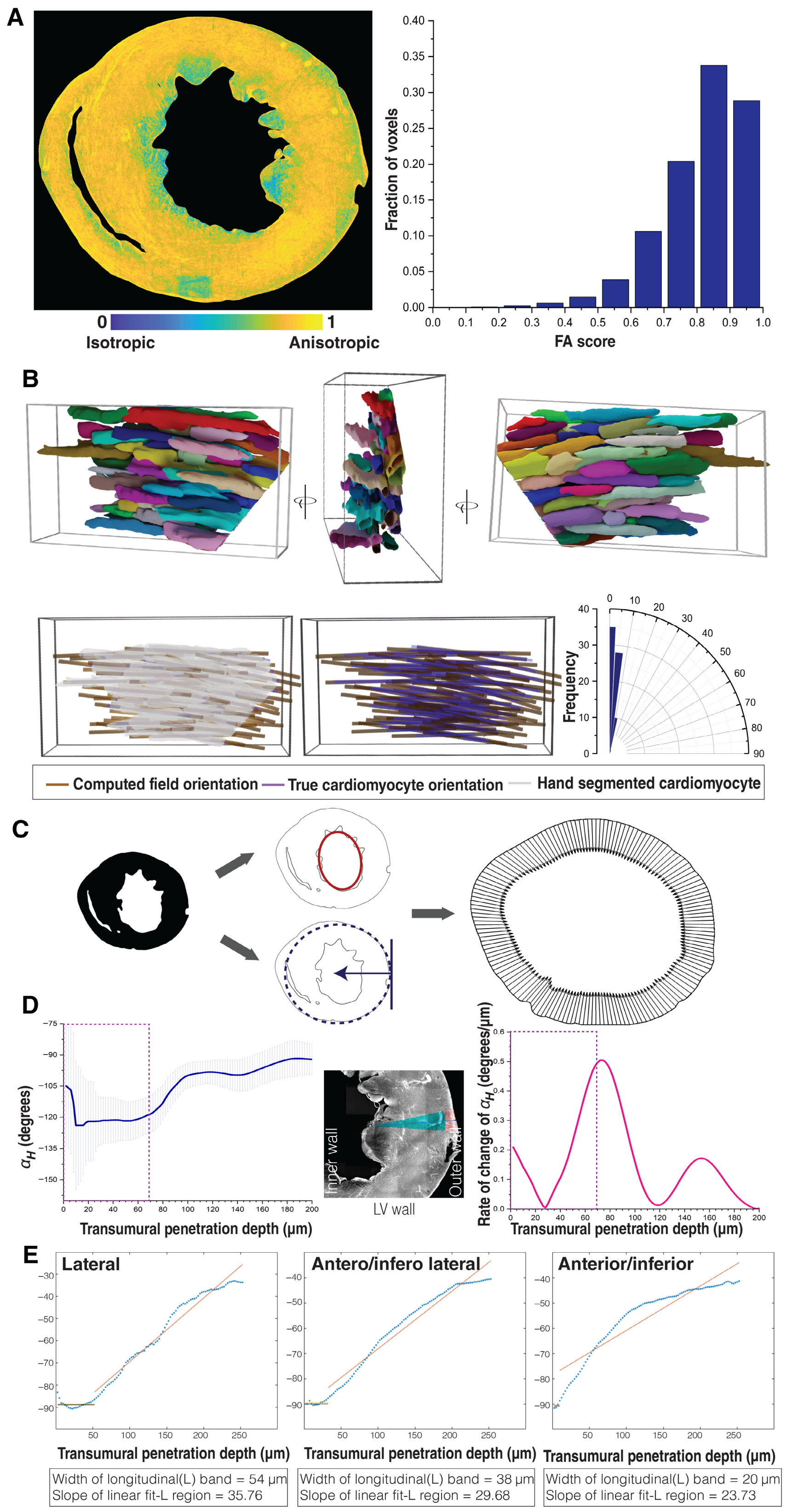

Figure EV2. The structure tensor method for orientation estimation.

A Fractional anisotropy scores are shown using a parula colormap (left) with a value in the range 0–1. The histogram (right) shows the fraction of pixels in each respective FA bin. The majority of the pixels have a high FA score, indicating the presence of dominant local orientations in the tissue stack. The color bar for the FA score is as indicated.

B Representative 3D views of hand-segmented cardiomyocytes with randomly assigned colors to each cell (top panel). A representative 3D view of ground truth orientation (purple) based on the second moment matrix for hand-segmented myocytes (gray) on the left, with the estimated field orientation from the WGA image (golden yellow) using a structure tensor approach (Materials and Methods) in the middle (bottom panel). The magnitude of the difference between the ground truth and the estimated orientation in degrees is illustrated with a graph on the right. The mean difference between two ground truth and estimated orientations is 5.98° ± 2.3°.

C Helix angle calculation: Masking (left) followed by centroid estimation (middle, top); Masking the short-axis section and estimating the tangent plane and normal for the penetration axis (middle, bottom). The set of penetration directions is shown on the right, from outer to inner wall.

D αH plot (left), region of LV wall analyzed (middle) and rate of change of αH calculated over a neighborhood of 15 voxels. The region marked by a dashed box represents the outer wall longitudinal cells, where the rate of change of αH is initially small and then increases sharply as one approaches the middle wall region, after which it plateaus and then increases again.

E Line fits for αH plots were calculated for the lateral, antero/infero lateral, and anterior/inferior regions, with the extent of the outer wall longitudinal cells shown by length of the first line in each plot.

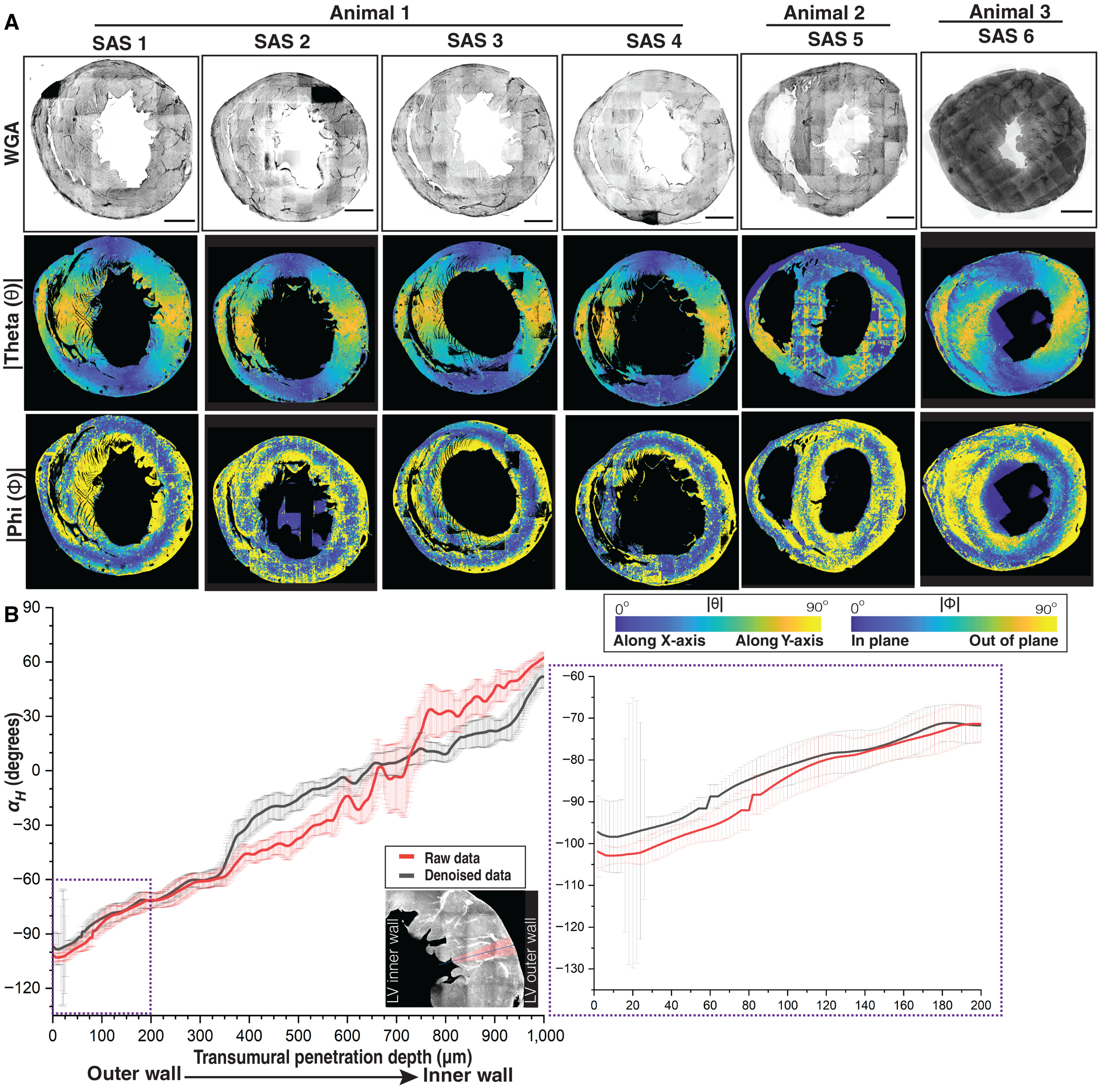

Figure EV3. WGA staining and angular colormaps of different short-axis sections.

A A maximum intensity Z-projection of the WGA-stained short-axis section from different mouse hearts as indicated, shown in grayscale, with the |Φ| and |θ| angles for cell orientations shown using parula colormaps, with the color bars as indicated. The scale bar is 1,000 μm.

B A comparison of αH estimates from raw (red line) and denoised short-axis section image stacks (black line) are shown for comparison. The region of the LV wall analyzed is shown in the bottom left panel, with a zoom in on the first 200 μm on the bottom right. The transmural penetration direction is from the outer to the inner LV wall. The raw data agree with denoised αH estimates in most of the places; however, we observed that estimation was much more consistent and robust to nonuniform illumination changes with denoised data. The intensity between two fields of view (~ 350 μm in transmural penetration depth) and the αH estimates begin to deviate marking the edges.

© 2023 The Authors

Figure EV4. Analysis of a short-axis section of a rat heart.

A A short-axis view of the ventricular chambers of a rat heart, sectioned at PSAX-PML (parasternal short-axis—papillary muscle level). A maximum intensity projection of the WGA stain is shown in grayscale, with the |Φ| angle shown using a parula colormap. The scale bar is 1,000 μm.

B–D Zoomed in regions from the left ventricle, septum and right ventricle, with the WGA stain shown in grayscale and the |Φ| angle shown using a parula colormap. The scale bars are 1,000 μm 100 μm for (A) and (B–D), respectively. The color bar is as indicated.

© 2023 The Authors

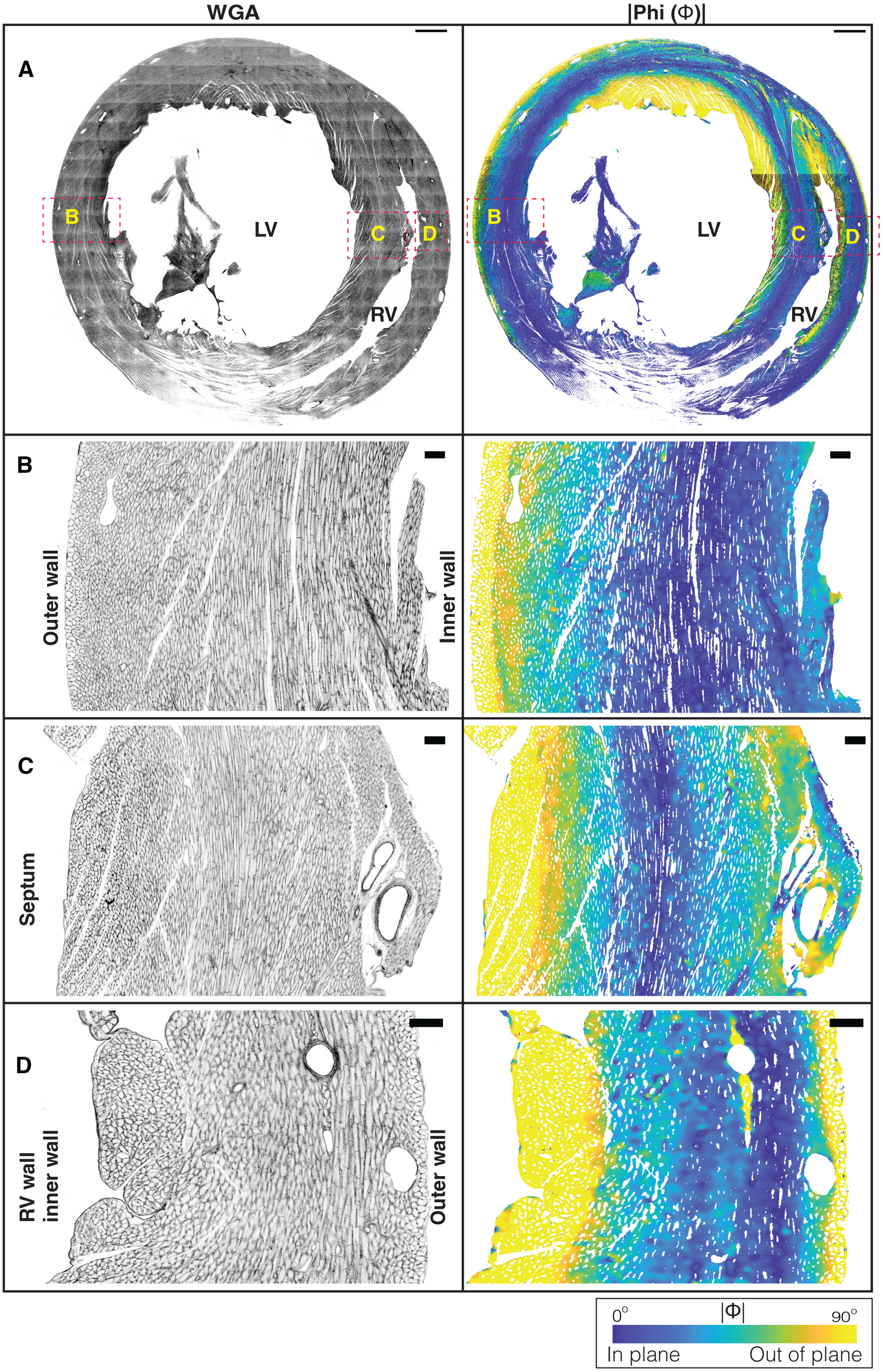

Figure EV4.

© 2023 The Authors

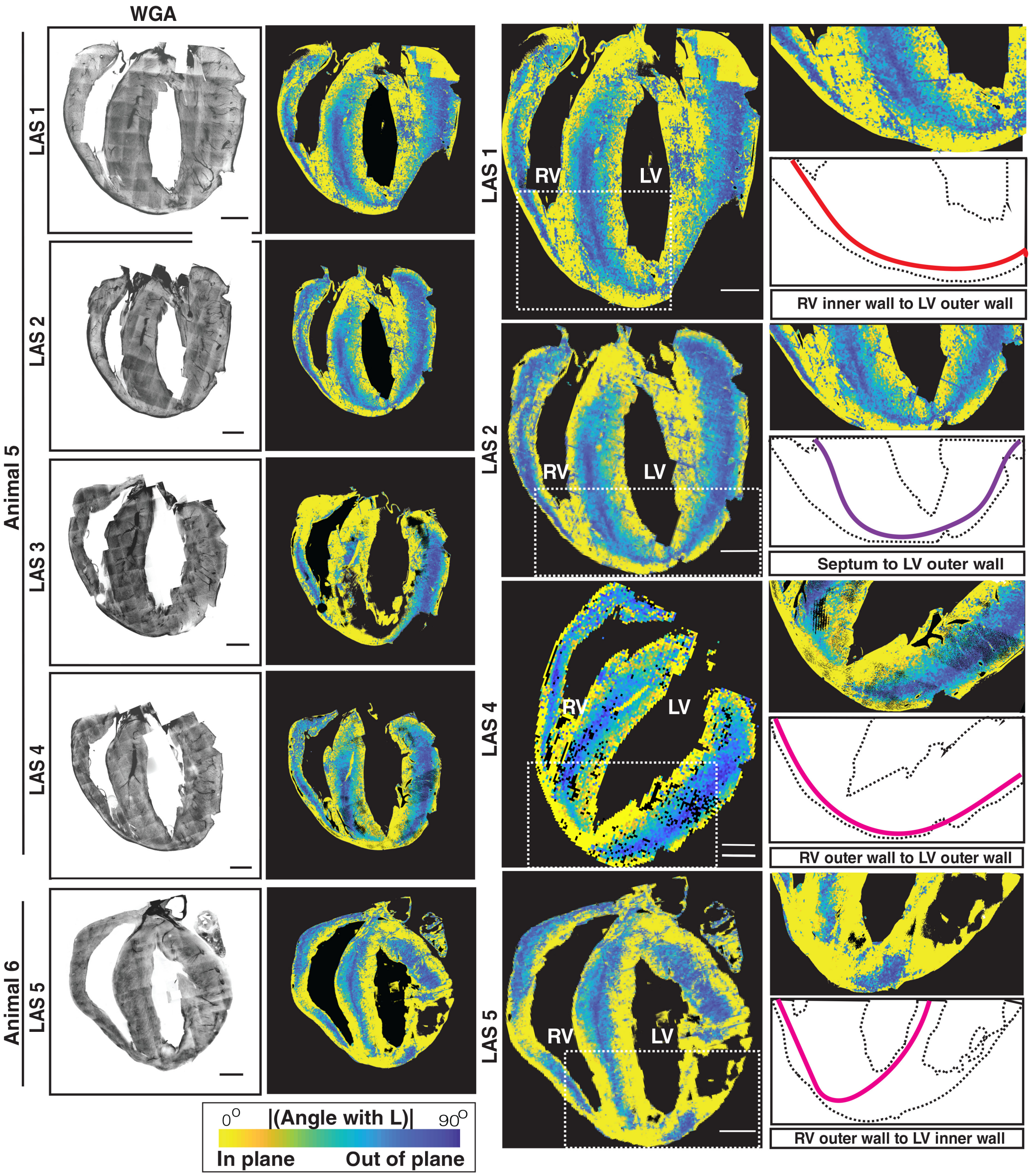

Figure EV5. WGA staining and colormaps for the magnitude of the angle with the longitudinal axis, for different long-axis sections and their connections at the apex.

Five mouse heart ventricle long-axis sections are shown as maximum intensity Z-projections of the WGA stain, with the magnitude of the angle between the aggregate cell orientation and longitudinal axis shown with a parula colormap marked, with a white dotted box highlighting the apex region under consideration (left column). A zoomed-in view of the highlighted apex region from the respective long-axis sections and illustration of long-axis connections between different ventricular walls is also shown (right column). The continuity of the long-axis fibers from the septum to the left ventricle outer wall, from the right ventricle inner wall to the left ventricle outer wall, from the right ventricle outer wall to the left ventricle outer wall and from the right ventricle inner wall to the left ventricle inner wall, is highlighted in the first, second, third, and fifth rows, respectively. The colorbar is as indicated, and the scale bar is 1,000 μm.

Cardiomyocyte orientation recovery at micron scale reveals long-axis fiber continuum in heart walls

Drisya Dileep*† Tabish A. Syed*‡ Tyler F. W. Sloan§
Perundurai S. Dhandapany† Kaleem Siddiqi‡ Minhajuddin Sirajuddin†

Appendix Table of Contents

Appendix Figure S1 2

Appendix Figure S2 3

1 Introduction to Methods 4

2 Biological Methods 5

2.1 Experimental procedures 5
2.2 Tissue preparation 6
2.3 Alignment of different short-axis sections 8
2.4 Analysis of short-axis sections from uncleared mouse and rat hearts 8

3 Computer Vision Methods 9

3.1 Deconvolution of acquired data 9
3.2 Denoising 9
3.3 Stitching 3D blocks 10
3.4 Orientation field estimation 11
3.5 Computation of the Helix Angle 11
3.6 Smoothing the Estimated Orientation Field 11
3.7 Validating Orientation Estimates 12
3.8 Φ, θ and α_H calculation and colormap generation 13
3.9 Computing the transmural rate of change of α_H 13
3.10 3D Rendered Visualizations and Animations 13

Appendix Table S1 15

*Equal Contribution
†Institute for Stem Cell Science and Regenerative Medicine, Bangalore, India.
‡School of Computer Science and Centre for Intelligent Machines, McGill University, and MILA - Québec AI Institute, Montréal, Canada.
§Quorumetrix Studio, Montréal

Appendix Figure S1

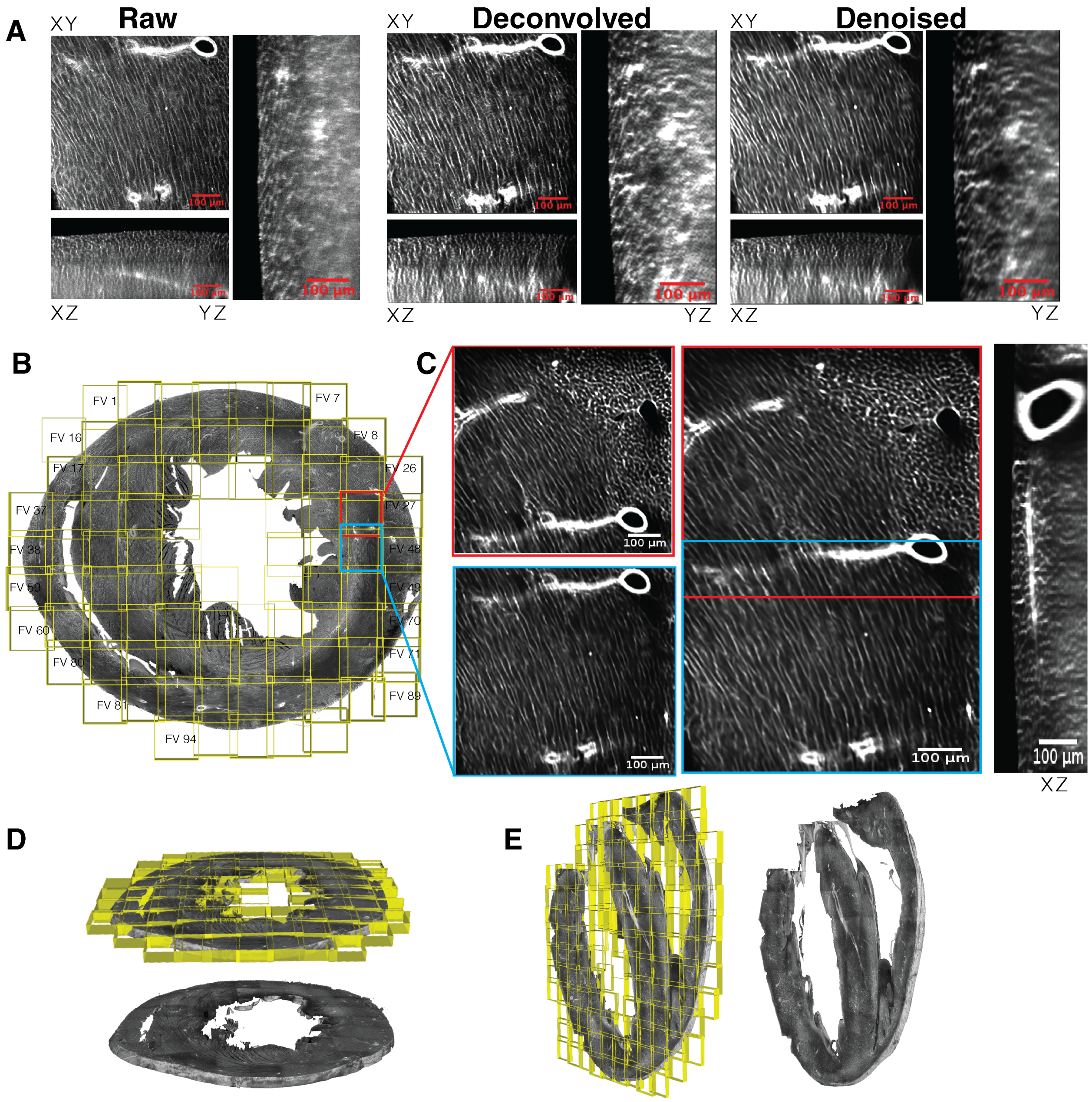

Appendix Figure S1: Preprocessing and stitching individual fields of view.

A. A representative field of view (left), followed by deconvolution (middle) and denoising (right). The orthogonal views (XZ and YZ) show an improvement in the signal to noise ratio towards the deeper Z-sections. The scale bar is 100 microns.

B. Individual fields of view (FVs) overlayed as a grid on the reconstructed full short-axis section. The individual FVs were imaged using a snake pattern, row by row. Each FV is 320 microns2 in dimension and has a 25% overlap with its neighboring FVs.

C. Left panel; An example showing the stitching of two neighboring FVs (FV28 and FV47, shown in red and blue boxes, respectively). Middle panel; A zoomed in view of the stitched result, showing the alignment of features in the common region. Right panel; An XZ/YZ view of the common region. The scale bar is as indicated.

D-E. 3D-views of the stitched short-axis and long-axis sections, with the individual FVs shown in yellow in an overlayed grid pattern.

Appendix Figure S2

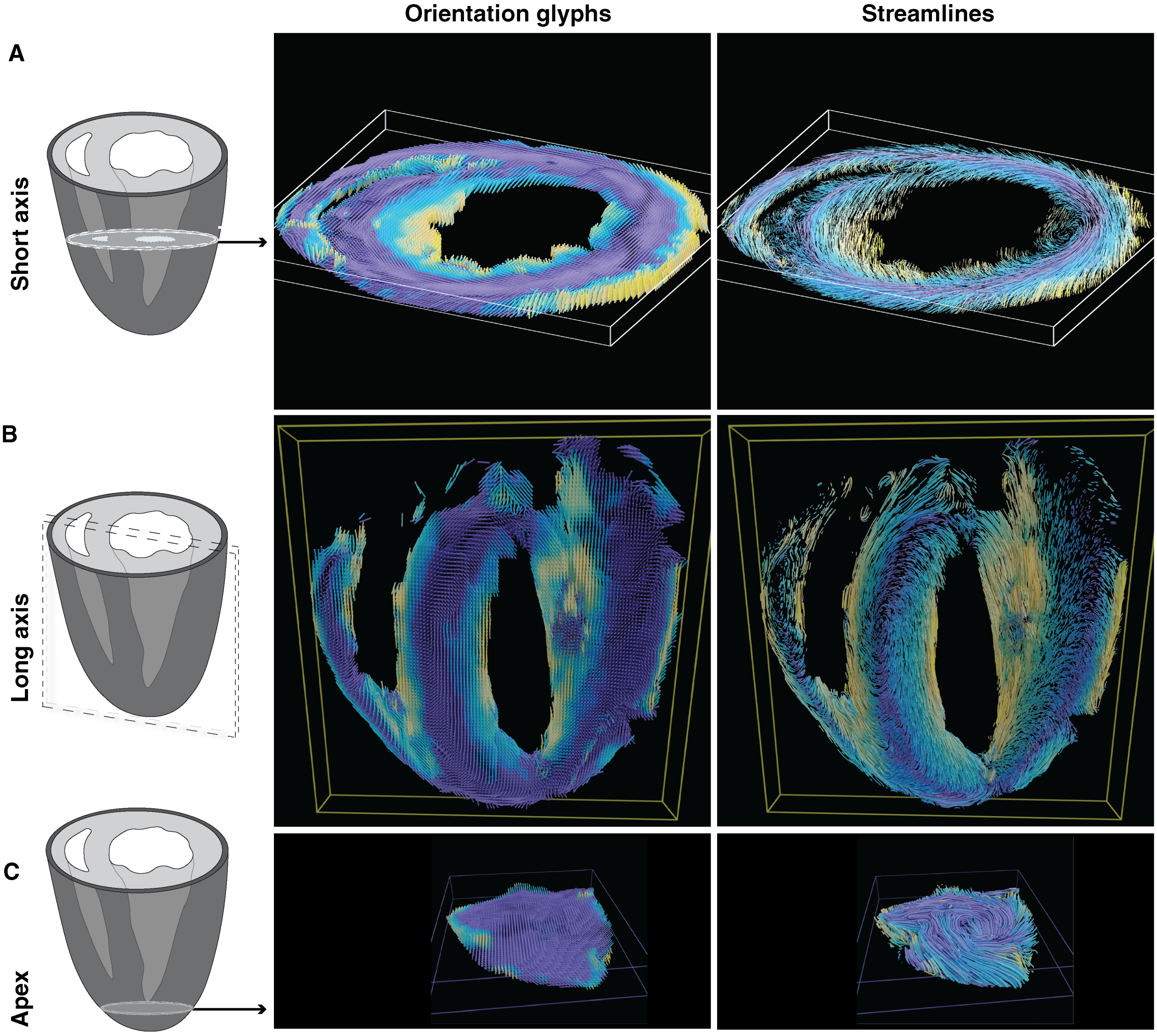

Appendix Figure S2: Long-axis and circumferential myofibers in the heart ventricular walls.

A. A short-axis (mid-ventricular region) section.

B. A long-axis (transverse section).

C. An apical section.

Data information: Schematics of the heart ventricle walls and the heart sections analyzed (left). Structure tensor based orientations are visualized as glyphs (middle) and streamlines (right) (Methods). The colors follow a parula colormap, where the blue and yellow tones indicate orientations that are in or out of the short-axis plane, respectively.

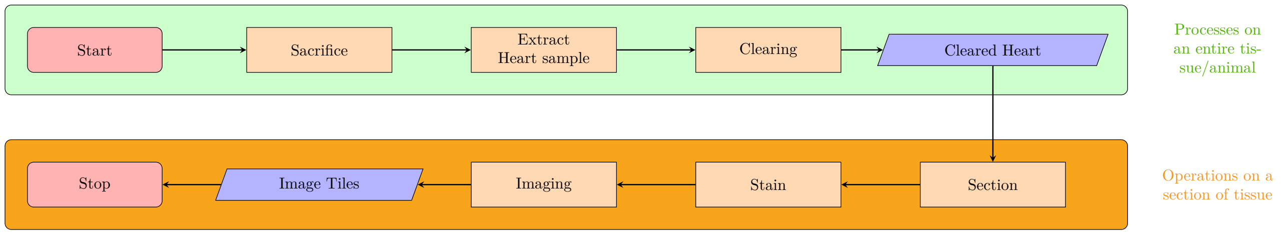

Appendix Figure S3: A flowchart of the steps in biological tissue preparation. The top row depicts operations performed on the entire tissue (or animal) while the bottom row depicts operations performed on sections of tissue.

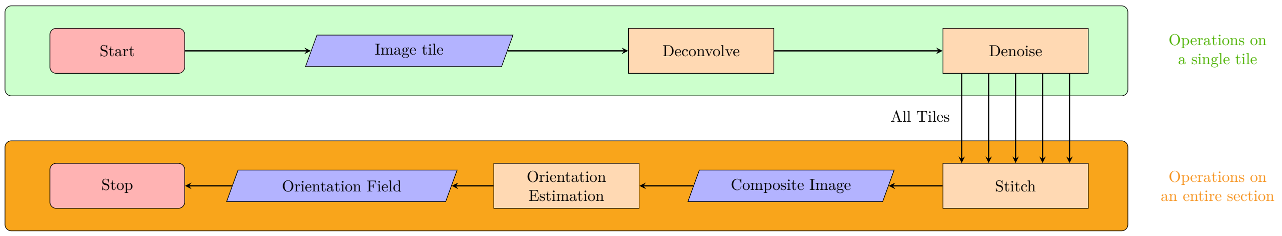

Appendix Figure S4: A flowchart illustrating the computational pipeline, starting from input image tiles, to generate a composite tiled image and a dense myocyte orientation field. The top row shows operations performed on individual image tiles while the bottom row shows operations performed on the entire stitched composite image.

1 Introduction to Methods

Here we present a detailed account of the steps used to recover myofiber organization in the mouse heart. All experiments were performed on wild type female mice of the C57BL/6 strain. All the experimental animals used in this study were maintained in the NCBS/inStem Animal Care and Resource facility in well ventilated cages with 12-hour light/12-hour dark cycles. The heart samples were collected from mice aged between 6 and 8 weeks. In Section 2 we describe the biological tissue preparation methods up to the imaging of the tissue samples using a confocal microscope. The flowchart in Fig. **S3** outlines the steps used for preparing heart samples and then tissue sections for imaging. In Section 3 we describe the computational methods used to estimate the cellular orientation of myocytes and to then reconstruct streamlines to represent the orientations of myofibers in entire heart sections. The flowchart in Fig. **S4** depicts the computational steps for generating a field representing myocyte orientations, starting from 3D images of small tiles of heart tissue sections.

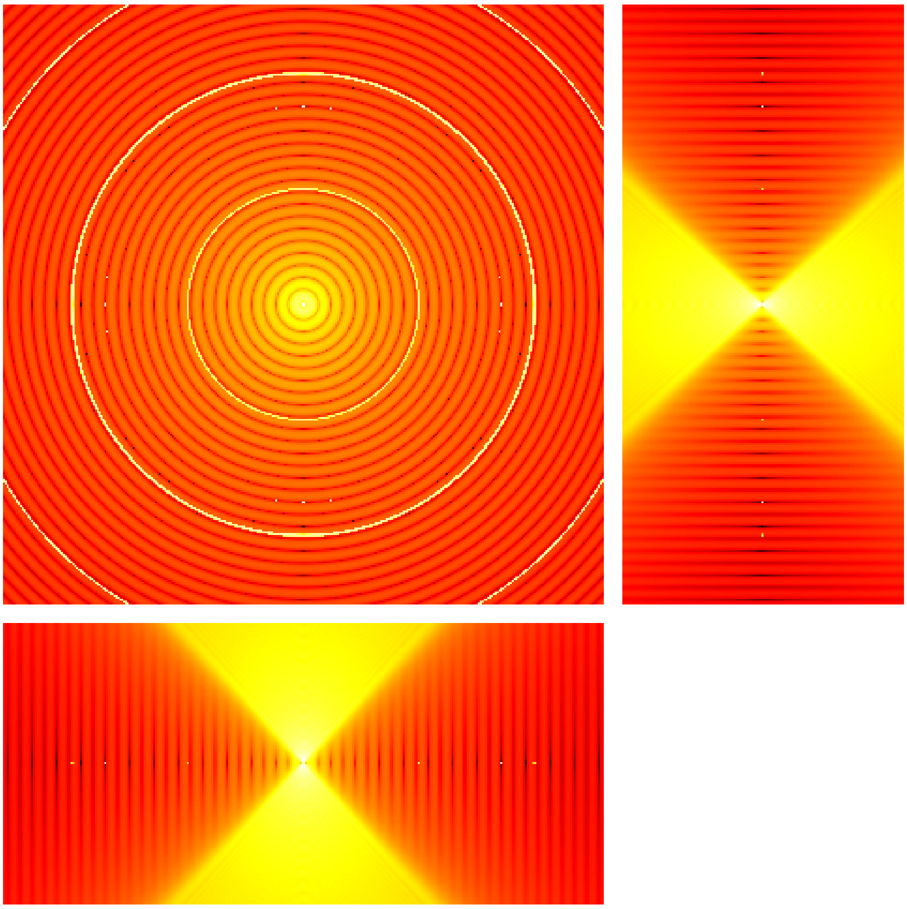

Appendix Figure S5: A visualization of the middle planes of a 3D point spread function (PSF) along the XY (top left), XZ (bottom left) and YZ (top right) directions, shown in log intensity scale (increasing from black through yellow and red to white).

2 Biological Methods

2.1 Experimental procedures

CLARITY based clearing protocol applied to the Mouse Heart: Each animal was sacrificed and its heart was gently accessed by cutting the abdominal cavity. The heart was then immediately perfused to remove blood and clots from the tissue. A small incision was made in the right atrium to facilitate fast perfusion of the heart chambers. The perfusion was carried out manually using a 26 Gauge syringe needle, inserted at an inclined angle, in the apex region of the right ventricle. We injected 10X phosphate-buffered saline solution (PBS) with a stock solution containing 1.37 M NaCl, 27 mM KCl, 100 mM Na_2HPO_4, and 18 mM KH_2PO_4, with the pH adjusted to 7.4. Initially, ice cold heparinized 1X PBS was passed through the syringe, followed by ice cold 4% paraformaldehyde (PFA). Subsequently, a hydrogel monomer solution consisting of 4% acrylamide, 4% PFA, 0.5% Bisacrylamide and 0.25% photo-initiator 2, 20-Azobis[2-(2-imidazolin-2-yl)propane] dihydrochloride (VA-044, Wako Chemicals USA) in PBS was perfused through the heart, as described previously for CLARITY based clearing of brain tissue [1]. The fixed mouse heart sample was transferred into a 50ml tube and incubated at 4 °C for seven days in the hydrogel monomer solution. The fixed heart

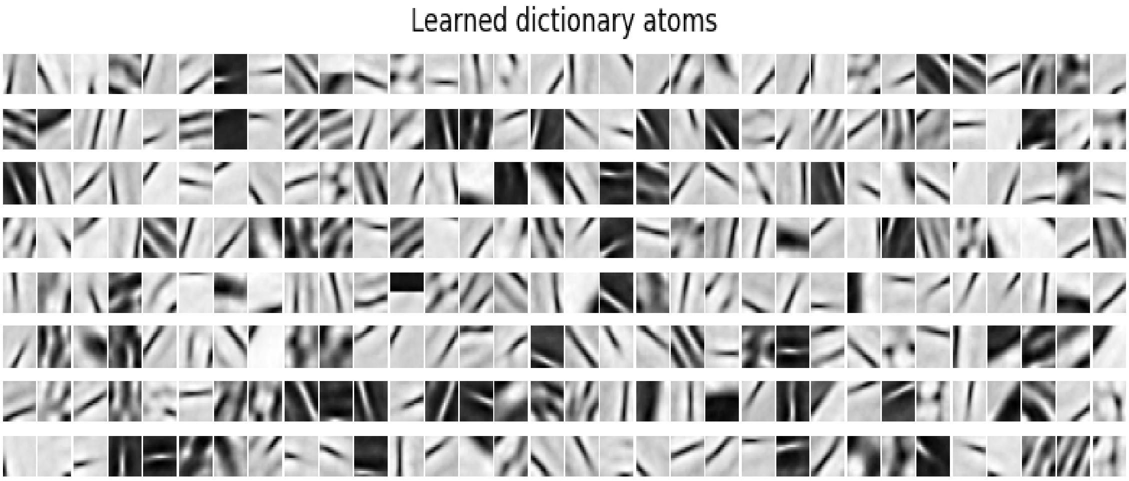

Appendix Figure S6: A visualization of the dictionary atoms learned for the mouse cardiac tissue microscopy image dataset SAS1.

tissues were then degassed for 10 minutes using a vacuum chamber at room temperature. To initiate tissue-hydrogel hybridization and polymerization, the processed heart tissues were then incubated for 3 hours at 37 °C. After polymerization, excess gel material was carefully removed by gently rubbing the tissue with soft tissue wipes. The tissue was transferred to 50 ml tubes for 1X PBS washes, which were carried out 3 times for a duration of 10 minutes each time. The tissue was further incubated with a clearing buffer (8% SDS and 4% boric acid in 1X PBS (pH 8.5)) for 20-30 days at 37 °C, in a shaking incubator (180 rpm) with a buffer exchange occurring every week. This CLARITY based approach applied to the heart tissue samples resulted in transparent tissue (Figure 1 and Supplementary Figure 1a) which could be imaged using a confocal microscope.

2.2 Tissue preparation

Sectioning: The cleared heart tissue was affixed with superglue along either its short axis or long axis orientation in a specimen tube (Compresstome® VF-300 OZ, Precisionary instruments). The specimen tube was a cylindrical holder with its outer rim fixed with a movable inside platform (or stage). The glued heart tissue was embedded in 2.5% low-melting agarose. For section along the short axis the tissue was placed so that the base of the heart was touching the stage of the specimen tube. For long axis sections, the tissue was kept in a plane on the stage, allowing for its four chambers to be seen. We used the compresstome to obtain 500μm thick tissue sections with an oscillation frequency of 7 units and a speed of 1.5mm/sec. The compresstome blade was kept close to the specimen tube, enabling the compression effect of sectioning to be distributed perpendicular to the sectioning axis. The tissue sections were collected in 1X PBS solution in the buffer tray of the compresstome. Each section was carefully transferred to one of 24 well plates, filled with 1X PBS, while maintaining the sectioning order. For this study, the short axis sections were approximately 3mm away from the apex and the long axis sections were approximately 3mm from the opposing outer walls of the heart. The

apex sections were cut in the short axis plane from the apical tip of the heart.

Staining: Each 500 µm processed section was washed in 1X PBS thrice over a day and permeabilized using a buffer containing 1% Triton X-100 (H5141, Promega) in PBS (PBST) for one day in a 37 °C incubator shaker. Subsequently, the tissue sections were incubated in 150 µg/mL of Alexa Fluor^{TM} 633 conjugated wheat germ agglutinin (WGA, W21404, ThermoFisher) for one day, to stain the cell membranes. The samples were washed with 1X PBS (3 times for 10 minutes each) before incubating them in imaging media (RIMS). For the preparation of RIMS, 40g of Histodenz (Sigma, D2158) was dissolved in 30 ml of 0.02 M phosphate buffer with 0.01% sodium azide, pH 7.5, resulting in a final concentration of 88% w/v Histodenz. The labelled tissue samples were incubated in RIMS until the tissue became more transparent [9]. All the staining and washing steps were carried out at room temperature, with gentle shaking. The cleared tissue samples were mounted with fresh RIMS solution using spacers (IS002, SUNjin Lab, Taiwan) of 500 µm such that the tissue was sandwiched between coverslips of size $60mm \times 20mm$.

For image acquisition, we used an Olympus FV3000 microscope. Images were first obtained using a lower magnification objective (Olympus PlanApo 1.25X/ air objective) to image a complete area of the heart tissue section. This low-resolution image was used to map high-resolution imaging areas of interest, using the Olympus fluoView^{TM} software. Then, micron scale imaging was carried out with the Olympus UCPLFN 20X Corr M32 85 mm scale air objective (NA=0.73). We used a 640 nm laser line for excitation and FV3000 high sensitivity spectral detectors (gallium arsenide phosphide (GaAsP) photomultiplier tube (PMT)) for detection of emission over a range of $650-670nm$. Each field of view covered approximately 320×320 pixels, with a voxel size of $1.98 \times 1.98 \times 1.98$ µm^3 and a depth of $\sim 300\mu m$. Here we under-sampled in the X and Y directions to obtain an isotropic voxel resolution, equivalent to sampling interval in the Z direction. Using the fluoview-map function, we ensured that the acquired 3D images were continuous, and had at least 25% overlap with their respective neighbouring fields of view. To minimize the laser attenuation at deeper regions of the tissue sample, the laser power was corrected (i.e., increased) with the help of the Bright Z function, with a manual judgement based on the quality of the intensity obtained at deeper layers. Each field of view was manually corrected for laser power intensity, increasing this by up to 10% with increased depth. The images were acquired following a snake pattern from row to row. Image reconstructions were performed using computer vision algorithms (as described in Section 3) using custom-written MATLAB and C++ scripts. After imaging, the heart tissue samples were stored in RIMS at room temperature and protected from exposure to light. In order to observe other cell types or Z-disc arrangement (α-actinin) as shown in Extended Data Fig. 1, a 60X magnified imaging of cleared tissue was carried out using PlanApo N 60X oil objective (NA=1.42) with a scan size of 320×320 pixels, with a voxel size of $0.66 \times 0.66 \times 0.79$ µm^3.

2.3 Alignment of different short-axis sections

We aligned the short axis datasets to ensure uniformity across different short axis sections, using the AHA classification and the capillary vessel description for the PSAX-PML level, used in echocardiography studies. We used the SAS3 dataset (Table1) as a reference. First, we aligned the posterior and anterior papillary muscles to the positions of the anterior and inferior regions globally. All other datasets were aligned using the outer wall and papillary muscle morphology to the SAS3 dataset, with the help of a MATLAB script and the ImageJ package. To obtain consistency between section alignments at a coarser scale some datasets were reflected (horizontally or vertically) when needed, using ImageJ. For example, the SAS2 and SAS4 samples both required a vertical reflection. The MATLAB script we wrote takes the SAS3 dataset as a reference and shows it as a transparent layer. This transparent layer can be rotated by an angular value to allow for fine alignment changes. Once the angular value for in-plane rotation had been determined, the short axis datasets were rotated in-plane and saved using ImageJ. The SAS3 and SAS4 had similar morphology. The SAS1 dataset required a $-10\,°C$ in-plane rotation and the SAS2 dataset required a $-7\,°C$ in-plane rotation. The SAS5 dataset was manually rotated by $180\,°C$ in-plane. At the end of this process, all the short axis datasets had a consistent alignment according to the AHA classification, including the positioning of major blood vessels.

2.4 Analysis of short-axis sections from uncleared mouse and rat hearts

We analyzed uncleared hearts from mouse (SAS7) of 1.5 months old and also from a different species, a wild-type Wistar strain of a male rat (RSAS1), approximately 8 weeks in age, which had been scheduled for culling in the NCBS/inStem animal facility. Once the mouse/rat had been sacrificed we performed a similar procedure as described in Section 2 . Once the perfusion was completed, the heart was excised and stored in 4% PFA at $4\,°C$. To enable the tissue to withstand freezing temperatures, the fixed heart was incubated in 30% sucrose for 5hrs before sectioning. The mouse/rat heart was cut into two thick blocks perpendicular to the long axis of the heart (i.e., short-axis-views). The resulting mid-ventricular region was suitable for cryo-sectioning. On the sectioning day, the mouse/rat heart was inserted in a mould containing tissue freezing medium and allowed to solidify at $-20\,°C$. The frozen sample was attached to a holder for cryostat (slee mev+), where the heart specimen was placed perpendicular to the long axis of the heart and sectioned into $100\,\mu m$ slices from the midventricular region. The sections were carefully transferred to 24 well plates containing 1X PBS, and were then washed (3 times for 10 minutes each) to remove freezing media. Afterwards, the tissue sections were incubated in $150\,\mu g/mL$ of Alexa Fluor^{TM}633 conjugated wheat germ agglutinin (WGA, W21404, ThermoFisher) for one day to stain the cell membrane. For α-actinin staining of uncleared or cleared mouse tissue, the tissue was blocked with 2.5% BSA + 2.5% neonatal goat serum in 1X PBST (1% Triton X) for 2hours. 1:200 dilution of primary antibody (Monoclonal anti-α-actinin rabbit antibody, Cell signalling, D6F6, 6487) and 1:400 dilution of secondary antibody (Anti Rabbit alexa-647 Goat antibody, Life technologies, A21245) were used sequentially with an incubation of a day at room

temperature. The samples were washed with 1X PBS (3 times for 10 minutes each) and incubated in RIMS subsequently for another day. We used a positively charged glass slide for mounting the uncleared mouse/rat heart tissue sections and custom made 100 µm spacers (100 µm plastic sheets). The stained heart sections were placed in this glass slide set up with RIMS and sealed with a cover glass in preparation for imaging. We used an Olympus FV3000 microscope and a lower magnification objective (Olympus PlanApo 1.25X/ air objective) to image the complete area of the tissue section to determine the best plane of view. This low-resolution image was used to map the high-resolution imaging area of interest using the Olympus fluoView software. The micron scale imaging was done with the Olympus UCPLFN 20X Corr M32 85 mm scale air objective (NA=0.73). Each field of view consisted of 320 × 320 voxels per slice, with a voxel size of 1.98 × 1.98 × 1.98 μm^3, over a depth of 10 µm and 50 µm for mouse and rat respectively. As with the cleared mouse hearts, we undersampled in the X, Y directions to obtain isotropic pixels at the resolution of the sampling in the Z direction. We used automatic tile acquisition via the fluoView software platform. Using the fluoview-map function, we ensured that the acquired 3D images were continuous with an overlap of 25 % with their neighbouring tiles, regulated by a motorized stage of the microscope. The fields of view were obtained row by row, following a snake pattern, and were then stitched using custom-built software.

3 Computer Vision Methods

3.1 Deconvolution of acquired data

Since the confocal images were acquired in three dimensions the resulting data was blurred in a manner that depended on the shape of the point spread function (PSF) of the microscope. A pseudo-color image of PSF generated based on our microscope settings is shown in Supplementary Information Figure (SI-Fig.) **S5**. To mitigate the effects of this blur we deconvolved each tile using an iterative Richardson-Lucy (RL) deconvolution method, with Total variation (TV) regularization (RL-TV), as described in [2]. The algorithm minimized the following objective function:

$$\min_I ||I \circledast PSF - I_o||_2 + \lambda ||I||_{TV}, \tag{1}$$

where $\circledast$ represents the convolution operation and TV is the total variation norm. In our implementation we set $\lambda = 0.01$ and processed each field of view for 20 iterations.

3.2 Denoising

As depth in the tissue increased, the signal to noise ratio decreased. As a result, the visual quality of the deeper layers was poorer than that of the shallow layers. To mitigate this effect we applied an unsupervised dictionary based method for denoising the images following the deconvolution stage. The method was based on the assumption that layers in the tissue are self similar so that the ultrastructure

of the tissue is similar in different depth layers of a single field of view. We trained a sparse ($m = 256$) element 2D dictionary of patches of size 16×16 using the sparse dictionary learning method of [6]. The shallow layers were relatively free from both depth and other optical degradation effects. The dictionary (D) was learned from data samples (x_i) from the shallow layers using the alternating minimization approach in [7]. The method involved alternating between between fixing D and solving the resulting basis pursuit denoising problem in Eq. (2) below

$$\min_{D \in C, \alpha \in \mathbb{R}^{256 \times n}} \frac{1}{n} \sum_{i=1}^{n} \left(\frac{1}{2} ||x_i - D\alpha_i||_2^2 + \lambda ||\alpha_i||_1 \right), \tag{2}$$

and fixing α and updating the dictionary D using coordinate descent. Here, $C = \{D \in \mathbb{R}^{256 \times 256}\ s.t.\ \forall j,\ ||d_j||_2^2 \leq 1\}$, and α_i are the sparse codes corresponding to data element x_i. We used a value of $\lambda = 0.15$ as the regularization parameter in our experiments and ran the optimization for 1000 iterations. The set of learned dictionary patches using tissue samples from the SAS1 dataset is shown in SI-Fig. **S6**.

The images acquired from deeper layers in the tissue sample can then be denoised using this learned sparse dictionary. To accomplish this, at each voxel in a degraded image we constructed a 16×16 patch centered at the voxel and estimated a denoised patch α by solving the sparse coding problem in Eq. (3) using a lars/homotopy method [3]:

$$\min_{\alpha \in \mathbb{R}^{256}} \frac{1}{2} ||x - D\alpha||_2^2 + \lambda ||\alpha||_1. \tag{3}$$

The final denoised image was reconstructed as an average of the denoised patches of the overlapping windows at each voxel. We learned a separate dictionary for each field of view so that the structure in one field of view did not affect the reconstruction in another.

3.3 Stitching 3D blocks

Each tissue section was too large to be imaged at once so we imaged multiple square shaped fields of view (tiles) of 320×320 isotropic voxels of length $1.98\mu m$ in each dimension, in the regions containing tissue samples. Adjacent tiles were set to have an overlap of 12.5% in every direction. We used the image registration method described in [8] for regions with valid data. The method involved a two stage registration process, with a local pairwise registration followed by a global registration. In the first local registration stage, we started with an initial guess for the location of each tile, derived from the microscope stage settings and assumed a 40 voxel (12.5%) overlap value. For every pair of adjacent tiles (a, b) we used the maximum phase correlation[5] based registration to estimate the relative shift, p_{ab} between the pair.

$$p_{ab} = \arg\max \mathscr{F}^{-}1 \left(\frac{A(\omega)B^*(\omega)}{|A(\omega)B^*(\omega)|} \right) \tag{4}$$

where, $\mathscr{F}^{-1}$ represents the inverse Fourier transform and $A(\omega)$ and $B^*(\omega)$ are the Fourier transform and the complex conjugate of the Fourier transform of tile a and tile b, respectively. For every imaged tile, a shift was computed with each of its 4-neighbours in the 2D imaging plane. This local pairwise registration process resulted in a refined list of (p_{ab}) pairwise relative shift values.

In the second global registration stage, a global optimal tile location of each tile $(p_a, p_b, ...)$ was computed with respect to the top left corner of the image. In all our datasets this corner tile was empty and was only used to define a common reference frame. The vector of optimal tile positions P for all tiles $T = \{a, b, ..\}$ was then given by

$$P = \arg\min \sum_{a \in T} \left(\sum_{b \in \mathcal{N}(a)} c_{ab} ||p_b - p_a - p_{ab}||_2^2 \right) \tag{5}$$

where c_{ab} was the correlation value between the pair a, b. This global registration was accomplished by solving an over-determined system of sparse linear equations for position [10]. This was done by iteratively eliminating all pairs of outlier pairwise distances. A local shift was labelled an outlier if it was over three standard deviations away from the mean shift.

Figures S2B and S2C illustrate the stitching process for a short axis section of a mouse heart. Two sample tiles are demarcated by red and blue bounding boxes.

3.4 Orientation field estimation

The orientation field was estimated at each voxel in the stitched and denoised image stack. We used the structure tensor [4] $s = G_\rho \circledast (\nabla_\sigma I)(\nabla_\sigma I)^T$, where G_ρ is a Gaussian with standard deviation ρ, $\circledast$ is the convolution operation and ∇_σ represents the intensity gradient at a Gaussian smoothing scale of σ. We used a noise scale $\sigma = 0.5$ and feature scale of $\rho = 3$ voxel units. The orientation was then set to align with the eigenvector of the structure tensor corresponding to the eigenvalue with smallest magnitude.

3.5 Computation of the Helix Angle

The helix angle α_H is typically defined in a manner that is relative to the local direction normal to the outer heart wall. To estimate the wall normal we computed a single pixel wide boundary of the heart in a short axis section and fit a circle tangential at each point along the outer boundary, using 80 sample points along the boundary in each direction. The direction of the heart wall normal was then associated with the inward radial vector of the circular fit (Figure S3B). The local helix angle α_H, as illustrated in Figure 2A, was calculated using the projection of the orientation onto the tangential plane defined by the heart wall normal. The angle varied from $-90°$ to $90°$, with $0°$ representing the in plane circumferential fibers and $\pm 90°$ representing fibers pointing out of the short axis plane, in the long axis direction of the heart.

3.6 Smoothing the Estimated Orientation Field

Given the thickness of tissue samples used in our study, imaging data in deeper layers, where light penetration was reduced, was noisy. In addition, optical factors including light scattering, photo bleaching and optical aberrations in the microscope lens, also diminished the image quality. To mitigate the affect of the reduced image quality on orientation estimation we averaged the orientation field over

small neighborhoods. Whereas orientations are directionless, their representation using the eigenvector with the smallest eigenvalue of the structure tensor is not. Two vectors whose components have the same magnitude but differ in signs represent the same orientation, so these direction vectors cannot be directly averaged component wise.

To smooth the orientations we first computed the rank-1 tensor constructed as $s = uu^T$, where s is a 3×3 matrix and u was the local unit direction vector. This rank-1 tensor was invariant to flips, since $s = uu^T = (-u)(-u)^T$. In fact, $s \in Gr(3,1)$, represents the Grassmann manifold of one dimensional subspaces (lines) in 3 dimensional Euclidean space. While it was possible to use the weighted Karcher mean to smooth the resulting tensors component-wise, an iterative approach to doing so was slow and did not scale well to handle large volumes of data. We therefore opted for an approximate strategy. We averaged the orientation tensors s component-wise using a local weighted average, and then projected back to the space of direction vectors. Specifically, the smoothed tensor $\hat{s}$ at any location $\mathbf{x}$ was given by

$$\hat{s}(\mathbf{x}) = \sum_{y \in Nbd(\mathbf{x})} w(\mathbf{y}-\mathbf{x})s(\mathbf{y}). \tag{6}$$

Here, $w(\cdot)$ is a scalar weight, which was empirically chosen to be a Gaussian with $\sigma = 4$, as defined below. The smoothed direction vector $\hat{u}$ at $\mathbf{x}$ was then given by the eigenvector of the $\hat{s}$ matrix corresponding to the eigenvalue with the largest magnitude. We only carried out smoothing in regions within the heart tissue by setting the weight $w(\mathbf{z})$ to zero in regions with missing data:

$$w(\mathbf{z}) = \begin{cases} \frac{1}{\sqrt{(2\pi\sigma^2)^3}} e^{-\frac{\mathbf{z}^2}{\sigma^2}} & \text{if } \mathbf{z} \in Supp(data) \\ 0 & \text{otherwise.} \end{cases} \tag{7}$$

3.7 Validating Orientation Estimates

To verify the accuracy of the orientations estimated using the structure tensor we hand segmented 70 myocytes in a field of view from the dataset SAS3, by manually tracing the boundaries of individual cells across multiple slices. The myocytes were marked by labeling the cytoplasm within each cell boundary, as signalled by WGA staining. The orientation of each hand segmented cell was then calculated using the second moment matrix of the interior voxels of each labeled myocyte. Each hand segmented cell was assigned a ground truth cell orientation derived from the eigenvector corresponding to the eigenvalue of the second moment matrix with the largest magnitude. We then associated a structure tensor based orientation estimate for the segmented cell using the average of the estimated field orientations over each of its interior voxels. A cell wise comparison revealed close agreement between the ground truth and the structure tensor based estimates of the orientations from the raw WGA images. For 70 hand segmented cells we observed an average difference of 6.13° with a standard deviation of 3.56°. Figure 1 and Supplementary Figure 3a provide a comparison of the ground truth myocyte orientations, represented by purple cylinders, and the structure tensor based estimated orientations, represented by golden yellow cylinders, with the manually segmented myocytes shown

in gray. We computed the Fractional Anisotropy (FA) score for each tensor, to confirm that the orientation estimates based on it were valid in that they reflected local elongation. The FA score as defined below in equation (8) measures the degree of anisotropy of the structure tensor, where the λ_i's are the eigenvalues of the structure tensor matrix.

$$FA = \sqrt{\frac{1}{2}} \frac{\sqrt{(\lambda_1 - \lambda_2)^2 + (\lambda_2 - \lambda_3)^2 + (\lambda_3 - \lambda_1)^2}}{\sqrt{\lambda_1^2 + \lambda_2^2 + \lambda_3^2}}. \tag{8}$$

An FA score close to one indicates strong directionality of the gradients at a location, while a value closer to zero reflects an isotropic region. The computed FA values were found to be closer to 1 for most of the tissue section, confirming that the WGA data reflected locally elongated cardiomyocytes.

3.8 Φ, θ and α_H calculation and colormap generation

The colormaps for Φ, θ and α_H were generated using the smoothed orientation field. We calculated the Φ and θ angles at each voxel with valid data and then mapped these angles to color values using a linear scale in the parula colormap.

To obtain the α_H colormaps we considered voxels with valid data and then computed the average value of the helix angle over all radial penetration lines overlapping at the voxel. A penetration line at a point (x,y) in a short axis plane was considered to overlap an integer valued voxel (i,j) when $i \leq x \leq i+1$ and $j \leq y \leq j+1$. Due to irregularities in boundary shape, which in turn affected the estimate of the heart wall normal, it was possible for a few isolated voxels, containing valid orientation estimates, to have no penetration line passing through them. The α_H value at each such location was set to the average α_H value over a 3×3 voxel neighbourhood.

3.9 Computing the transmural rate of change of α_H

To measure the rate of change of α_H in the transmural direction we compute the forward difference between its values along the outer to inner wall direction. This difference is computed by calculating the smaller of the angle between two successive α_H vectors. To ensure that these rate of change estimates are well defined, we smoothed α_H using a 1D Gaussian with $\sigma = 7$ voxels, along the transmural penetration direction, prior to computing the forward difference.

3.10 3D Rendered Visualizations and Animations

Orientation glyphs and streamlines were procedurally generated and rendered using a custom Python module within the open-source animation software Blender. For each sample volume, an orientation field and a tiff stack representing the WGA tissue staining were imported as N-dimensional NumPy arrays.

Glyphs The orientation field was represented by a 3-dimensional array of rotated cylinders, referred to as orientation glyphs. The input vector field was approximated by a 3-dimensional grid of equally spaced vertices, downsampled such that the number of vertices was not larger than 75,000. At each vertex of the downsampled grid, a cylinder primitive shape was created, and rotated proportionally to the components of the vector field at the vertex position. A parula colormap was applied to the cylinders proportional to the Φ angle, defined as the arc cosine of the absolute value of the z component of the vector field.

Streamlines Bidirectional streamlines were represented as curves extruded from polylines, whose points were computed as follows. A set of up to 25,000 points were selected from a random sample of voxels from the vector field, and each sample voxel location was the initial point for a streamline. Since the direction of the vector field represents the orientation of the tissue, and the sign of the orientation does not matter, each starting point initialized both a positive and negative streamline. Both positive and negative streamlines originated from a single starting point, and were grown by iteratively adding new points along the polyline. For each iteration, the location of the next point was calculated by adding a displacement proportional to the vector field and the current point, multiplied by the sign, such that the positive streamline is displaced by the positive value of the field, and the negative streamline by the negative value of the field. At each new position, the value of the field at that voxel acts to determine the position of the following point along the polyline. A parula colormap was applied to the streamlines in proportion to the Φ angle at the streamlines starting point within the vector field. To avoid artifacts arising where the streamlines extend beyond tissue, a binary mask of the tissue volume with the same dimensions as the vector field served as a boundary condition.

Appendix Table S1

<table>
<tr><th>Dataset</th><th>Orientation of dataset</th><th>Animal Sex</th><th>Derived from animal number</th><th>Cleared (by CLARITY) or Uncleared</th></tr>
<tr><td>SAS1*
SAS2*
SAS3*
SAS4*</td><td rowspan="4">Short Axis</td><td rowspan="7">Female</td><td>Mouse 1</td><td rowspan="3">Cleared</td></tr>
<tr><td>SAS5</td><td>Mouse 2</td></tr>
<tr><td>SAS6</td><td>Mouse 3</td></tr>
<tr><td>SAS7</td><td>Mouse 4</td><td>Uncleared</td></tr>
<tr><td>LAS1#
LAS2#
LAS3#
LAS4#</td><td rowspan="2">Long Axis</td><td>Mouse 5</td><td rowspan="3">Cleared</td></tr>
<tr><td>LAS5</td><td>Mouse 6</td></tr>
<tr><td>APEX1*
APEX2*
APEX3*
APEX4*</td><td>Short Axis</td><td>Mouse 1</td></tr>
<tr><td>RSAS1</td><td>Short Axis</td><td>Male</td><td>Rat 1</td><td>Uncleared</td></tr>
</table>

Appendix Table S1: A list of sections and animals used in this study (*/# indicate serial sections from same animal)

References

[1] Kwanghun Chung, Jenelle Wallace, Sung-Yon Kim, Sandhiya Kalyanasundaram, Aaron S Andalman, Thomas J Davidson, Julie J Mirzabekov, Kelly A Zalocusky, Joanna Mattis, Aleksandra K Denisin, et al. "Structural and molecular interrogation of intact biological systems". In: *Nature* 497.7449 (2013), pp. 332–337.

[2] Nicolas Dey, Laure Blanc-Feraud, Christophe Zimmer, Pascal Roux, Zvi Kam, Jean-Christophe Olivo-Marin, and Josiane Zerubia. "Richardson–Lucy algorithm with total variation regularization for 3D confocal microscope deconvolution". In: *Microscopy research and technique* 69.4 (2006), pp. 260–266.

[3] Iddo Drori and David L Donoho. "Solution of L1 minimization problems by LARS/homotopy methods". In: *2006 IEEE International Conference on Acoustics Speech and Signal Processing Proceedings*. Vol. 3. IEEE. 2006, pp. III–III.

[4] Hans Knutsson, Carl-Fredrik Westin, and Mats Andersson. "Representing local structure using tensors II". In: *Scandinavian conference on image analysis*. Springer. 2011, pp. 545–556.

[5] C.D. Kuglin and D.C. Hines. "The phase correlation image alignment method". In: *Proc. Int. Conf. on Cybernetics and Society*. IEEE, Sept. 1975, pp. 163–165.

[6] Julien Mairal, Francis Bach, Jean Ponce, et al. "Sparse modeling for image and vision processing". In: *Foundations and Trends® in Computer Graphics and Vision* 8.2-3 (2014), pp. 85–283.

[7] Julien Mairal, Francis Bach, Jean Ponce, and Guillermo Sapiro. "Online dictionary learning for sparse coding". In: *Proceedings of the 26th annual international conference on machine learning*. 2009, pp. 689–696.

[8] Stephan Preibisch, Stephan Saalfeld, and Pavel Tomancak. "Globally optimal stitching of tiled 3D microscopic image acquisitions". In: *Bioinformatics* 25.11 (2009), pp. 1463–1465.

[9] Bin Yang, Jennifer B Treweek, Rajan P Kulkarni, Benjamin E Deverman, Chun-Kan Chen, Eric Lubeck, Sheel Shah, Long Cai, and Viviana Gradinaru. "Single-cell phenotyping within transparent intact tissue through whole-body clearing". In: *Cell* 158.4 (2014), pp. 945–958.

[10] Dženan Zukić, Michael Jackson, Dennis Dimiduk, Sean Donegan, Michael Groeber, and Matthew McCormick. "ITKMontage: A Software Module for Image Stitching". In: *Integrating Materials and Manufacturing Innovation* 10.1 (2021), pp. 115–124.
