# Supplementary material for: Cardiomyocyte orientation recovery at micrometer scale reveals long‐axis fiber continuum in heart walls
Source: EMBO J. 2023 Sep 6;42(19):e113288. doi: 10.15252/embj.2022113288 (PMC10548172; doi:10.15252/embj.2022113288)
Supplement: Supplementary file 12 [file EMBJ-42-e113288-s009.pdf]

THE EMBO JOURNAL

Cardiomyocyte orientation recovery at micrometer scale reveals long-axis fiber continuum in heart walls

Drisya Dileep, Tabish Syed, Tyler Sloan, Perundurai Dhandapany, Kaleem Siddiqi, and Minhajuddin Sirajuddin
DOI: 10.15252/embj.2022113288

Corresponding author(s): Minhajuddin Sirajuddin (minhaj@instem.res.in) , Kaleem Siddiqi (siddiqi@cim.mcgill.ca)

Review Timeline:

Submission Date:	14th Dec 22
Editorial Decision:	24th Feb 23
Revision Received:	5th Jun 23
Editorial Decision:	7th Jul 23
Revision Received:	1st Aug 23
Accepted:	6th Aug 23

Editor: Kelly Anderson

Transaction Report:

(Note: With the exception of the correction of typographical or spelling errors that could be a source of ambiguity, letters and reports are not edited. Depending on transfer agreements, referee reports obtained elsewhere may or may not be included in this compilation. Referee reports are anonymous unless the Referee chooses to sign their reports.)

Dear Dr. Sirajuddin,

Thank you for submitting your manuscript for consideration by the EMBO Journal. It has now been seen by three referees whose comments are shown below. As you will see, the referees have raised concerns that the biological insight, specifically the newly identified longitudinal cardiomyocyte tracts, is not sufficiently supported by the data. That said, the referees are overall positive on the importance of the method provided. Therefore, I would like to invite you to submit a revised version of the manuscript, addressing the comments of all three reviewers. Unless you are prepared to provide more evidence to support your biological insights as suggested by the referees, we would ask that you readjust the language related to these findings and rather reframe this manuscript to be more protocol-focused. It is EMBO Journal policy to allow only a single round of revision, and acceptance of your manuscript will therefore depend on the completeness of your responses in this revised version. It would be good to discuss your plan to address the referee concerns and I am available to do so by email or zoom in the coming weeks. I have also attached a guide to revisions for your convenience.

When preparing your letter of response to the referees' comments, please bear in mind that this will form part of the Review Process File, and will therefore be available online to the community. For more details on our Transparent Editorial Process, please visit our website: https://www.embopress.org/page/journal/14602075/authorguide#transparentprocess

We generally allow three months as standard revision time. As a matter of policy, competing manuscripts published during this period will not negatively impact on our assessment of the conceptual advance presented by your study. However, we request that you contact the editor as soon as possible upon publication of any related work, to discuss how to proceed. Should you foresee a problem in meeting this three-month deadline, please let us know in advance and we may be able to grant an extension.

Thank you for the opportunity to consider your work for publication. I look forward to your revision.

Yours sincerely,
Kelly

Kelly M Anderson, PhD
Editor
The EMBO Journal
k.anderson@embojournal.org

Further information is available in our Guide For Authors: https://www.embopress.org/page/journal/14602075/authorguide

We realize that it is difficult to revise to a specific deadline. In the interest of protecting the conceptual advance provided by the work, we recommend a revision within 3 months (25th May 2023). Please discuss the revision progress ahead of this time with the editor if you require more time to complete the revisions. Use the link below to submit your revision:

https://emboj.msubmit.net/cgi-bin/main.plex

Referee #1:

In the manuscript of EMBOJ-2022-113288 entitled "Myofiber reconstruction at micron scale reveals long-axis fiber continuum in heart ventricular walls", Dr. Dileep and colleagues reported a prominent long-axis fiber system in the outer walls of the heart, distinct from the well-known helical myofibers and the longitudinal myofibers in the inner heart walls, by using microscopy and computer vision with 3D reconstruction of myofibers at the micron scale. This paper is well written and may address an important topic. Also, the quality of figure and video is good; however, it will be somewhat hard to understand, and there are some concern to be addressed.
1. The authors emphasized better understanding of myofiber geometry at the micron scale, compared with DT-MRI studies. Please discuss about it and clarify the clinical impact, rather than mentioning the association between pathological heart conditions and fiber organization.
2. This study is based on histological examination. In human study, only small samples from endomyocardial biopsy are obtained, while DT-MRI can provide noninvasively whole-heart assessment even as less spatial resolution.
Minor comments
1.Can they show the images without deconvolution and denosing? Is there any difference in results if not using deconvolution or

denosing?
2. Page 10, line 33 What do they mean as Figure 1a? Please modify the figure legend.
3. Page 3, line 26 Words such as PSAX-PML, HLA-4C need to be spelled out.

Referee #2:

Summary:
In this manuscript, Dileep et al apply the CLARITY tissue clearing process to mouse hearts (and one rat heart), apply a stain that allows visualization of cardiomyocyte boundaries (WGA). Sections of these hearts are imaged using confocal microscopy, and then processed using structure tensor analysis.
While this manuscript has some nice imaging data, in this reviewer's opinion importance of the novel "long-axis myofiber layer" is not supported by the data in the manuscript or the supplemental data.
Overall, I don't recommend this manuscript for publication in its current form. Larger sample sizes and additional imaging or other data - separate from the structure-tensor processed data - would be needed in order for it to be considered a genuine anatomical discovery. In this reviewer's opinion this manuscript would be better suited to focus on its excellent image processing and visualization.

Major concerns:
You refer to the "long-axis myofiber layer" "long-axis bands of fibers" "distinct from the known helical myofibers" "separate long-axis outer wall fiber system". Is there some sort of anatomic separation of this set of cardiomyocytes? Some sort of collagen structure or membrane? It seems that the distinctness of these cardiomyocytes from the rest of the myocardium is over-stated.
The authors refer to "myofibers" throughout. The heart doesn't contain myofibers, and in this reviewer's opinion the term should no longer be accepted for anatomic descriptions of the heart. Within a large voxel (say 1x1x1 mm) there can be considered to be a "net cardiomyocyte orientation" or "aggregate cardiomyocyte orientation". If you're referring to structure tensor data, refer to the eigenvector directions. If you're referring to tract/streamline, refer to tract/streamline orientation.
The text in the results section provides an insufficient summary of the experimental data. The majority of the text in the "results" section looks like it belongs in the methods or discussion section. Please refer to actual numerical results as much as possible, and avoid interpreting color maps. For me to consider a genuine "layer" of cells based on transmural helix angle I would want to see a clear discontinuity, which I don't see in your data.
The abstract should be much more specific. What breed of mouse? How many mice? What tissue clearing process? How were the images processed? What is the most important helix angle data?
pg 2 line 30-34 & pg 3 line 1-5 This paragraph is essentially wrong because of the Garcia-Canadilla et al 2019 paper (doi:10.1111/joa.13058), which you later misreport as using DT-MRI (pg10 line 12). Also Pope et al 2008 (doi:10.1152/ajpheart.00484.2008), Teh et al 2017 (doi:10.1186/s12968-017-0342-x).
pg 5 line 19-23 This would likely look different depending which colormap you used. You should stick to reporting angle numbers rather than colors from color maps.
pg 6 line 12-14 "entirely new discovery" To me the epicardial structures you're reporting look the same as those reported by Teh et al 2016 (doi:10.1016/j.pbiomolbio.2016.06.001) Figures 3 and 7, and probably others.
Why do you think the steep transition in epicardial helix angle is not observed in Pope et al 2008 (doi:10.1152/ajpheart.00484.2008) which also as micron scale data?
pg 6 18-21 You need to be much more descriptive in what Figure 3b is showing, and what resolutions the different colored lines are referring to, dotted versus solid etc.
pg 6 line 26-29 How confident are you that these are not just papillary muscles?
pg 8 line 9-11 "a resolution gain of about 3 orders of magnitude". I don't think this is correct. Please re-check Garcia-Canadilla et al 2019 (doi:10.1111/joa.13058), Pope et al 2008 (doi:10.1152/ajpheart.00484.2008), Teh et al 2017 (doi:10.1186/s12968-017-0342-x).
pg 9 line 1-17 You suggest that your new "long-axis myofiber layer" is important for electrical propagation in the heart, but you spend only a single sentence including the cardiac conduction system and the Purkinje fibers. Please indicate your long-axis layer would work with the cardiac conduction system, and whether your layer are specialized conduction cells.
pg 12 line 22-25 It is not clear which angles you are referring to, and unfortunately the diagram in Figure 2 is also not clear. Are you using projected helical angle, transverse angle and helical angle as in Agger et al 2020 Figure 2 (doi:10.1002/nbm.4205)? If so please refer to them using these names. For theta, when the cell long axis is projected onto the short-axis plane, are you calculating the angle from the radial or the circumferential direction?

Minor concerns:
pg 1 line 17 Cardiomyocytes often have a branching structure and do not form long exclusively end-to-end myofibers as in skeletal muscle.
pg 2 line 3 Cardiomyocytes often have a branching structure and do not form long exclusively end-to-end myofibers as in skeletal muscle.
pg 4 line 6 This is entirely expected of cutting cylinder-shaped cells at different angles - it seems strange to call it a "striking feature"
pg 4 line 13-14 "strong agreement between the two" I'd like to see a numerical result here. What was the mean/median

difference in orientation? What it centered on zero? Was there any bias?
pg 4 line 21-23 Why use three angles? Only two are needed. Helix angle and transverse angle OR helical angle and intrusion angle (Agger et al 2019, doi:10.1002/nbm.4205)
pg 4 line 24 Please use circumferential, longitudinal, radial (C, L, R) coordinates - the reader isn't particularly interested in the orientation you imaged the samples, more in the anatomical directions.
pg 5 line 1-3 Cardiomyocytes often have a branching structure and do not form long exclusively end-to-end myofibers as in skeletal muscle.
pg 5 line 24 "distinct bands of long-axis fibers" In my opinion you would need histology data that shows a clear membrane or separation of these epicardial cardiomyocytes to make this claim.
pg 7 line 15-16 I'm not convinced that there are three separate fiber/tract systems here. The apical section clearly has a triangular shape that may or may not be giving the impression of three groups. Even numerical analysis of these tracts will be impacted by the apparent size of the tracts and the kernel size used for the structure tensor analysis.
pg 7 line 24-25 "revealed an entirely new long-axis fiber system" Even Streeter et al 1969 (doi:10.1161/01.RES.24.3.339) showed a sharp transition of longitudinally oriented to circumferentially oriented cardiomyocytes. How is your finding different from this?
pg 7 line 27 "ventricular walls of four-chambered hearts" 6 mouse hearts and 1 rat heart is not sufficient data to make this claim.
Figure 1 It is very difficult to see the purple cylinders, perhaps because the yellow cylinders are so much longer than the purple ones.
Figure 2 I know the angle diagrams are difficult to construct, but the angle diagram is not clear, apart from the helix angle.
Figure 3 In b, make clear which sections each of the three solid lines represent from the 2 hearts. Also in the pseudo low resolution the main thing missing is the sharp but somewhat random transitions (green -65 to -90, blue -95 to -85, red -85 to -90), are you saying that these variable transitions indicate your long-axis fiber layer? I'm not sure what this pseudo low resolution data is showing me.

Edits:
pg 2 line 23 Referencing error - presumably they shouldn't be the same reference (18). They're also a different referencing style.
pg 3 line 23 Italicize "ex vivo"
pg 3 line 22-30 This belongs in the methods section, not the results section.
pg 3 line 32-34 This belongs in the methods section, not the results section.
pg 4 line 1-5 This belongs in the methods section, not the results section.
pg 4 line 9-11 This belongs in the methods section, not the results section.
pg 4 line 14-17 This belongs in the discussion section, not the results section.
pg 4 line 21-27 This belongs in the methods section, not the results section.
pg 4 line 27-32 This belongs in the discussion section, not the results section.
pg 4 line 32-33 This belongs in the methods section, not the results section.
pg 5 line 3-5 This belongs in the methods section, not the results section.
pg 5 line 5-7 This belongs in the discussion section, not the results section.
pg 5 line 10-12 This belongs in the methods section, not the results section.
pg 5 line 15-18 This belongs in the discussion section, not the results section.
pg 5 line 30-32 This belongs in the discussion section, not the results section.
pg 5 line 32-33 This belongs in the methods section, not the results section.
pg 6 line 1-2 This belongs in the methods section, not the results section.
pg 6 line 9-12 This belongs in the discussion section, not the results section.
pg 6 line 14-15 This belongs in the intro section, not the results section.
pg 6 line 15-18 This belongs in the methods section, not the results section.
pg 6 line 2 Please use "wedge sector" or simply "sector" rather than "pie-shaped sector".
pg 6 line 4 Please use anatomic directions such as anterior/posterior/septal/lateral/RV instead of "3 O'clock position"

Referee #3:

The paper describes a method to reconstruct myocardial fibers at micron-scale resolution. The topic is of interest for the clinical cardiac imaging community, in particular DT-MRI, since microscopy can provide ground truth orientation measurements. The proposed experimental protocol included tissue clearing to prepare the fresh excised mice hearts, slicing of short-axis and long-axis slabs, and confocal microscopy imaging. Image processing methods are used to register and assemble the image stacks, to mitigate blurring and noise, and to reconstruct fiber orientation using the structure tensor. The overall patterns of myofiber organization agree with the literature, however it is claimed that a new distinct fiber structure was found in the outer wall. The experiments and methods are clearly described. The image processing methods are sound and use state-of-the-art algorithms. I have some questions though, listed below.
Major comments
1) About the claim of a new fiber system found in the outer wall : isn't is the pericardium? It seems to me that some layers of pericardium contain fibrous structures. Would it be useful to indicate the different cell types on the raw images to make it clear?
2) Validation of the structure tensor calculations: 70 myocytes where manually selected for comparison, how where they

selected? Was it randomly, did the authors check on difficult cases, i.e. when the structure tensor is very different from the expected elongated cell type?
3) Other ways to visualize the orientation results could also be considered, inspired from DT-MRI: color-coded fractional anisotropy map is a nice way to summarize the fiber orientation in a single image. Fractional anisotropy may also help to check where the rank-1 structure tensor is not a good fit to the actual structure.
4) Figure 3 is difficult to read: it is not clear what the top graph is showing (missing legends in b, not obvious which region the zoomed plot corresponds to). Penetration depth should rather be call transmural depth (to avoid confusion with the light penetration depth problem mentioned in another part of the paper). In b, c, d, it could be indicated where is the inner/outer edge of the wall, i.e. left or right in the abscise axis? Also, I found the sharp changes indicated by the authors are clearly visible in the alphaH images, but not so clear on the transmural plots (maybe indicate them with an arrow).
5) I found the application of the patch-based denoising interesting, i.e. training the dictionary on the high-SNR data to denoise the lower-SNR data. Since the SNR varies with the z location in the slab, there might be a risk of a slight bias in the HA maps, due to residual noise being interpreted as structural gradient by the structure tensor. Did the authors consider that? As an alternative, would it be possible to acquire the images twice with flipped orientation of the sample?
Minor comments
6) Averaging of the structure tensor (section 3.6 of Supplementary material): why did the authors apply another filtering here, wouldn't it be simpler to increase the Gaussian kernel width/sigma already present in the structure tensor definition?
7) Maybe a point of discussion: apart from DT-MRI, phase-contrast CT has also been reported to achieve 3D myofiber reconstruction, and its resolution may be on the order of 10 microns. Since anyway, we are talking about cell orientation, going below the cell size is not strictly necessary. Actually with the Gaussian filter in the structure tensor definition (sigma=3 voxels) and the further averaging, the present study is working roughly at the same resolution. I think the main advantage is not so much the gain in resolution, but the more direct and clear visualization of cell boundaries which make the estimate more trustable and which can be validated by the manual contouring (this seems to me the most important part in the results).
8) Helix angle, in all color-coded images, is scaled between 0 and 90{degree sign}, whereas it should be between -90 and 90{degree sign} (as it is in the transmural plots). Could the authors make it consistent throughout the figures?
9) Fig 2e : there seems to be a problem with the aplhaH computation here, could the authors comment (although theta and phi maps seem correct)? Also it seems to be a slightly different slice and mask compared to c and d.

Detailed Response to Referee comments (EMBOJ-2022-113288R):

Referee #1 (Remarks to the Author):

In the manuscript of EMBOJ-2022-113288 entitled "Myofiber reconstruction at micron scale reveals long-axis fiber continuum in heart ventricular walls", Dr. Dileep and colleagues reported a prominent long-axis fiber system in the outer walls of the heart, distinct from the well-known helical myofibers and the longitudinal myofibers in the inner heart walls, by using microscopy and computer vision with 3D reconstruction of myofibers at the micron scale. This paper is well written and may address an important topic. Also, the quality of figure and video is good; however, it will be somewhat hard to understand, and there are some concern to be addressed.

1. The authors emphasized better understanding of myofiber geometry at the micron scale, compared with DT-MRI studies. Please discuss about it and clarify the clinical impact, rather than mentioning the association between pathological heart conditions and fiber organization.

2. This study is based on histological examination. In human study, only small samples from endomyocardial biopsy are obtained, while DT-MRI can provide noninvasively whole-heart assessment even as less spatial resolution.

Author Response:
We thank the reviewer for this summary of our methodology and findings and for appreciating our work. In the Discussion we have included mention of the clinical impact of our work and have also addressed the tradeoff between assessing myofiber geometry at the cellular scale versus at the whole-heart (typically millimeter) scale of other studies. Please refer to page 8, lines 31-33 and page 9, lines 1-15.

Minor comments

1.Can they show the images without deconvolution and denoising? Is there any difference in results if not using deconvolution or denoising?

Author Response:
We computed the orientation estimates and the corresponding helix angle for SAS3 data and show the comparison between raw data and the pre-processed images in Supplementary Figure 4c. The comparison shows that pre-processing (deconvolution + denoising) significantly improves the orientation estimation and calculation of angles.

2. Page 10, line 33 What do they mean as Figure 1a? Please modify the figure legend
Author Response:
We thank the reviewer for pointing out this mistake. We have corrected this error in the revised manuscript.

3. Page 3, line 26 Words such as PSAX-PML, HLA-4C need to be spelled out.
Author Response:
We have provided the expanded form of each of these acronyms in the revised manuscript. Please see Results, page 3, lines 31-32.

Referee #2 (Remarks to the Author):

Summary:
In this manuscript, Dileep et al apply the CLARITY tissue clearing process to mouse hearts (and one rat heart), apply a stain that allows visualization of cardiomyocyte boundaries (WGA). Sections of these hearts are imaged using confocal microscopy, and then processed using structure tensor analysis.
While this manuscript has some nice imaging data, in this reviewer's opinion importance of the novel "long-axis myofiber layer" is not supported by the data in the manuscript or the supplemental data.
Overall, I don't recommend this manuscript for publication in its current form. Larger sample sizes and additional imaging or other data - separate from the structure-tensor processed data - would be needed in order for it to be considered a genuine anatomical discovery. In this reviewer's opinion this manuscript would be better suited to focus on its excellent image processing and visualization.

Author Response:
We appreciate the concerns raised by the reviewer. We acknowledge the limitations of our work, and as the reviewer suggests, we now refer to the long-axis fibers as a geometrically distinct long-axis system, which is what our data and 3D reconstructions support. We appreciate the reviewer's positive comments about the image processing and visualization methods we have introduced.

Major concerns:
You refer to the "long-axis myofiber layer" "long-axis bands of fibers" "distinct from the known helical myofibers" "separate long-axis outer wall fiber system". Is there some sort of anatomic separation of this set of cardiomyocytes? Some sort of collagen structure or membrane? It seems that the distinctness of these cardiomyocytes from the rest of the myocardium is over-stated.

Author Response:
*We appreciate this concern. From our imaging data and analyses we have not observed a physical or anatomical separation between the long-axis fibers and the circumferential (helical) ones. However, Pope et. al. 2008 (*doi:10.1152/ajpheart.00484.2008*) have found evidence of a distinct organization of collagen in the outer wall region, suggesting that some level of anatomical separation in terms of the collagen matrix could occur. As stated above, we now refer to cardiomyocyte orientation and the recovered streamlines as a geometrically distinct system, throughout the revised manuscript. Please see page 1, lines 23-24, page 3, lines 16-23, page 6, lines 1-4, page 8, lines 12-15.*

The authors refer to "myofibers" throughout. The heart doesn't contain myofibers, and in this reviewer's opinion the term should no longer be accepted for anatomic descriptions of the heart. Within a large voxel (say 1x1x1 mm) there can be considered to be a "net cardiomyocyte orientation" or "aggregate cardiomyocyte orientation". If you're referring to structure tensor data, refer to the eigenvector directions. If you're referring to tract/streamline, refer to tract/streamline orientation.

Author Response:
We agree and thank the reviewer for this comment. Indeed, although the term `myofiber' has been widely used in the literature, fibers do not actually exist in a structural sense in the heart wall. We have modified our manuscript to explain, at the beginning, our use of the term 'fibers' to mean 'cardiomyocyte aggregates', and have also discussed the relationship to streamlines generated from cardiomyocyte orientation estimates. We have followed the reviewer's constructive suggestion to

refer to eigenvector based orientations or tract/streamline orientations or to use the term aggregate, when appropriate.

The text in the results section provides an insufficient summary of the experimental data. The majority of the text in the "results" section looks like it belongs in the methods or discussion section. Please refer to actual numerical results as much as possible, and avoid interpreting color maps. For me to consider a genuine "layer" of cells based on transmural helix angle I would want to see a clear discontinuity, which I don't see in your data.

Author Response:
We thank the reviewer for these suggestions. To present the data clearly we have made the following changes:

- a. *In Figure 3 we have improved the presentation of data to highlight the sharp changes. In addition, we have added rate of change of aH plots in Supplementary Figure 3d and 3e, to emphasize sharp changes.*
- b. *To present our findings quantitively we have incorporated the numerical angle values for φ, θ and aH in the Results section.*
- c. *We have also revised the Results and Discussion sections to minimize overlap with the Methods section. Please see page 3, lines 27-34.*

The abstract should be much more specific. What breed of mouse? How many mice? What tissue clearing process? How were the images processed? What is the most important helix angle data?

Author Response:
The current abstract is in line with The EMBO Journal guidelines. We have revised the abstract based on all the reviewer suggestions and the editor's recommendation to place more emphasis on the methods contribution of the article.

pg 2 line 30-34 & pg 3 line 1-5 This paragraph is essentially wrong because of the Garcia-Canadilla et al 2019 paper (doi:10.1111/joa.13058), which you later misreport as using DT-MRI (pg10 line 12). Also, Pope et al 2008 (doi:10.1152/ajpheart.00484.2008), Teh et al 2017 (doi:10.1186/s12968-017-0342-x).

Author Response:
We thank the reviewer for pointing out this error. We have corrected these mistakes. Please see page 2, line 34, page 3 lines 1-4.

pg 5 line 19-23 This would likely look different depending which colormap you used. You should stick to reporting angle numbers rather than colors from color maps.

Author Response:
We agree with the reviewer and have added quantitative descriptions in the revised article as much as possible in the Results section. See page 5, lines 30-33.

pg 6 line 12-14 "entirely new discovery" To me the epicardial structures you're reporting look the same as those reported by Teh et al 2016 (doi:10.1016/j.pbiomolbio.2016.06.001) Figures 3 and 7, and probably others. Why do you think the steep transition in epicardial helix angle is not observed in Pope et al 2008 (doi:10.1152/ajpheart.00484.2008) which also has micron scale data?

Author Response:
The epicardial structures reported in Teh et al. 2016 are at the millimetre voxel resolution. Figure 3 of Teh et al. 2016 shows orientation glyphs in a short-axis view, where a sharp change in αH at the outer wall is not evident. Figure 8 in this article shows αH plots, where again no sharp changes are seen. The article does not describe a geometrically distinct long-axis epicardial system.

The Pope et al. 2008 article reports collagen organization across rat ventricular walls, but does not show αH analyses. However, this article has reported a distinct collagen orientation in the sub-epicardial regions. This finding provides indirect support for our findings of geometrically distinct cardiomyocyte arrangements in the outer walls. We have added this point in the Discussion (page 9, lines 8-12).

pg 6 18-21 You need to be much more descriptive in what Figure 3b is showing, and what resolutions the different colored lines are referring to, dotted versus solid etc.

Author Response:
We thank the reviewer for this comment. We have improved the presentation and discussion of Figure 3.

pg 6 line 26-29 How confident are you that these are not just papillary muscles?

Author Response:
Papillary muscles are seen in the cavity of the ventricles and are attached to their walls. The geometrically distinct long-axis cardiomyocyte layer we refer to is observed in the outer ventricular walls and is quite distinct from these.

pg 8 line 9-11 "a resolution gain of about 3 orders of magnitude". I don't think this is correct. Please re-check Garcia-Canadilla et al 2019 (doi:10.1111/joa.13058), Pope et al 2008 (doi:10.1152/ajpheart.00484.2008), Teh et al 2017 (doi:10.1186/s12968-017-0342- x).

Author Response:
Garcia-Canadilla et al. 2019 use high resolution episcopic microscopy to image a thin section of the postnatal mouse heart, infiltrated with methacrylate resin. They report an XY resolution of ~5.5 um and 3 um Z slice thickness. The voxel size used by Garcia-Canadilla is therefore ~164 um^3. Thus their study is at a coarser resolution than ours.
Pope et al. 2008 focuses on the organization of collagen in the extracellular matrix in wild type rat hearts at an isotropic resolution of 1 um. Further, they only image a limited heart wall region and not the entire heart section. Teh et al. 2017 use an isotropic voxel of size 7.2 um. Both these studies do not use staining specific to cell membranes in the heart and thus cannot provide data to infer cardiomyocyte boundaries or fiber orientation. We have included this point in the Discussion (page 9, lines 4-12).

pg 9 line 1-17 You suggest that your new "long-axis myofiber layer" is important for electrical propagation in the heart, but you spend only a single sentence including the cardiac conduction system and the Purkinje fibers. Please indicate your long-axis layer would work with the cardiac conduction system, and whether your layer are specialized conduction cells.

Author Response:

This was a speculative discussion point, drawing a connection to past work (e.g., the connection to conduction in the long-axis epicardial system in the Circulation paper by Vetter et al.). We have not determined whether the cells in the long-axis system are specialized conduction cells. Thus, the question of the role of a geometrically distinct long axis system for conduction remains a topic for future investigation. We have expanded this point in the Discussion (page 10, lines 22-33).

pg 12 line 22-25 It is not clear which angles you are referring to, and unfortunately the diagram in Figure 2 is also not clear. Are you using projected helical angle, transverse angle and helical angle as in Agger et al 2020 Figure 2 (doi:10.1002/nbm.4205)? If so please refer to them using these names. For theta, when the cell long axis is projected onto the short-axis plane, are you calculating the angle from the radial or the circumferential direction?

Author Response:
We thank the reviewer for pointing this out. We have improved the illustration in Figure 2a. We have also cited Agger et al. 2020 for the helix angle we are referring to.

Minor concerns:
pg 1 line 17 Cardiomyocytes often have a branching structure and do not form long exclusively end-to-end myofibers as in skeletal muscle.
pg 2 line 3 Cardiomyocytes often have a branching structure and do not form long exclusively end-to-end myofibers as in skeletal muscle.

Author Response:
In the revised manuscript we have addressed this by referring to the aggregate cardiomyocyte orientation, following the reviewer's earlier suggestion.

pg 4 line 6 This is entirely expected of cutting cylinder-shaped cells at different angles - it seems strange to call it a "striking feature"

Author Response:
We have modified this sentence to clarify what we meant (page 4, lines 12-13).

pg 4 line 13-14 "strong agreement between the two" I'd like to see a numerical result here. What was the mean/median difference in orientation? What it centered on zero? Was there any bias?

Author Response:
We thank the reviewer for this comment and the opportunity to clarify this point. We have examined the absolute value of the difference between hand segmented and quantitatively estimated cardiomyocyte orientations. We have included the numerical mean/median absolute difference in orientation in the revised manuscript (page 4, lines 19-24).

pg 4 line 21-23 Why use three angles? Only two are needed. Helix angle and transverse angle OR helical angle and intrusion angle (Agger et al 2019, doi:10.1002/nbm.4205)

Author Response:
This is true, but it is not straightforward to robustly compute the helix angle αH in the long-axis sections since an estimate of the transmural penetration direction is required for it. We therefore chose the magnitude of the φ angle to highlight the long-axis orientation of outer wall cells in a

short-axis section (Figure 2d). We have also improved our presentation of definitions of angles and their illustration in Figure 2a.

pg 4 line 24 Please use circumferential, longitudinal, radial (C, L, R) coordinates - the reader isn't particularly interested in the orientation you imaged the samples, more in the anatomical directions.

Author Response:
We have included the C, L, R coordinate frame in our illustration of the helix angle. Please see Figure 2a.

pg 5 line 24 "distinct bands of long-axis fibers" In my opinion you would need histology data that shows a clear membrane or separation of these epicardial cardiomyocytes to make this claim.

Author Response:
This concern is similar to that raised by the reviewer earlier. We have addressed this point and have revised the manuscript accordingly to refer to a geometrically distinct long-axis system. Please see page 1, line 23-24, page 3, lines 16-23, page 6, lines 1-4, page 8, lines 12-17.

pg 7 line 15-16 I'm not convinced that there are three separate fiber/tract systems here. The apical section clearly has a triangular shape that may or may not be giving the impression of three groups. Even numerical analysis of these tracts will be impacted by the apparent size of the tracts and the kernel size used for the structure tensor analysis. pg 7 line 24-25 "revealed an entirely new long-axis fiber system" Even Streeter et al 1969 (doi:10.1161/01.RES.24.3.339) showed a sharp transition of longitudinally oriented to circumferentially oriented cardiomyocytes. How is your finding different from this?

Author Response:
The Streeter et al. 1969 article shows the presence of longitudinal fibers in the endocardium and epicardium, similar to Green et al. 1981. However, both these reports do not comment on the fiber continuum and neither do several follow up studies. This marks the major difference between our work and previous studies. We describe a long-axis fiber continuum in the outer and inner ventricular walls. In Figure 6, we have shown different apical sections from the apex to the base of the heart. At the bottommost apex section we see three geometrically distinct aggregate cardiomyocyte orientations converge at what appears to be a singularity. We agree that these are qualitative observations. In the above sections (towards the base), these aggregate orientation bands emerge as separate units in the LV wall. We have revised Figure 6 to include the apex data as a series of sections from apex to base, which should clarify our claims.

pg 7 line 27 "ventricular walls of four-chambered hearts" 6 mouse hearts and 1 rat heart is not sufficient data to make this claim.

Author Response:
We have changed our wording and have softened claims that are not substantiated by data. Please see page 8, lines 15-17.

Figure 1 It is very difficult to see the purple cylinders, perhaps because the yellow cylinders are so much longer than the purple ones.

Author Response:
We thank the reviewer for pointing this out. We have provided an improved version of Figure 1 in the revised manuscript.

Figure 2 I know the angle diagrams are difficult to construct, but the angle diagram is not clear, apart from the helix angle.

We thank the reviewer for pointing out this. We have provided an improved version of Figure 2, illustrating the angles we have used, in the revised manuscript.

Figure 3 In b, make clear which sections each of the three solid lines represent from the 2 hearts. Also in the pseudo low resolution the main thing missing is the sharp but somewhat random transitions (green -65 to -90, blue -95 to -85, red -85 to -90), are you saying that these variable transitions indicate your long-axis fiber layer? I'm not sure what this pseudo low resolution data is showing me.

Author Response:
We have modified Figure 3 thoroughly to accommodate the suggested changes. We also have included the description and findings from the pseudo low resolution analysis in the Results section (page 6, lines 28-33).

Edits:

Author Response:
We thank the reviewer for all the suggested edits listed below.

pg 2 line 23 Referencing error - presumably they shouldn't be the same reference (18). They're also a different referencing style.
We have fixed this referencing issue in the revised article.

pg 3 line 23 Italicize "ex vivo"
We have italicized this word in the revised article. Please see page 3, line 28.

pg 3 line 22-30 This belongs in the methods section, not the results section.
pg 3 line 32-34 This belongs in the methods section, not the results section.
pg 4 line 1-5 This belongs in the methods section, not the results section.
pg 4 line 9-11 This belongs in the methods section, not the results section.
pg 4 line 14-17 This belongs in the discussion section, not the results section.
pg 4 line 21-27 This belongs in the methods section, not the results section.
pg 4 line 27-32 This belongs in the discussion section, not the results section.
pg 4 line 32-33 This belongs in the methods section, not the results section.
pg 5 line 3-5 This belongs in the methods section, not the results section.
pg 5 line 5-7 This belongs in the discussion section, not the results section.
pg 5 line 10-12 This belongs in the methods section, not the results section.
pg 5 line 15-18 This belongs in the discussion section, not the results section.
pg 5 line 30-32 This belongs in the discussion section, not the results section.
pg 5 line 32-33 This belongs in the methods section, not the results section.
pg 6 line 1-2 This belongs in the methods section, not the results section.
pg 6 line 9-12 This belongs in the discussion section, not the results section.
pg 6 line 14-15 This belongs in the intro section, not the results section.

pg 6 line 15-18 This belongs in the methods section, not the results section.
pg 6 line 2 Please use "wedge sector" or simply "sector" rather than "pie-shaped sector".
pg 6 line 4 Please use anatomic directions such as anterior/posterior/septal/lateral/RV instead of "3 O'clock position"

Author Response:
We have changed the wording to" wedge shaped sector" or "sector" and have included the anatomic directions both in Figure 3 and the Results. Please refer to page 6, lines 13-20. Similarly we have modified the manuscript to accommodate all the reviewers' editorial suggestions and The EMBO Journal requirements.

Referee #3 (Remarks to the Author):

The paper describes a method to reconstruct myocardial fibers at micron-scale resolution. The topic is of interest for the clinical cardiac imaging community, in particular DT-MRI, since microscopy can provide ground truth orientation measurements. The proposed experimental protocol included tissue clearing to prepare the fresh excised mice hearts, slicing of short-axis and long-axis slabs, and confocal microscopy imaging. Image processing methods are used to register and assemble the image stacks, to mitigate blurring and noise, and to reconstruct fiber orientation using the structure tensor. The overall patterns of myofiber organization agree with the literature, however it is claimed that a new distinct fiber structure was found in the outer wall. The experiments and methods are clearly described. The image processing methods are sound and use state-of-the-art algorithms. I have some questions though, listed below.

Major comments
1) About the claim of a new fiber system found in the outer wall:isn't it the pericardium? It seems to me that some layers of pericardium contain fibrous structures. Would it be useful to indicate the different cell types on the raw images to make it clear?

Author Response:

We thank the reviewer for the opportunity to elaborate on this point. The outer wall long-axis fibers described in the manuscript are not merely fibrous structures but rather are stacked end-on-end cardiomyocytes forming an entire distinct geometrical system (seen in our short- and long-axis heart sections). The dimensions of the cardiomyocytes (diameter in a short-axis-section and length in a long-axis-section) in the outer wall are within a range that is expected of a typical cardiomyocyte. Our confocal images are also undersampled so that we acquire isotropic data, at 2-micron voxel resolution, which cannot resolve other small non-cardiomyocyte cells (please see Supplementary Figure 1f). We have added text about this in the Results section (page 4, lines 4-6).

In addition, in the figure below, we provide evidence for the reviewer that the outer wall cells we refer to are indeed cardiomyocytes, as shown by the presence of sarcomeric structures with the help of alpha-actinin staining. We are confident that the outer wall cells we report are indeed cardiomyocytes that form a long-axis system.

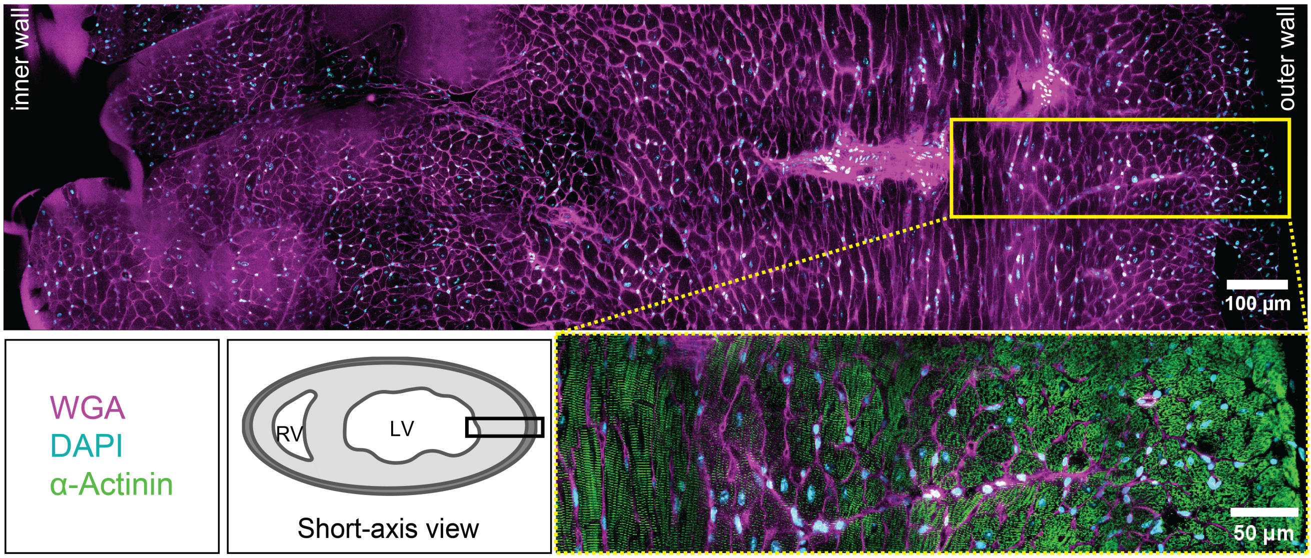

A representative image highlighting the cardiomyocytes stained using WGA, DAPI and alpha actinin to mark the cell membranes, nuclei and Z-disc regions of sarcomeres, respectively. The images were acquired with a 60X objective using a confocal microscope. The LV-wall region marked with a black box in the schematic representation of the short axis section (bottom middle) is shown in the top panel. A zoomed in region (yellow box) showing the middle wall to outer wall area is shown in the bottom right panel. The cardiomyocytes in the outer wall pick up staining for alpha actinin, confirming the presence of cardiac muscle cells. The scale bars are as indicated.

2) Validation of the structure tensor calculations: 70 myocytes where manually selected for comparison, how where they selected? Was it randomly, did the authors check on difficult cases, i.e. when the structure tensor is very different from the expected elongated cell type?

Author Response:
We thank the reviewer for this query and for the opportunity to clarify this point. For validation we handpicked 70 myocytes from a randomly selected 3D image stack (field of view) but we did not bias the selection of cell types in any particular way. We have elaborated on this in the Detailed Methods (Section 3.7). In our study, we have data from only cardiomyocytes, which are elongated cells. To validate our structure tensor calculations, we have now also calculated the Fractional Anisotropy (FA) scores based on structure tensor eigenvalues, for the entire short-axis section and have found that the majority of the voxels have high FA scores. We have illustrated this using a colormap and an FA distribution plot in Supplementary Figure 3a. These analyses reveal that the structure tensor indeed consistently reveals an elongated cell type.

3) Other ways to visualize the orientation results could also be considered, inspired from DT-MRI: color-coded fractional anisotropy map is a nice way to summarize the fiber orientation in a single image. Fractional anisotropy may also help to check where the rank-1 structure tensor is not a good fit to the actual structure.

Author Response:
We thank the reviewer for this suggestion. We have provided calculations of and visualizations of the FA map using eigenvalues of the structure tensor based estimates in the revised article (Supplementary Figure 3a). The maps reveal a high FA score at most locations in the short-axis section.

4) Figure 3 is difficult to read: it is not clear what the top graph is showing (missing legends in b, not obvious which region the zoomed plot corresponds to). Penetration depth should rather be call transmural depth (to avoid confusion with the light penetration depth problem mentioned in another part of the paper). In b, c, d, it could be indicated where is the inner/outer edge of the wall, i.e. left or right in the abscise axis? Also, I found the sharp changes indicated by the authors are clearly visible in the alphaH images, but not so clear on the transmural plots (maybe indicate them with an arrow).

Author Response:
We thank the reviewer for pointing this out, and we entirely agree. We have now included the suggested changes to Figure 3 in the revised manuscript. We have also included the rate of change of the Helix angle (αH) in Supplementary Figure 3d, along with the transmural penetration, to highlight the sharp change in orientation near the outer boundary of the LV.

5) I found the application of the patch-based denoising interesting, i.e. training the dictionary on the high-SNR data to denoise the lower-SNR data. Since the SNR varies with the z location in the slab, there might be a risk of a slight bias in the HA maps, due to residual noise being interpreted as structural gradient by the structure tensor. Did the authors consider that? As an alternative, would it be possible to acquire the images twice with flipped orientation of the sample?

Author Response:
We thank the reviewer for this valuable suggestion. We have in fact we tried the idea of imaging from both sides of the section. However, when flipped the tissue sample also bends (due to gravity), so the precise registration of the flipped sample with the original becomes an issue. A non-linear registration method would have to be applied between the original and the flipped sample, while preserving and aligning cardiomyocyte boundaries. This is a matter for future investigation and if addressed a solution could apply to other confocal microscopy based tissue imaging problems as well. Regarding the concern about noise biasing our αH maps, our structure tensor based orientation is estimated use the intensity gradient between neighbouring voxels with an initial smoothing to ensure that the gradient calculations were valid. As such, we did not notice biases due to variations in contrast. In addition, our pre-processing pipeline (deconvolution and denoising) helped to improve SNR at the deeper layers, as evident from the raw versus pre-processed comparisons shown in Supplementary Figure 4c.

Minor comments

6) Averaging of the structure tensor (section 3.6 of Supplementary material): why did the authors apply another filtering here, wouldn't it be simpler to increase the Gaussian kernel width/sigma already present in the structure tensor definition?

Author Response:
This is an interesting suggestion. We decided to proceed in two steps, since we had an idea of the expected size of a myocyte. Our first smoothing scale is to ensure that gradient calculations to build the elements of the structure tensor are valid. We then selected the object scale (size of neighborhood) for the structure tensor according to the expected size of a typical myocyte. The choice of parameters of the structure tensor were therefore guided by the resolution of imaging. The averaging of the estimated orientations was carried out in a separate step, to allow us to also study the impact of acquiring data at coarser resolutions. While we didn't vary the object scale for the

structure tensor as suggested by the reviewer, we expect similar results to our orientation smoothing (averaging) when using a larger object scale.

7) Maybe a point of discussion: apart from DT-MRI, phase-contrast CT has also been reported to achieve 3D myofiber reconstruction, and its resolution may be on the order of 10 microns. Since anyway, we are talking about cell orientation, going below the cell size is not strictly necessary. Actually with the Gaussian filter in the structure tensor definition (sigma=3 voxels) and the further averaging, the present study is working roughly at the same resolution. I think the main advantage is not so much the gain in resolution, but the more direct and clear visualization of cell boundaries which make the estimate more trustable and which can be validated by the manual contouring (this seems to me the most important part in the results).

Author Response:
We agree with the reviewer that the estimates of orientation by extracting information from cell boundary labelling is indeed more trustworthy. Even though CT imaging provides micron resolution, the images lack features to easily demarcate the boundaries of cells. We have included this comparison in the Discussion section. Please see page 9, lines 4-15.

8) Helix angle, in all color-coded images, is scaled between 0 and 90{degree sign}, whereas it should be between -90 and 90{degree sign} (as it is in the transmural plots). Could the authors make it consistent throughout the figures?

Author Response:
We thank the reviewer for this excellent suggestion. We have recomputed all color-coded images which show the Helix angle to now use a -90 (blue) to +90 (yellow) color scale, as is also consistent with the transmural plots.

9) Fig 2e : there seems to be a problem with the alphaH computation here, could the authors comment (although theta and phi maps seem correct)? Also it seems to be a slightly different slice and mask compared to c and d.

Author Response:
We thank the reviewer for raising this concern. In fact, the θ and φ maps are calculated in 2D planes, as shown in Figure 2c and d. But the α_H maps are computed as 3D averages per stack/ tissue section, and this is the reason the masking appears different. We have improved the article to address this issue related to color coding and slice masks, by referring to φ and θ colormaps as a 'single plane representation' (page 5, lines 22-24) and the α_H colormaps as '3D average values' (page 6, lines 13-14).

Dear Minhaj,

Congratulations on a great revision! Overall, two of the referees are satisfied with your revision. However referee 2 remains unconvinced that the data shown fully supports a new long-axis band. Therefore, to move forward with acceptance, we would require you to address the remaining major concern as suggested by referee 2. This can be done with the experiments/statistics recommended or by modifying the claim and including in the discussion alternative theories that could explain the data, as proposed by the referee.

When you submit your revised version, please also take care of the following editorial items and add this also to your point-by-point response:

1. Please include the following funding info in eJP online: National Mouse Research Resource grant (BT/PR5981/MED/31/181/2012;2013-2016 & 102/IFD/SAN/5003/2017-2018) ; EMBO Young Investigator Programme award, CEFIPRA (5703-1) from the Department of Science and Technology; Rajiv Gandhi University of Health Sciences (RGUHS), Scientist Development Grant (15SDG23250005) from the American Heart Association (AHA), a Department of Science and Technology Grant (DST/CRG/2019/005401) and inStem core funding; Natural Sciences and Engineering Research Council of Canada (NSERC) and to the Fonds de Recherche du Québec Nature et Technologies (FRQNT); CSIR-SRF

2. Please include up to five keywords, which may or may not appear in the title, should be given in alphabetical order, below the abstract, each separated by a slash (/).

3. Please review our new policy on conflict of interests on the EMBO author guide website and update the title of this section to: Disclosure and competing interests statement

4. Please remove the author contribution section from the main manuscript

5. Each figure should be referred to/called out in the main manuscript in chronological order. The following callouts are missing: Fig. 1b, 4a-d, 5a-b, Supplementary Figure 2a-e, Supplementary Figure 3e, Supplementary Figure 4a-b, Supplementary Figure 5a-d, Supplementary Figure 7a-c; there is a callout for Supplementary Figure 4d but no such panel

6. The figures should be uploaded individually, and figure legends should be included in the manuscript file.

7. Please include a table of contents with page numbers for the appendix file and please update the nomenclature to Appendix Figure S1-S7, and Appendix Table S1, including updating the reference to these in the main manuscript

8. We include a synopsis of the paper (see http://emboj.embopress.org/). Please provide me with a general summary statement and 3-5 bullet points that capture the key findings of the paper.

9. We also need a summary figure for the synopsis. The size should be 550 wide by 200-440 high (pixels). You can also use something from the figures if that is easier.

10. Please refer to our website and correct the order of sections

11. Please explain the file "Dileep-Syed-etal-Detailed-methods". Is this EV content? The figures in this file have not been referenced in the main manuscript. References in this file should be alphabetical and 10 authors before et al. Movie files should be remained to Movie EV1-EV2 with corresponding call outs and the legends should be removed and zipped with each movie file.

Thank you for the opportunity to consider your work for publication. I look forward to your revision.

Kind regards,
Kelly

Kelly M Anderson, PhD
Editor
The EMBO Journal
k.anderson@embojournal.org

Further information is available in our Guide For Authors: https://www.embopress.org/page/journal/14602075/authorguide

Use the link below to submit your revision:

https://emboj.msubmit.net/cgi-bin/main.plex

Referee #2:

In this manuscript the authors use tissue clearing, microscopy and structure tensor analysis to analyze cardiomyocyte orientation in one long axis section and one short axis section of mouse hearts. Unfortunately in this reviewer's opinion the claim of discovery of a new long-axis band of cardiomyocytes is not supported by the data.

Major concern:
- The claim of a "geometrically distinct arrangement" is simply not supported by the data in the manuscript. The closest thing to evidence is the transmural helix angle traces in Fig 3. The steeper helix angle at the epicardium MIGHT be indicative of something, but it also might not be anything. You need to use statistical tests and multiple samples to support your claim. Is the change in helix angle steeper than in the midwall (statistically significant?), is the transition steeper in wedge sections that have the distinct region versus the wedges that don't? (statistically significant?) Can you prove that your result isn't simply an arcsine transmural trend as explained in Wilson et al 2023 (doi:10.1007/978-3-031-35302-4_7)?

Minor concerns
- The authors stand by their "three orders of magnitude" gain, but now say it is a gain over existing "models", rather than existing myocardial imaging data? I don't see a presentation of a new model in this manuscript, which in my mind would require some anatomic/schematic type diagram with explanation, or a computational model with definition of cardiomyocyte orientation over the whole heart. There is not a three order of magnitude gain over previous cardiac imaging data, so just leave this out. Why not focus on the positive aspects? To my knowledge you are the first to use tissue clearing to perform cardiomyocyte structural analysis over a long axis and short axis section. Why not just say that?

- I reiterate from my first review that you should focus on your strengths - the excellent microscopy and image analysis - rather than this possible long-axis band.

Referee #3:

The authors replied satisfactorily to all my comments. In particular they have clarified the claims regarding the distinct epicardial structure, and they improved the figures, especially Fig 3 which I find most important. I have no more questions and I think the paper could be accepted.

Editorial point-by-point response:

1. Please include the following funding info in eJP online: National Mouse Research Resource grant (BT/PR5981/MED/31/181/2012;2013-2016 & 102/IFD/SAN/5003/2017-2018) ; EMBO Young Investigator Programme award, CEFIPRA (5703-1) from the Department of Science and Technology; Rajiv Gandhi University of Health Sciences (RGUHS), Scientist Development Grant (15SDG23250005) from the American Heart Association (AHA), a Department of Science and Technology Grant (DST/CRG/2019/005401) and inStem core funding; Natural Sciences and Engineering Research Council of Canada (NSERC) and to the Fonds de Recherche du Québec Nature et Technologies (FRQNT); CSIR-SRF

We have included the funding information in the online submission.

2. Please include up to five keywords, which may or may not appear in the title, should be given in alphabetical order, below the abstract, each separated by a slash (/).

We have included the keywords in the revised manuscript.

3. Please review our new policy on conflict of interests on the EMBO author guide website and update the title of this section to: Disclosure and competing interests statement

We have changed this title in the revised manuscript.

4. Please remove the author contribution section from the main manuscript

We have removed this section from the revised manuscript.

5. Each figure should be referred to/called out in the main manuscript in chronological order. The following callouts are missing: Fig. 1b, 4a-d, 5a-b, Supplementary Figure 2a-e, Supplementary Figure 3e, Supplementary Figure 4a-b, Supplementary Figure 5a-d, Supplementary Figure 7a-c; there is a callout for Supplementary Figure 4d but no such panel

We have removed missing callouts and checked the figure callout in the revised manuscript.

6. The figures should be uploaded individually, and figure legends should be included in the manuscript file.

We have uploaded individual Adobe Illustrator files for each figure using the required format and figure legends have been added in the respective section of the revised manuscript.

7. Please include a table of contents with page numbers for the appendix file and please update the nomenclature to Appendix Figure S1-S7, and Appendix Table S1, including updating the reference to these in the main manuscript.

We have renamed “Dileep-Syed-etal-Detailed-methods” to “Appendix” and the figures in this section to ‘Appendix Figure S1’, and so on. We have also included a short table of contents at the beginning of the “Appendix” document.

8. We include a synopsis of the paper (see http://emboj.embopress.org/). Please provide me with a general summary statement and 3-5 bullet points that capture the key findings of the paper.

9. We also need a summary figure for the synopsis. The size should be 550 wide by 200-440(210 now) high (pixels). You can also use something from the figures if that is easier.

We have attached a word document file for the summary text and the 3-5 bullet point summary and have provided a separate AI file for the Synopsis image.

10. Please refer to our website and correct the order of sections

We have followed the correct order of sections in the revised manuscript.

11. Please explain the file "Dileep-Syed-etal-Detailed-methods". Is this EV content? The figures in this file have not been referenced in the main manuscript. References in this file should be alphabetical and 10 authors before et al. Movie files should be remained to Movie EV1-EV2 with corresponding call outs and the legends should be removed and zipped with each movie file.

Please see our response in point no. 7. We have corrected the naming of the movie files.

Referee #2:

In this manuscript the authors use tissue clearing, microscopy and structure tensor analysis to analyze cardiomyocyte orientation in one long axis section and one short axis section of mouse hearts. Unfortunately in this reviewer's opinion the claim of discovery of a new long-axis band of cardiomyocytes is not supported by the data.

Major concern:
- The claim of a "geometrically distinct arrangement" is simply not supported by the data in the manuscript. The closest thing to evidence is the transmural helix angle traces in Fig 3. The steeper helix angle at the epicardium MIGHT be indicative of something, but it also might not be anything. You need to use statistical tests and multiple samples to support your claim. Is the change in helix angle steeper than in the midwall (statistically significant?), is the transition steeper in wedge sections that have the distinct region versus the wedges that don't? (statistically significant?) Can you prove that your result isn't simply an arcsine transmural trend as explained in Wilson et al 2023 (doi:10.1007/978-3-031-35302-4_7)?

Our claim is based on geometric observation. Our orientation plots of static samples do not yet provide conclusive evidence of a distinct band of cells. However, based on our analysis of fixed samples, it is clear that the Helix Angle does not vary uniformly across the heart wall. In fact, as the reviewer points out, the very recent Wilson et. al. article (FIMH, June 2023) also reports a non-uniform variation of the helix angle in transmural penetrations in different regions of the heart wall. Although that study is at the millimeter scale in porcine hearts, which is 1000 times coarser in spatial resolution than our analysis, the article shows that in the left ventricular region an arcsine function, with a sharper rate of change in the epicardial region, is a better fit to DTI orientation measurements than linear or arctan functions. The tractography analysis in the article also demonstrates a clustering of streamlines in the epicardial and mesocardial regions. We have added a sentence about this in the discussion section along with citation to the recent paper Wilson et al 2023 (Please see Page 10 Lines 15-17). We agree with the reviewer that more statistical evidence and data samples would strengthen claims of the distinctiveness of the longitudinal bands we have reconstructed. We have therefore removed the phrase “geometrically distinct” throughout the article and have reworded the associated sentences. We have also included a reference to the very recent Wilson et al. conference paper.

Minor concerns
- The authors stand by their "three orders of magnitude" gain, but now say it is a gain over existing "models", rather than existing myocardial imaging data? I don't see a presentation of a new model in this manuscript, which in my mind would require some anatomic/schematic type diagram with explanation, or a computational model with definition of cardiomyocyte orientation over the whole heart. There is not a three order of magnitude gain over previous cardiac imaging data, so just leave this out. Why not focus on the positive aspects? To my knowledge you are the first to use tissue clearing to perform cardiomyocyte structural analysis over a long axis and short axis section. Why not just say that?

- I reiterate from my first review that you should focus on your strengths - the excellent microscopy and image analysis - rather than this possible long-axis band.

We thank the reviewer for bringing this concern to our attention. We agree and therefore have removed the word “models” from the abstract. In the discussion we have clarified that the gain in resolution is from the millimeter to the micron scale, for cardiomyocyte aggregate orientation recovery and for our 3D reconstructions. In the same section we have reviewed the limitations of previous studies (Page 8, line 28 to page 9 line 6).

Referee #3:

The authors replied satisfactorily to all my comments. In particular they have clarified the claims

regarding the distinct epicardial structure, and they improved the figures, especially Fig 3 which I find most important. I have no more questions and I think the paper could be accepted.

We thank the reviewer for their positive feedback and for their suggestions for improving the manuscript.

2nd Revision - Editorial Decision 6th Aug 2023

Dear Minhaj,

Congratulations on an excellent manuscript, I am pleased to inform you that your manuscript has been accepted for publication in The EMBO Journal. Thank you for your comprehensive response to the referee concerns, it has been a pleasure to work with you to get this to the acceptance stage.

I will begin the final checks on your manuscript before submitting to the publisher next week. Once at the publisher, it will take about 3 weeks for your manuscript to be published online. As a reminder, the entire review process, including referee concerns and your point-by-point response, will be available to readers.

I will be in touch throughout the final editorial process until publication. In the meantime, I hope you find time to celebrate!

Kind regards,
Kelly

Kelly M Anderson, PhD
Editor, The EMBO Journal
k.anderson@embojournal.org

Please note that it is EMBO Journal policy for the transcript of the editorial process (containing referee reports and your response letter) to be published as an online supplement to each paper. If you do NOT want this, you will need to inform the Editorial Office via email immediately. More information is available here:
https://www.embopress.org/page/journal/14602075/authorguide#transparentprocess

Your manuscript will be processed for publication in the journal by EMBO Press. Manuscripts in the PDF and electronic editions of The EMBO Journal will be copy edited, and you will be provided with page proofs prior to publication. Please note that supplementary information is not included in the proofs.

You will be contacted by Wiley Author Services to complete licensing and payment information. The required 'Page Charges Authorization Form' is available here: https://www.embopress.org/pb-assets/embo-site/tej_apc.pdf - please download and complete the form and return to embopressproduction@wiley.com

EMBO Press participates in many Publish and Read agreements that allow authors to publish Open Access with reduced/no publication charges. Check your eligibility: https://authorservices.wiley.com/author-resources/Journal-Authors/open-access/affiliation-policies-payments/index.html

Should you be planning a Press Release on your article, please get in contact with embojournal@wiley.com as early as possible, in order to coordinate publication and release dates.

If you have any questions, please do not hesitate to call or email the Editorial Office. Thank you for your contribution to The EMBO Journal.

** Click here to be directed to your login page: https://emboj.msubmit.net

EMBO Press Author Checklist

Corresponding Author Name: Kaleem Siddiqi and Minhajuddin Sirajuddin
Journal Submitted to: The EMBO Journal
Manuscript Number: EMBOJ-2022-113288R

USEFUL LINKS FOR COMPLETING THIS FORM
The EMBO Journal - Author Guidelines
EMBO Reports - Author Guidelines
Molecular Systems Biology - Author Guidelines
EMBO Molecular Medicine - Author Guidelines

Reporting Checklist for Life Science Articles (updated January
This checklist is adapted from Materials Design Analysis Reporting (MDAR) Checklist for Authors. MDAR establishes a minimum set of requirements in transparent reporting in the life sciences (see Statement of Task: 10.31222/osf.io/9sm4x). Please follow the journal's guidelines in preparing your
Please note that a copy of this checklist will be published alongside your article.

Abridged guidelines for figures
1. Data
The data shown in figures should satisfy the following conditions:
- the data were obtained and processed according to the field's best practice and are presented to reflect the results of the experiments in an accurate and unbiased manner.
- ideally, figure panels should include only measurements that are directly comparable to each other and obtained with the same assay.
- plots include clearly labeled error bars for independent experiments and sample sizes. Unless justified, error bars should not be shown for technical
- if n<5, the individual data points from each experiment should be plotted. Any statistical test employed should be justified.
- Source Data should be included to report the data underlying figures according to the guidelines set out in the authorship guidelines on Data

2. Captions
Each figure caption should contain the following information, for each panel where they are relevant:
- a specification of the experimental system investigated (eg cell line, species name).
- the assay(s) and method(s) used to carry out the reported observations and measurements.
- an explicit mention of the biological and chemical entity(ies) that are being measured.
- an explicit mention of the biological and chemical entity(ies) that are altered/varied/perturbed in a controlled manner.
- the exact sample size (n) for each experimental group/condition, given as a number, not a range;
- a description of the sample collection allowing the reader to understand whether the samples represent technical or biological replicates (including how many animals, litters, cultures, etc.).
- a statement of how many times the experiment shown was independently replicated in the laboratory.
- definitions of statistical methods and measures:
  - common tests, such as t-test (please specify whether paired vs. unpaired), simple χ2 tests, Wilcoxon and Mann-Whitney tests, can be unambiguously identified by name only, but more complex techniques should be described in the methods section;
  - are tests one-sided or two-sided?
  - are there adjustments for multiple comparisons?
  - exact statistical test results, e.g., P values = x but not P values < x;
  - definition of 'center values' as median or average;
  - definition of error bars as s.d. or s.e.m.

Please complete ALL of the questions below.
Select "Not Applicable" only when the requested information is not relevant for your study.

Materials

Newly Created Materials	Information included in the manuscript?	In which section is the information available? (Reagents and Tools Table, Materials and Methods, Figures, Data Availability Section)
New materials and reagents need to be available; do any restrictions apply?	Not Applicable	

Antibodies	Information included in the manuscript?	In which section is the information available? (Reagents and Tools Table, Materials and Methods, Figures, Data Availability Section)
For **antibodies** provide the following information:<br>- Commercial antibodies: RRID (if possible) or supplier name, catalogue number and or/clone number<br>- Non-commercial: RRID or citation	Yes	Detailed methods, Sections 2.2, 2.4

DNA and RNA sequences	Information included in the manuscript?	In which section is the information available? (Reagents and Tools Table, Materials and Methods, Figures, Data Availability Section)
**Short novel DNA or RNA including primers, probes**: provide the sequences.	Not Applicable	

Cell materials	Information included in the manuscript?	In which section is the information available? (Reagents and Tools Table, Materials and Methods, Figures, Data Availability Section)
**Cell lines:** Provide species information, strain. Provide accession number in repository **OR** supplier name, catalog number, clone number, and/**OR** RRID.	Not Applicable	
**Primary cultures:** Provide species, strain, sex of origin, genetic modification status.	Not Applicable	
Report if the cell lines were recently **authenticated** (e.g., by STR profiling) and tested for mycoplasma contamination.	Not Applicable	

Experimental animals	Information included in the manuscript?	In which section is the information available? (Reagents and Tools Table, Materials and Methods, Figures, Data Availability Section)
**Laboratory animals or Model organisms:** Provide species, strain, sex, age, genetic modification status. Provide accession number in repository **OR** supplier name, catalog number, clone number, **OR** RRID.	Yes	Manuscript, Methods Section Page 12
**Animal observed in or captured from the field:** Provide species, sex, and age where possible.	Not Applicable	
Please detail **housing and husbandry conditions**.	Yes	Manuscript, Methods Section Page 12

Plants and microbes	Information included in the manuscript?	In which section is the information available? (Reagents and Tools Table, Materials and Methods, Figures, Data Availability Section)
**Plants:** provide species and strain, ecotype and cultivar where relevant, unique accession number if available, and source (including location for collected wild specimens).	Not Applicable	
**Microbes:** provide species and strain, unique accession number if available, and source.	Not Applicable	

Human research participants	Information included in the manuscript?	In which section is the information available? (Reagents and Tools Table, Materials and Methods, Figures, Data Availability Section)
If collected and within the bounds of privacy constraints report on age, sex and gender or ethnicity for all study participants.	Not Applicable	

Core facilities	Information included in the manuscript?	In which section is the information available? (Reagents and Tools Table, Materials and Methods, Figures, Data Availability Section)
If your work benefited from core facilities, was their service mentioned in the acknowledgments section?	Yes	Manuscript, Acknowledgements Section Page 20

Design

Study protocol	Information included in the manuscript?	In which section is the information available? (Reagents and Tools Table, Materials and Methods, Figures, Data Availability Section)
If study protocol has been **pre-registered, provide DOI in the manuscript**. For clinical trials, provide the trial registration number **OR** cite DOI.	Not Applicable	
Report the **clinical trial registration number** (at ClinicalTrials.gov or equivalent), where applicable.	Not Applicable	

Laboratory protocol	Information included in the manuscript?	In which section is the information available? (Reagents and Tools Table, Materials and Methods, Figures, Data Availability Section)
Provide DOI OR other citation details if **external detailed step-by-step protocols** are available.	Not Applicable	

Experimental study design and statistics	Information included in the manuscript?	In which section is the information available? (Reagents and Tools Table, Materials and Methods, Figures, Data Availability Section)
Include a statement about **sample size** estimate even if no statistical methods were used.	Yes	Supplementary Figures and Video legends, Table S1, Page 43
Were any steps taken to minimize the effects of subjective bias when allocating animals/samples to treatment (e.g. **randomization procedure**)? If yes, have they been described?	Not Applicable	
Include a statement about **blinding** even if no blinding was done.	Not Applicable	Blinding is not applicable to our study because the microscopy imaging and data collection were performed in an unbiased manner.
Describe **inclusion/exclusion criteria** if samples or animals were excluded from the analysis. Were the criteria pre-established?<br><br>If sample or data points were omitted from analysis, report if this was due to attrition or intentional exclusion and provide justification.	Not Applicable	No data were excluded from the analyses.
For every figure, are **statistical tests** justified as appropriate? Do the data meet the assumptions of the tests (e.g., normal distribution)? Describe any methods used to assess it. Is there an estimate of variation within each group of data? Is the variance similar between the groups that are being statistically compared?	Not Applicable	

Sample definition and in-laboratory replication	Information included in the manuscript?	In which section is the information available? (Reagents and Tools Table, Materials and Methods, Figures, Data Availability Section)
In the figure legends: state number of times the experiment was **replicated** in laboratory.	Yes	Supplementary Figures and Video legends, Table S1, Page 43
In the figure legends: define whether data describe **technical or biological replicates**.	Yes	Supplementary Figures and Video legends, Table S1, Page 43

Ethics

Ethics	Information included in the manuscript?	In which section is the information available? (Reagents and Tools Table, Materials and Methods, Figures, Data Availability Section)
Studies involving **human participants**: State details of **authority granting ethics approval** (IRB or equivalent committee(s), provide reference number for approval.	Not Applicable	
Studies involving **human participants**: Include a statement confirming that **informed consent** was obtained from all subjects and that the experiments conformed to the principles set out in the WMA Declaration of Helsinki and the Department of Health and Human Services Belmont Report.	Not Applicable	
Studies involving **human participants:** For publication of **patient photos**, include a statement confirming that consent to publish was obtained.	Not Applicable	
Studies involving experimental **animals**: State details of **authority granting ethics approval** (IRB or equivalent committee(s), provide reference number for approval. Include a statement of compliance with ethical regulations.	Yes	Manuscript, Methods Section Page 12
Studies involving **specimen and field samples:** State if relevant **permits** obtained, provide details of authority approving study; if none were required, explain why.	Not Applicable	

Dual Use Research of Concern (DURC)	Information included in the manuscript?	In which section is the information available? (Reagents and Tools Table, Materials and Methods, Figures, Data Availability Section)
Could your study fall under dual use research restrictions? Please check biosecurity documents and list of **select agents and toxins** (CDC): https://www.selectagents.gov/sat/list.htm	Not Applicable	
If you used a select agent, is the security level of the lab appropriate and reported in the manuscript?	Not Applicable	
If a study is subject to dual use research of concern regulations, is the name of the **authority granting approval and reference number** for the regulatory approval provided in the manuscript?	Not Applicable	

Reporting

The MDAR framework recommends adoption of discipline-specific guidelines, established and endorsed through community initiatives. Journals have their own policy about requiring specific guidelines and recommendations to complement MDAR.

Adherence to community standards	Information included in the manuscript?	In which section is the information available? (Reagents and Tools Table, Materials and Methods, Figures, Data Availability Section)
State if relevant guidelines or checklists (e.g., **ICMJE, MIBBI, ARRIVE, PRISMA**) have been followed or provided.	Not Applicable	
For **tumor marker prognostic studies,** we recommend that you follow the **REMARK** reporting guidelines (see link list at top right). See author guidelines, under 'Reporting Guidelines'. Please confirm you have followed these guidelines.	Not Applicable	
For **phase II and III randomized controlled trials**, please refer to the **CONSORT** flow diagram (see link list at top right) and submit the CONSORT checklist (see link list at top right) with your submission. See author guidelines, under 'Reporting Guidelines'. Please confirm you have submitted this list.	Not Applicable	

Data Availability

Data availability	Information included in the manuscript?	In which section is the information available? (Reagents and Tools Table, Materials and Methods, Figures, Data Availability Section)
Have **primary datasets** been deposited according to the journal's guidelines (see 'Data Deposition' section) and the respective accession numbers provided in the Data Availability Section?	Yes	Manuscript, Data and code availability Page 15
Were **human clinical and genomic datasets** deposited in a public access-controlled repository in accordance to ethical obligations to the patients and to the applicable consent agreement?	Not Applicable	
Are **computational models** that are central and integral to a study available without restrictions in a machine-readable form? Were the relevant accession numbers or links provided?	Yes	Manuscript, Data and code availability Page 15
If publicly available data were reused, provide the respective **data citations in the reference list**.	Not Applicable	
